# Early cave art and ancient DNA record the origin of European bison

**DOI:** 10.1038/ncomms13158

**Published:** 2016-10-18

**Authors:** Julien Soubrier, Graham Gower, Kefei Chen, Stephen M. Richards, Bastien Llamas, Kieren J. Mitchell, Simon Y. W. Ho, Pavel Kosintsev, Michael S. Y. Lee, Gennady Baryshnikov, Ruth Bollongino, Pere Bover, Joachim Burger, David Chivall, Evelyne Crégut-Bonnoure, Jared E. Decker, Vladimir B. Doronichev, Katerina Douka, Damien A. Fordham, Federica Fontana, Carole Fritz, Jan Glimmerveen, Liubov V. Golovanova, Colin Groves, Antonio Guerreschi, Wolfgang Haak, Tom Higham, Emilia Hofman-Kamińska, Alexander Immel, Marie-Anne Julien, Johannes Krause, Oleksandra Krotova, Frauke Langbein, Greger Larson, Adam Rohrlach, Amelie Scheu, Robert D. Schnabel, Jeremy F. Taylor, Małgorzata Tokarska, Gilles Tosello, Johannes van der Plicht, Ayla van Loenen, Jean-Denis Vigne, Oliver Wooley, Ludovic Orlando, Rafał Kowalczyk, Beth Shapiro, Alan Cooper

**Affiliations:** 1 Australian Centre for Ancient DNA, School of Biological Sciences, University of Adelaide, Adelaide, South Australia 5005, Australia; 2 School of Biological Sciences, University of Sydney, Sydney, New South Wales 2006, Australia; 3 Institute of Plant and Animal Ecology, Russian Academy of Sciences, 202 8 Marta Street, 620144 Ekaterinburg, Russia; 4 School of Biological Sciences, Flinders University, South Australia 5001, Australia; 5 Earth Sciences Section, South Australian Museum, North Terrace, Adelaide, South Australia 5000, Australia; 6 Zoological Institute RAS, Universitetskaya Naberezhnaya 1, 199034 St Petersburg, Russia; 7 Palaeogenetics Group, Institute of Anthropology, University of Mainz D-55128, Mainz, Germany; 8 Department of Biodiversity and Conservation, Institut Mediterrani d'Estudis Avançats (CSIC-UIB), Cr. Miquel Marquès 21, 07190 Esporles, Illes Balears; 9 Oxford Radiocarbon Accelerator Unit, Research Laboratory for Archaeology and the History of Art, University of Oxford, Oxford OX1 3QY, UK; 10 Museum Requien, 67 rue Joseph Vernet, 84000 Avignon, France; 11 Laboratoire TRACES UMR5608, Université Toulouse Jean Jaurès - Maison de la Recherche, 5 allée Antonio Machado, 31058 Toulouse, France; 12 Division of Animal Sciences, University of Missouri, Columbia, Missouri 65211, USA; 13 ANO Laboratory of Prehistory, 14 Linia 3e 11, 199034 St Petersburg, Russia; 14 Environment Institute and School of Biological Sciences, University of Adelaide, Adelaide, South Australia 5005, Australia; 15 Dipartimento di Studi Umanistici, Università degli Studi di Ferrara, 12 Via Paradiso, 44121 Ferrara, Italy; 16 CNRS, TRACES, UMR 5608 et CREAP, MSHS Toulouse, USR 3414, Maison de la Recherche, 5 allées Antonio Machado, 31058 Toulouse, France; 17 CERPOLEX/Mammuthus, Anna Paulownastraat 25A, NL-2518 BA Den Haag, The Netherlands; 18 School of Archaeology and Anthropology, Australian National University, Building 14, Canberra, Australian National University 0200, Australia; 19 Max Planck Institute for the Science of Human History, 07745 Jena, Germany; 20 Mammal Research Institute, Polish Academy of Sciences, Waszkiewicza 1c, 17-230 Białowieża, Poland; 21 Department of Archaeology, Centre for the Archaeology of Human Origins, University of Southampton, Avenue Campus, Southampton SO17 1BF, UK; 22 Unité Histoire naturelle de l'Homme préhistorique (UMR 7194), Sorbonne Universités, Muséum national d'Histoire narurelle, CNRS, 1 rue René Panhard, 75013 Paris, France; 23 Department of Stone Age, Institute of Archaeology, National Ukrainian Academy of Science, 04210 Kiev, Ukraine; 24 Institute for Archaeological Sciences, Archaeo and Palaeogenetics, University of Tübingen, 72070 Tübingen, Germany; 25 Palaeogenomics and Bio-Archaeology Research Network, Research Laboratory for Archaeology, Dyson Perrins Building, South Parks Road, Oxford OX1 3QY, UK; 26 School of Mathematical Sciences, The University of Adelaide, Adelaide, South Australia 5005, Australia; 27 Chercheur associé, CREAP, MSHS Toulouse, URS 3414, Maison de la Recherche, 5 allées Antonio Machado, 31058 Toulouse, France; 28 Centre for Isotope Research, Radiocarbon Laboratory, University of Groningen, Nijenborg 4, NI-9747 AG Groningen, The Netherlands; 29 Centre National de la Recherche Scientifique, Muséum National d'Histoire Naturelle, Sorbonne Universités, UMR7209, ‘Archéozoologie, archéobotanique: sociétés, pratiques et environnements', CP56, 55 rue Buffon, 75005 Paris, France; 30 Centre for GeoGenetics, Natural History Museum of Denmark, University of Copenhagen, ØsterVoldgade 5-7, Copenhagen 1350K, Denmark; 31 Université de Toulouse, University Paul Sabatier, Laboratoire AMIS, CNRS UMR 5288, 37 Allées Jules Guesde, Toulouse 31000, France; 32 Department of Ecology and Evolutionary Biology, University of California Santa Cruz, 1156 High Street, Santa Cruz, California 95064, USA; 33 UCSC Genomics Institute, University of California Santa Cruz, 1156 High Street, Santa Cruz, California 95064, USA

## Abstract

The two living species of bison (European and American) are among the few terrestrial megafauna to have survived the late Pleistocene extinctions. Despite the extensive bovid fossil record in Eurasia, the evolutionary history of the European bison (or wisent, *Bison bonasus*) before the Holocene (<11.7 thousand years ago (kya)) remains a mystery. We use complete ancient mitochondrial genomes and genome-wide nuclear DNA surveys to reveal that the wisent is the product of hybridization between the extinct steppe bison (*Bison priscus*) and ancestors of modern cattle (aurochs, *Bos primigenius*) before 120 kya, and contains up to 10% aurochs genomic ancestry. Although undetected within the fossil record, ancestors of the wisent have alternated ecological dominance with steppe bison in association with major environmental shifts since at least 55 kya. Early cave artists recorded distinct morphological forms consistent with these replacement events, around the Last Glacial Maximum (LGM, ∼21–18 kya).

## Abstract (web summary)

The ancestry of the European bison (wisent) remains a mystery. Here, Cooper and colleagues examine ancient DNA from fossil remains of extinct bison, and reveal the wisent originated through the hybridization of the extinct Steppe bison and ancestors of modern cattle.

## Graphical abstract

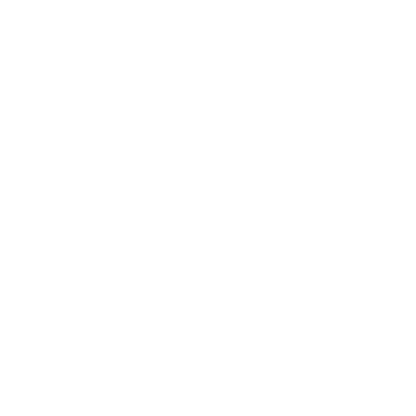

The extensive Late Pleistocene fossil record of bovids in Europe consists of two recognized forms: the aurochs (*Bos primigenius*), ancestor of modern cattle, and the mid/late Pleistocene ‘steppe bison' (*Bison priscus*), which also ranged across Beringia as far as western Canada^1^,^2^. The European bison, or wisent (*Bison bonasus*), has no recognized Pleistocene fossil record and seems to suddenly appear in the early Holocene (<11.7 kya)^3^,^4^, shortly after the disappearance of the steppe bison during the megafaunal extinctions of the Late Pleistocene^5^,^6^,^7^. The Holocene range of wisent included all lowlands of Europe, and several highland areas of eastern Europe (where it was termed the Caucasian form *B. bonasus caucasicus*) but range reduction and hunting by humans brought the species close to extinction, with modern populations descending from just 12 mostly Polish individuals that lived in the 1920s (refs 8, 9). Nuclear DNA sequences and the morphology of the wisent show close similarities to American bison (*B. bison*), but wisent mitochondrial DNA (mtDNA) indicates a closer relationship with cattle. This suggests some form of introgression from cattle or a related *Bos* species^10^,^11^,^12^, potentially associated with the recent extreme bottleneck event.

Both aurochs and bison feature heavily in Palaeolithic cave art, with 820 depictions displaying bison individuals (∼21% of known cave ornamentation^13^). The diversity of bison representations has been explained as putative cultural and individual variations of style through time, since the steppe bison was assumed to be the only bison present in Late Paleolithic Europe^14^,^15^,^16^. However, two distinct morphological forms of bison (Fig. 1, Supplementary Information section) are clearly apparent in cave art: a long-horned form similar to modern American bison (which are thought to be descended from steppe bison), with very robust forequarters and oblique dorsal line, and a second form with thinner double-curved horns, smaller hump and more balanced body proportions, similar to wisent. The former is abundant in art older than the Last Glacial Maximum (LGM, ∼22–18 kya), while the latter dominates Magdalenian art (∼17–12 kya, see Supplementary Information section). Similarly, two distinct morphological forms of Late Pleistocene bison have been reported from North Sea sediments^17^.

To further examine the potential existence of a previously unrecognized fossil bison species within Europe, we sequenced ancient mtDNA and nuclear DNA from bones and teeth of 64 Late Pleistocene/Holocene bison specimens.

We reveal that the wisent lineage originated from hybridization between the aurochs and steppe bison, and this new form alternated ecologically with steppe bison throughout the Late Pleistocene and appears to have been recorded by early cave artists.

## Results

### New group of ancient European bison

The mtDNA sequences of 38 specimens, dated from >50 to 14 kya and ranging from the Caucasus, Urals, North Sea, France and Italy, formed a previously unrecognized genetic clade, hereafter referred to as CladeX, related to modern and historical wisent (including the Caucasian form; Fig. 2a,b). By using the radiocarbon-dated specimens to calibrate our phylogenetic estimate of the timescale, we inferred that the divergence between CladeX and modern wisent lineages occurred ∼120 (92–152) kya, likely during the last (Eemian) interglacial. Both these mitochondrial clades are more closely related to cattle than to bison, suggesting that they are descended from an ancient hybridization event that took place >120 kya (presumably between steppe bison and an ancestral form of aurochs, from which the mitochondrial lineage was acquired).

### Hybrid origin of wisent and ancient European bison

To investigate the potential hybrid origins of wisent and CladeX, we used target enrichment and high-throughput methods to sequence ∼10,000 genome-wide bovine single-nucleotide polymorphisms (SNPs) from nine members of CladeX, an ancient (>55 kyr) and a historical (1911 AD) wisent specimen and two steppe bison (30 and >50 kyr). Principal Component Analysis (PCA) and phylogenetic analysis (Fig. 3 and Supplementary Fig. 10) of the nuclear data demonstrate that members of CladeX are closely related to the steppe bison. D-statistic^18^ analyses confirm a closer affinity of both CladeX and the ancient wisent to steppe bison than to modern wisent (Fig. 3b), which is explicable because of rapid genetic drift during the severe bottleneck leading to modern wisent. Concordantly, our historical wisent sample (Caucasian, from 1911) displays a signal intermediate between modern wisent and both CladeX and steppe bison (Fig. 3b(3).

The nuclear and mitochondrial analyses together suggest that the common ancestor of the wisent and CladeX mitochondrial lineages originated from asymmetrical hybridization (or sustained introgression) between male steppe bison and female aurochs (see Supplementary Fig. 20). This scenario is consistent with the heavily polygynous mating system of most large bovids^19^, and the observation that hybridization between either extant bison species and cattle usually results in F1 male infertility, consistent with Haldane's Rule of heterogametic crosses^20^,^21^,^22^. However, it is unclear whether hybridization took place only once or multiple times, and how and at what point after the initial hybridization event(s) the wisent–CladeX forms became distinct from the steppe bison.

To examine the extent of genetic isolation maintained through time by the hybrid forms (wisent and CladeX) from steppe bison, we characterized the genomic signals originating from either steppe bison or aurochs in the wisent and CladeX lineages. Calculations of *f*_4_ ratios^23^ show the same high proportion of nuclear signal from steppe bison (≥89.1%) and low proportion from aurochs (≤10.9%) in both wisent and CladeX (Fig. 3d and Supplementary Table 6). Independent calculation of hybridization levels from ABC comparisons with simulated data also shows clear evidence of hybridization, with similar proportions of nuclear signal (97.2% probability that there is at least 1% aurochs ancestry and a 87.6% probability that there is at least 5% aurochs ancestry; see Supplementary Note 2 and Supplementary Tables 10 and 11). The agreement between these two methods is compelling evidence of hybridization. In addition, a greater number of derived alleles are common to both wisent and CladeX lineages (either from the imprint of steppe bison ancestry, aurochs ancestry, or from post-hybridization drift) than expected from multiple hybridization events (see Supplementary Note 2 and Supplementary Tables 8 and 9), implying that CladeX represents part of the Late Pleistocene wisent diversity. The age of the oldest genotyped specimens of CladeX (23 kyr) and wisent (>55 kyr) confirm that the initial hybridization event (or ultimate significant introgression of steppe bison) occurred before 55 kya. Together, the long-term stability of the nuclear and mitochondrial signal in wisent and CladeX indicates that the hybrid bison lineage maintained a marked degree of genetic isolation throughout the Late Pleistocene, consistent with the different morphologies observed in the North Sea specimens^17^.

### Hybrid and steppe Bison represent different ecological forms

The temporal distribution of genotyped individuals reveals that wisent mitochondrial lineages (including CladeX) are only observed before 50 kya and after 34 kya, when steppe bison appears to be largely absent from the European landscape (Fig. 4). The detailed records of the southern Ural sites allow the timing of the population replacements between steppe bison and wisent to be correlated with major palaeoenvironmental shifts, revealing that the wisent was associated with colder, more tundra-like landscapes and absence of a warm summer (Supplementary Fig. 22). Stable isotope data (∂^13^C/∂^15^N; Supplementary Fig. 23) and environment reconstructions show that wisent were present in a more diverse environment than steppe bison, with a more variable diet, suggesting that these two taxa occupied separate ecological niches.

## Discussions

Contrary to previous palaeontological interpretations, the ancestors of modern wisent were present in Europe throughout the Late Pleistocene, and the two different bison morphs depicted in Paleolithic art suggest that early artists recorded the replacement of the steppe bison by the hybrid form (including CladeX) in Western Europe around the LGM. Two bison individuals have been genotyped from European caves during this period: a 19-kyr-old steppe bison from Southern France^24^ and a 16-kyr-old wisent (CladeX) from Northern Italy (present study), corresponding to the timing of the morphological transition from steppe bison-like to wisent-like morphotypes apparent in cave art.

Combined evidence from genomic data, paleoenvironmental reconstructions and cave paintings strongly suggest that the hybridization of steppe bison with an ancient aurochs lineage during the late Pleistocene led to a morphologically and ecologically distinct form, which maintained its integrity and survived environmental changes on the European landscape until modern times. Although further analyses of deeper ancient genome sequencing will be necessary to characterize the phenotypic consequences of such hybridization, this adds to recent evidence of the importance of hybridization as a mechanism for speciation and adaptation of mammals^25^,^26^,^27^,^28^,^29^ as is already accepted for plants. Lastly, the paraphyly of *Bos* with respect to *Bison*, and the evidence of meaningful hybridization between aurochs and bison, support the argument that both groups should be combined under the genus *Bos*^12^,^19^,^30^.

## Methods

### Ancient DNA samples description and processing

Samples from a total of 87 putative bison bones were collected from three regions across Europe: Urals, Caucasus and Western Europe (Supplementary Data 1).

Dating of 45 samples that yielded DNA was performed at the Oxford Radiocarbon Accelerator Unit of the University of Oxford (OxA numbers), and the Ångström Laboratory of the University of Uppsala, Sweden, for the Swiss sample (Ua-42583). The calibration of radiocarbon dates was performed using OxCal v4.1 with the IntCal13 curve^31^ (Supplementary Data 1).

All ancient DNA work was conducted in clean-room facilities at the University of Adelaide's Australian Centre for Ancient DNA, Australia (ACAD), and at the University of Tuebingen, Germany (UT) following the published guidelines^32^.

Samples were extracted using either phenol–chloroform^33^ or silica-based methods^34^,^35^ (see Supplementary Data 1).

Mitochondrial control region sequences (>400 bp) were successfully amplified from 65 out of 87 analysed samples in one or up to four overlapping fragments, depending on DNA preservation^33^. To provide deeper phylogenetic resolution and further examine the apparent close relationship between *Bos* and wisent mitochondria, whole-mitogenome sequences of 13 CladeX specimens, as well as one ancient wisent, one historical wisent and one steppe bison were generated using hybridization capture with either custom-made^36^,^37^ (see Supplementary Note 1 for details).

In addition, genome-wide nuclear locus capture was attempted on DNA extracts from 13 bison samples (see Supplementary Table 2), using either an ∼40,000 or an ∼10,000 set of probes (as described in Supplementary Note 1). All targeted loci were part of the BovineSNP50 v2 BeadChip (Illumina) bovine SNP loci used in a previous phylogenetic study^38^. Ultimately, only the 9,908 loci common to both sets were used for comparative analysis.

### Genetic data analysis

*Data processing*. Next-generation sequencing data were obtained from enriched libraries using paired-end reactions on Illumina HiSeq or MiSeq machines, and processed using the pipeline Paleomix v1.0.1 (ref. 39). AdapterRemoval v2 (ref. 40) was used to trim adapter sequences, merge the paired reads and eliminate all reads shorter than 25 bp. BWA v0.6.2 was then used to map the processed reads to either the reference mitochondrial genome of the wisent (NC_014044), American bison (NC_012346—only for the steppe bison A3133) or the *Bos taurus* genome reference UMD 3.1 (ref. 41). Minimum mapping quality was set at 25, seeding was disabled and the maximum number of gap opens was set to 2 (see Supplementary Tables 2 and 3).

MapDamage v2 (ref. 42) was used to check that the expected contextual mapping and damage patterns were observed for each library, depending on the enzymatic treatment used during library preparation (see Supplementary Table 3 and Supplementary Figs 1–3 for examples), and to rescale base qualities accordingly.

**Figure 1:**
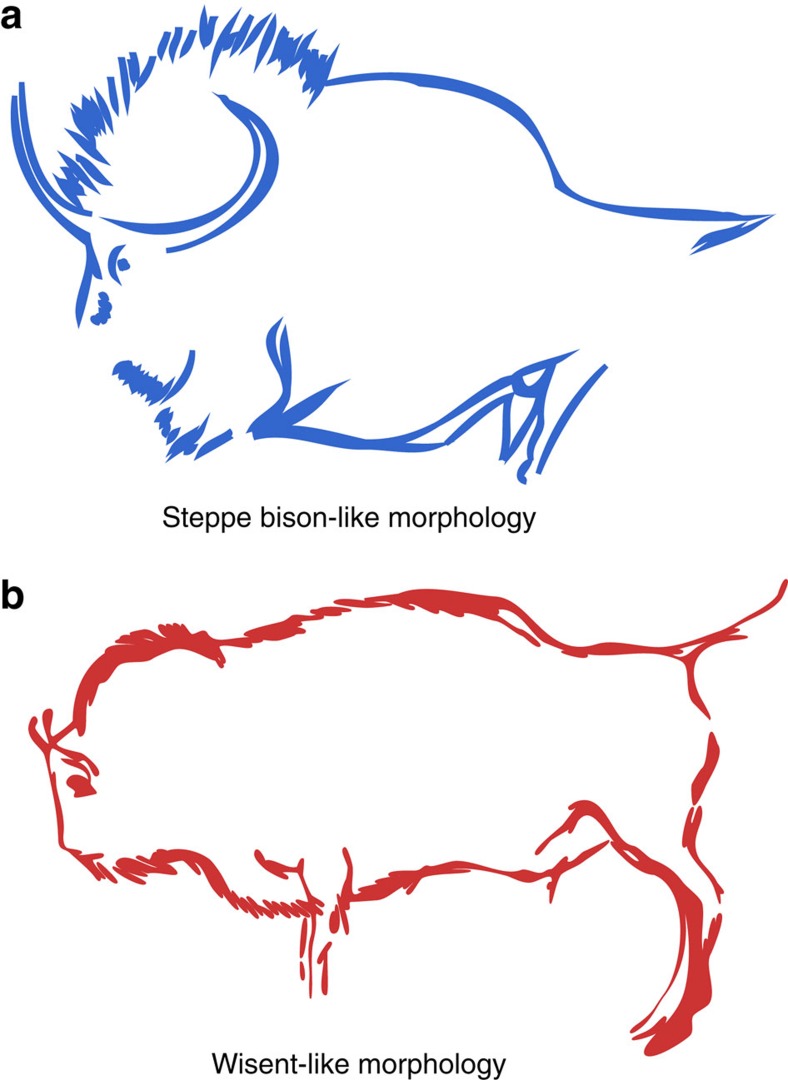
Cave painting example of steppe bison-like and wisent-like morphs. (**a**) Reproduction from Lascaux cave (France), from the Solutrean or early Magdalenian period (∼20,000 kya—picture adapted from ref. 53). (**b**) Reproduction from the Pergouset cave (France), from the Magdalenian period (<17,000 kya—picture adapted from ref. 54).

**Figure 2:**
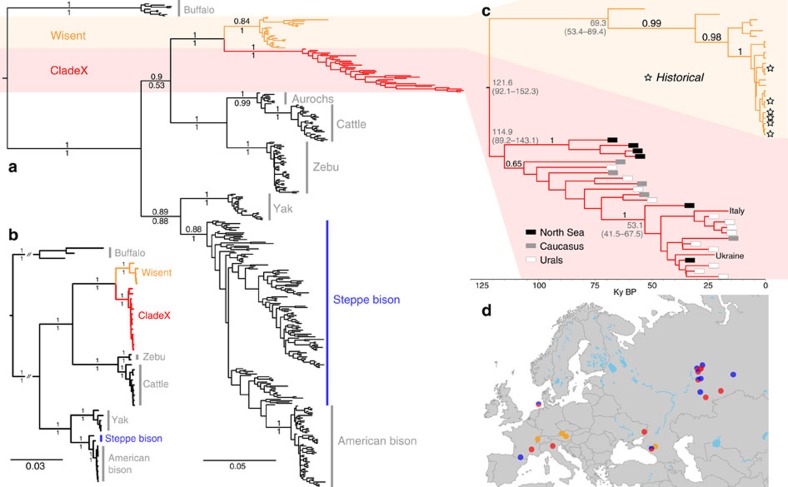
Identification of CladeX. (**a**) Phylogenetic tree inferred from bovine mitochondrial control region sequences, showing the new clade of bison individuals. The positions of the newly sequenced individuals are marked in red for CladeX. (**b**) Bovine phylogeny estimated from whole-mitochondrial genome sequences, showing strong support for the grouping of wisent and CladeX with cattle (cow) and zebu. For both trees (**a**,**b**) numbers above branches represent the posterior probabilities from Bayesian inference, numbers below branches represent approximate likelihood ratio test support values from maximum-likelihood analysis and scale bars represent nucleotide substitutions per site from the Bayesian analysis. (**c**) Maximum-clade-credibility tree of CladeX and wisent estimated using Bayesian analysis and calibrated with radiocarbon dates associated with the sequenced bones. Dates of samples older than 50 kyr were estimated in the phylogenetic reconstruction. (**d**) Map showing all sampling locations, using the same colour code (red for CladeX, orange for wisent and blue for steppe bison).

*Phylogenetic analyses*. The 60 newly sequenced bovine mitochondrial regions (Supplementary Data 1) were aligned with 302 published sequences (Supplementary Table 4), and a phylogenetic tree was inferred using both maximum-likelihood (PhyML v3 (ref. 43)) and Bayesian (MrBayes v3.2.3 (ref. 44)) methods (Fig. 2a and Supplementary Fig. 4). The same methods were used to obtain the whole-mitogenome phylogeny of 16 newly sequenced bison (Supplementary Data 1) aligned with 31 published sequences (Fig. 2b and Supplementary Fig. 5). To estimate the evolutionary timescale, we used the programme BEAST v1.8.1 (ref. 45) to conduct a Bayesian phylogenetic analysis of all radiocarbon-dated samples from CladeX and wisent (Fig. 1c), using the mean calibrated radiocarbon dates as calibration points. All parameters showed sufficient sampling after 5,000,000 steps, and a date-randomization test supported that the temporal signal from the radiocarbon dates associated with the ancient sequences was sufficient to calibrate the analysis^46^ (Supplementary Fig. 6).

Finally, phylogenetic trees were inferred from nuclear loci data using RAxML v8.1.21 (ref. 47), first from published data of modern bovine representatives^38^ (using sheep as an outgroup; Supplementary Fig. 7) and then including five ancient samples (two ancient steppe bison, an ancient wisent, a historical wisent and a CladeX bison; Fig. 2a), which had the highest number of nuclear loci successfully called among the ∼10 k nuclear bovine SNPs targeted with hybridization capture (see Supplementary Fig. 8).

**Figure 3:**
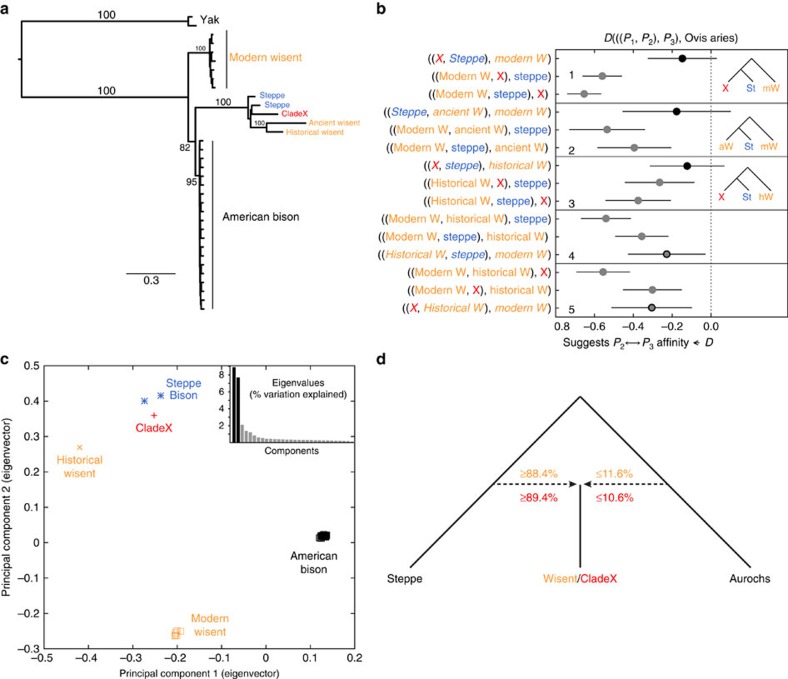
Genome-wide data comparison of bison. (**a**) Maximum-likelihood phylogeny of modern and ancient bison from ∼10,000 genome-wide nuclear sites, showing the close relationship between CladeX and steppe bison. However, a bifurcating phylogeny is not capable of displaying the complex relationships between these taxa (see Supplementary Fig. 8). Numbers above branches represent bootstrap values. (**b**) D-statistics from the same ∼10,000 nuclear sites, using sheep as outgroup. For three bison populations, assuming two bifurcations and no hybridizations, three possible phylogenetic topologies can be evaluated using D-statistics, with the value closest to 0, indicating which topology is the most parsimonious. The topology being tested is shown on the vertical axis. Error bars are three s.e.'s (from block jackknife) either side of the data point. Data points that are significantly different from zero are shown in grey. The data point representing the topology in **a**, among a set of three possible topologies, is shown with a black outline. (**c**) Principal Component Analysis of ∼10,000 genome-wide nuclear sites (ancient wisent not included due to the sensitivity of PCA to missing data, see Supplementary Fig. 10). (**d**) Proportion of steppe bison and aurochs ancestry in both wisent and CladeX lineages, calculated with *f_4_* ratios.

*Principal Component Analysis*. PCA (Fig. 3a and Supplementary Fig. 10) was performed using EIGENSOFT version 6.0.1 (ref. 48). In Fig. 3a, CladeX sample A006 was used as the representative of CladeX, as this sample contained the most complete set of nuclear loci called at the bovine SNP loci (see Supplementary Table 2). Other CladeX individuals, as well as ancient wisent, cluster towards coordinates 0.0, 0.0 (see Supplementary Fig. 10), because of missing data.

*D and f statistics*. Support for the bifurcating nuclear tree (Fig. 2a) was further tested using D-statistics calculated using ADMIXTOOLS version 3.0, git∼3065acc5 (ref. 23). Sensitivity to factors like sampling bias, depth of coverage, choice of outgroup, heterozygosity (by haploidization) and missing data did not have notable influences on the outcome (Supplementary Figs 12–15).

The proportion of the wisent's ancestry differentially attributable to the steppe bison, and the aurochs was estimated with AdmixTools using an *f_4_* ratio^23^ with sheep (*Ovis aries*) as the outgroup (Supplementary Figs S16, S17 and 3D). Again, the test was shown to be robust to haploidization.

Finally, to test whether the wisent lineages (including CladeX) have a common hybrid ancestry, or whether multiple independent hybridization events gave rise to distinct wisent lineages (Supplementary Fig. 18), we identify nuclear loci that have an ancestral state in the aurochs lineage, but a derived state in the steppe bison lineage (see Supplementary Note 2 section ‘Identification of Derived Alleles'). Hypergeometric tests (Supplementary Tables 8 and 9) showed strong support for an ancestral hybridization event occurring before the divergence of the wisent lineages.

*Testing admixture using ABC and simulated data*. Admixture proportions were also independently tested using simulated data and an ABC approach. Nuclear genetic count data were simulated for two species trees (as described in Supplementary Fig. 19 and Supplementary Note 2 section) by drawing samples from two Multinomial distributions, where for tree topology X_1_, 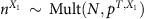, and for tree topology 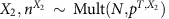. The linear combination of these counts was then considered.

ABC was performed using the R package ‘abc', with a ridge regression correction for comparison of the simulated and observed data using the ‘abc' function^49^. The distance between the observed and simulated data sets is calculated as the Euclidean distance in a three-dimensional space, corrected for the within dimension variability. A tolerance 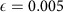 was chosen so that the closest 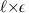 simulated data sets are retained. For each analysis we had 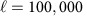, resulting in 500 posterior samples.

We performed leave-one-out cross-validation using the function ‘cv4abc' on 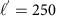 randomly selected simulations, and report the prediction error, calculated as


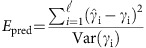


for each analysis. At most, the prediction error was 0.5111 s.d.'s away from zero, and so we observe that the analysis has performed well (see Supplementary Table 10).

### Palaeoenvironment reconstruction and stable isotope analyses

**Figure 4:**
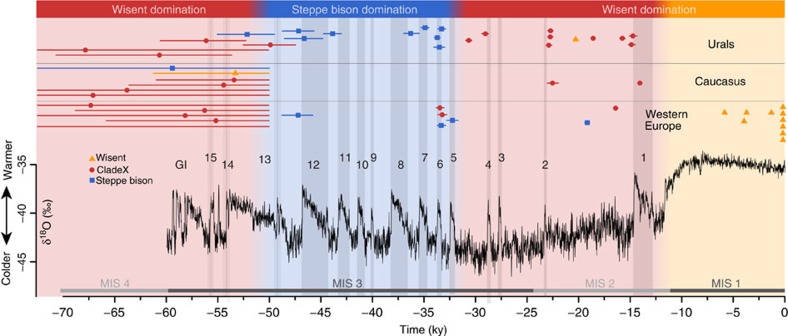
Temporal and geographical distribution of bison in Europe. Individual calibrated AMS dates from the present study and published data are plotted on top of the NGRIP δ^18^O record^55^. Age ranges for infinite AMS dates are from molecular clock estimates (Fig. 2c). Greenland interstadials (GIs) are numbered in black and marine isotope stages (MIS) in grey.

The Urals material has the most complete sampling through time (Fig. 4 and Supplementary Fig. 22), allowing us to contrast reconstructed paleoenvironmental proxies for the region (see Supplementary Note 3). Paleovegetation types were inferred for a convex hull of the Ural study region based on geo-referenced site locations for all genotyped ancient samples (Supplementary Fig. 21). Global maps of BIOME4 plant functional types^50^ were accessed for 2,000-year time steps throughout the period from 70,000 years ago to the present day, with a 1° × 1° latitude/longitude grid cell resolution. We also generated estimates of the annual mean daily temperature and Köppen–Geiger climate classification^51^ using the Hadley Centre Climate model (HadCM3)^52^. Finally, stable isotope values (δ13C and δ15N) obtained for all the genotyped bison individuals from the Ural region were compared between steppe bison and wisent (Supplementary Fig. 23).

### Cave paintings

Two consistent morphological types can be distinguished within the diversity of bison representations (see Fig. 1 and Supplementary Figs 24–27). The first type, abundant before the LGM, is characterized by long horns (with one curve), a very oblique dorsal line and a very robust front part of the body (solid shoulders versus hindquarters), all traits similar to the modern American bison. The second type, dominating the more recent paintings between 18 and 15 kya, displays thinner sinuous horns (often with a double curve), a smaller hump and more balanced dimensions between the front and rear of the body, similar to modern wisent and to some extent aurochsen (see also Supplementary Note 4). The coincident morphological and genetic replacement indicate that variation in bison representations in Paleolithic art does not simply represent stylistic evolution, but actually reflects the different forms of bison genotyped in this study (that is, pre and post-hybridization) through time.

### Data Availability

All newly sequenced mitochondrial control regions are deposited at the European Nucleotide Archive under the following accession numbers (LT599586–645) and all complete mitochondrial genomes at GenBank (KX592174–89). The BEAST input file (XML) is available as Supplementary Data set 2, the MrBayes input file (Nexus), including all whole-mitochondrial genomes, as Supplementary Data set 3 and the nuclear SNPs as Supplementary Data set 4 (VCF format). All other data are included in the Supplementary Material or available upon request to the corresponding authors.

## Additional information

**How to cite this article**: Soubrier, J. *et al*. Early cave art and ancient DNA record the origin of European bison. *Nat. Commun.* **7**, 13158 doi: 10.1038/ncomms13158 (2016).

## Supplementary Material

Supplementary Information Supplementary Figures 1-27, Supplementary Tables 1-11, Supplementary Notes 1-4 and Supplementary References

Supplementary Data 1 Sample details

Supplementary Data 2 BEAST input file

Supplementary Data 3 MrBayes output tree

Supplementary Data 4 Nuclear SNPs
