# Supplementary material for: Early cave art and ancient DNA record the origin of European bison
Source: Nat Commun. 2016 Oct 18;7:13158. doi: 10.1038/ncomms13158 (PMC5071849; doi:10.1038/ncomms13158)
Supplement: Supplementary Information — Supplementary Figures 1-27, Supplementary Tables 1-11, Supplementary Notes 1-4 and Supplementary References [file ncomms13158-s1.pdf]

Supplementary Figures

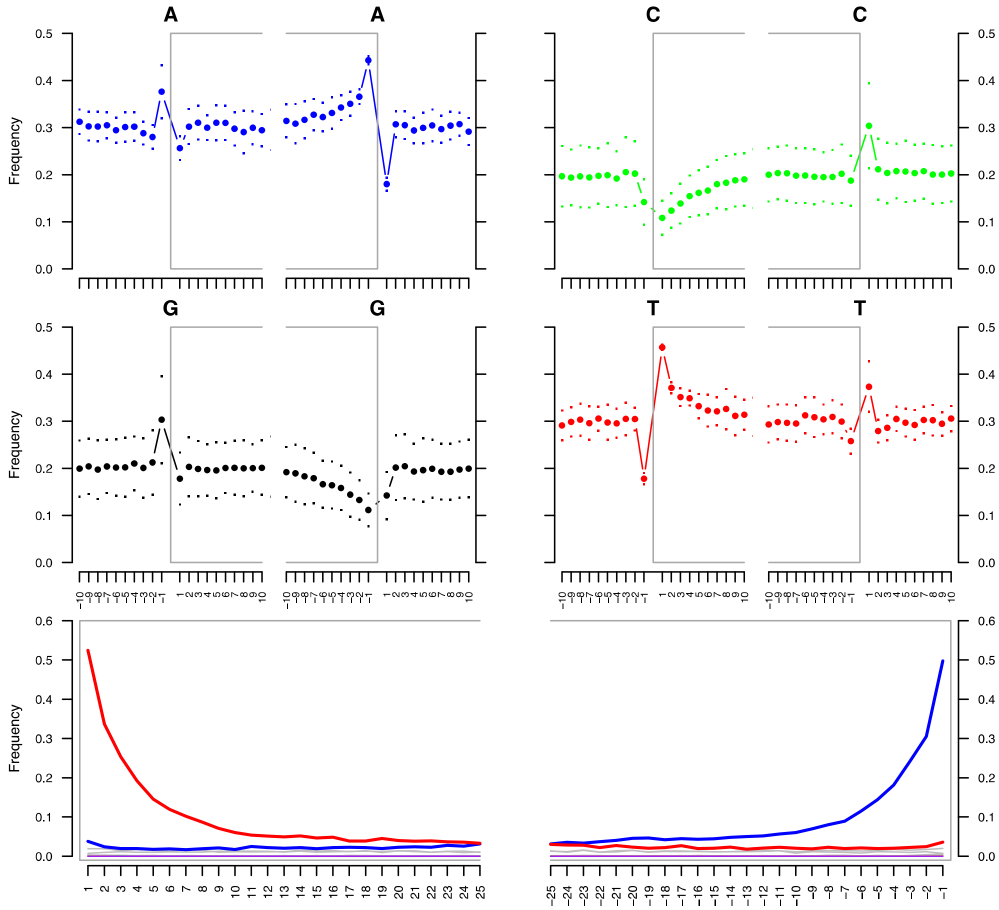

Supplementary Fig 1. Example of damage profile (sample LE257) obtained after sequencing of the whole mitochondrial genome using no treatment for the library preparation. As expected, there is an excess of purines found at the genomic position preceding the mapped reads, and an excess of C>T transitions at the first few positions of the reads.

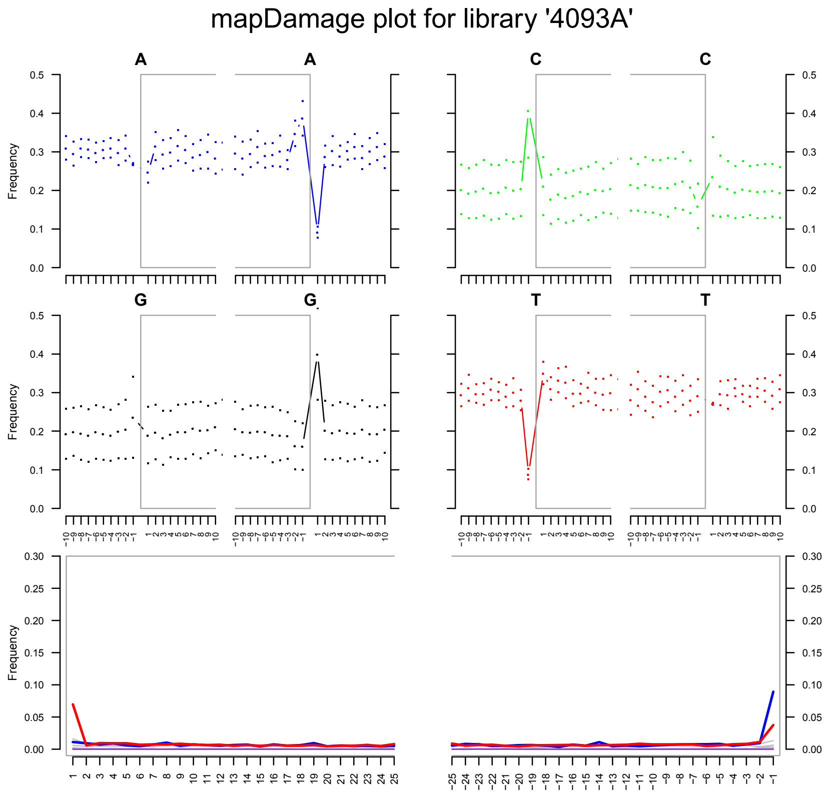

Supplementary Fig 2. Example of damage profile (sample A4093) obtained after sequencing of the whole mitochondrial genome using UDG-half treatment for the library preparation. As expected, there is an excess of cytosine found at the genomic position preceding the mapped reads, and an excess of C>T (and complementary G>A) transitions at the first (last) position of the reads.

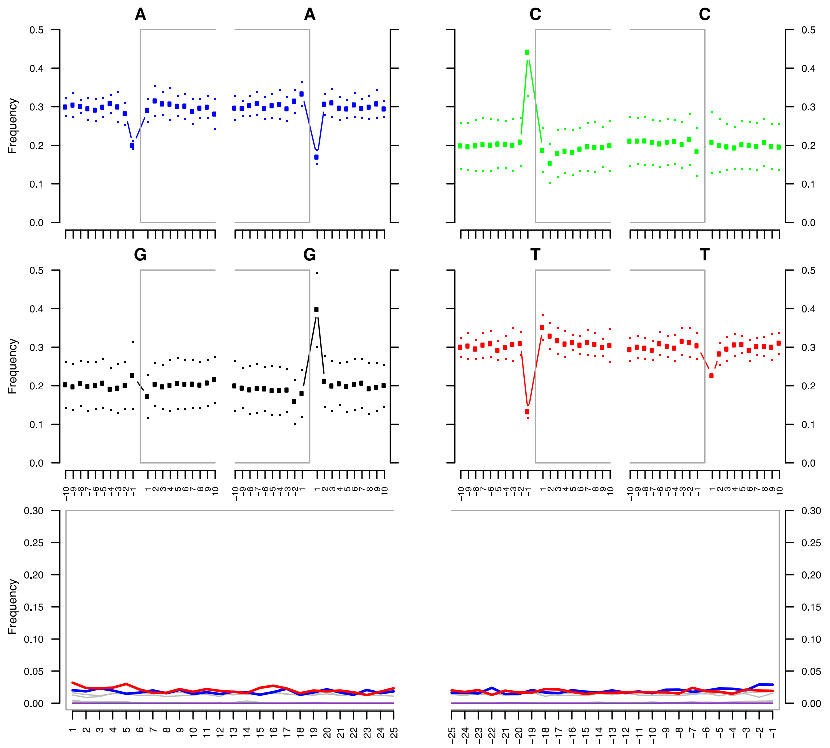

Supplementary Fig 3. Example of damage profile (sample A18) obtained after sequencing of the whole mitochondrial genome using full USER treatment for the library preparation. As expected, there is an excess of cytosine found at the genomic position preceding the mapped reads, and no excess of C>T transitions at the start of the reads.

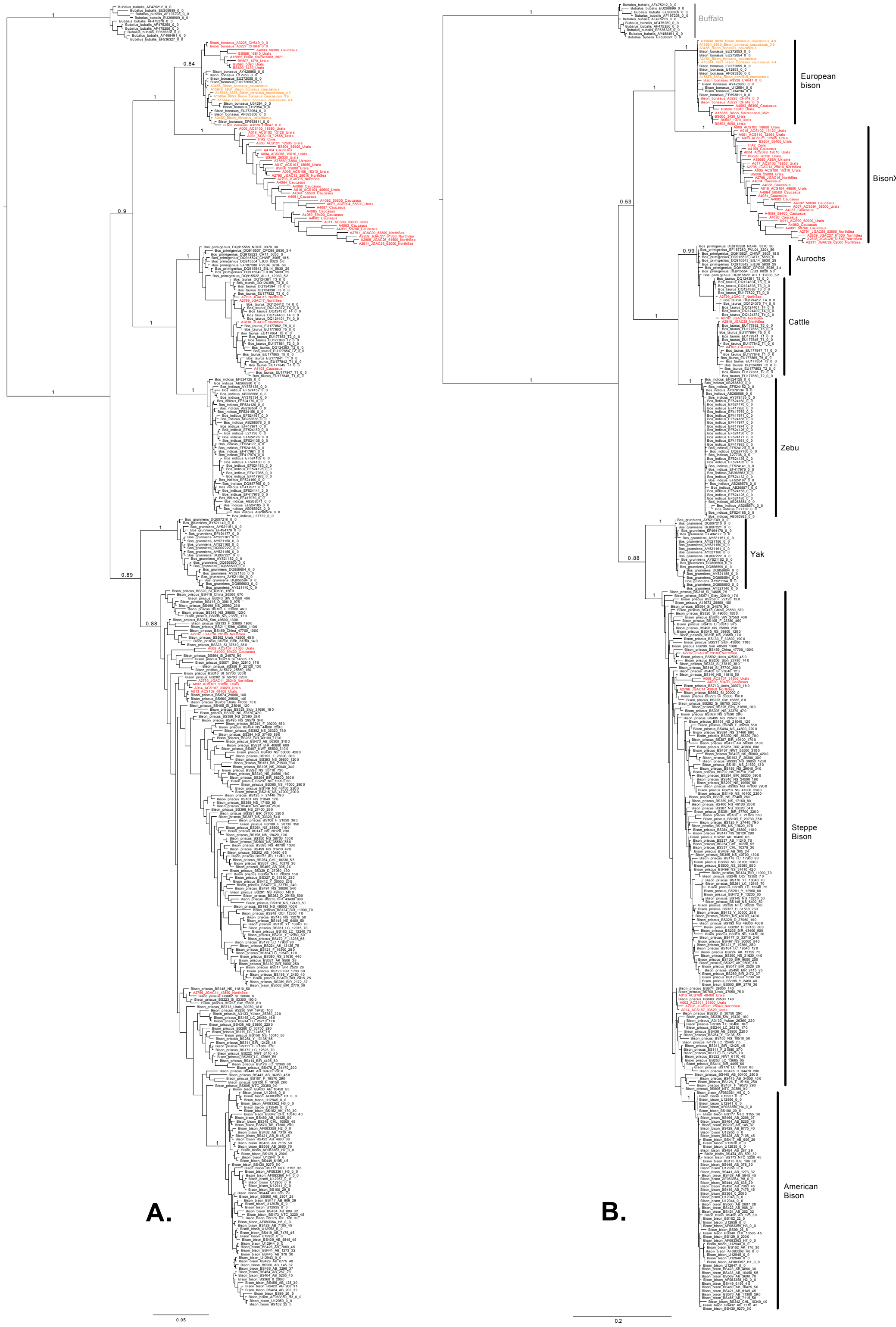

Supplementary Fig 4. Phylogenetic trees of mitochondrial control region sequences from 362 bovid samples. **A.** Majority-rule consensus tree from MrBayes. **B.** Maximum-likelihood tree from PhyML. The 60 newly sequenced individuals are in red font, with the Caucasian bison (*B. bonasus caucasicus*) in orange. Scale bars are given in substitutions per site.

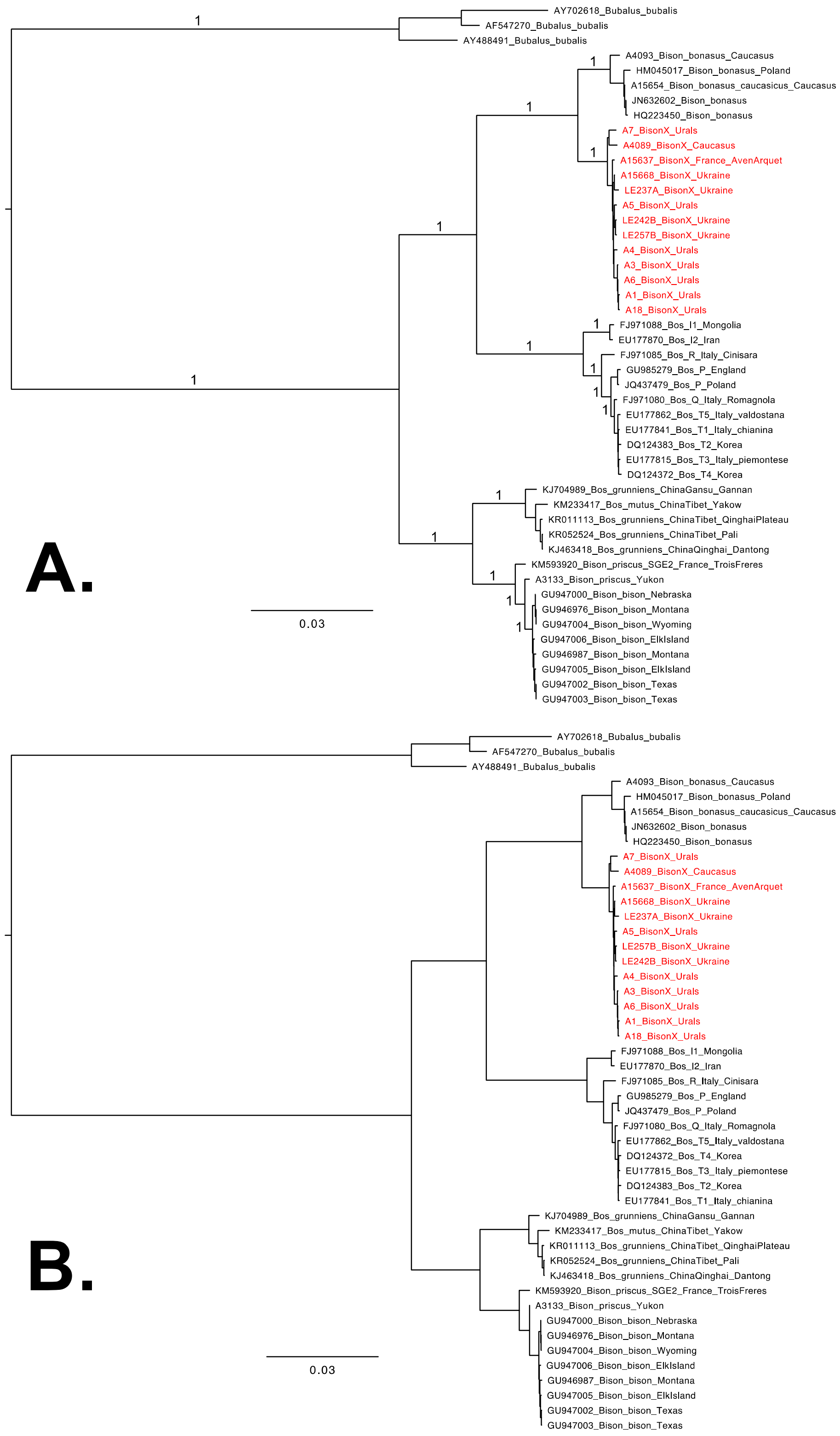

Supplementary Fig 5. Phylogenetic trees inferred from whole mitochondrial genomes. **A.** Majority-rule consensus tree from MrBayes. **B.** Maximum-likelihood tree from PhyML. CladeX bison individuals are colored in red. Scale bars are given in substitutions per site.

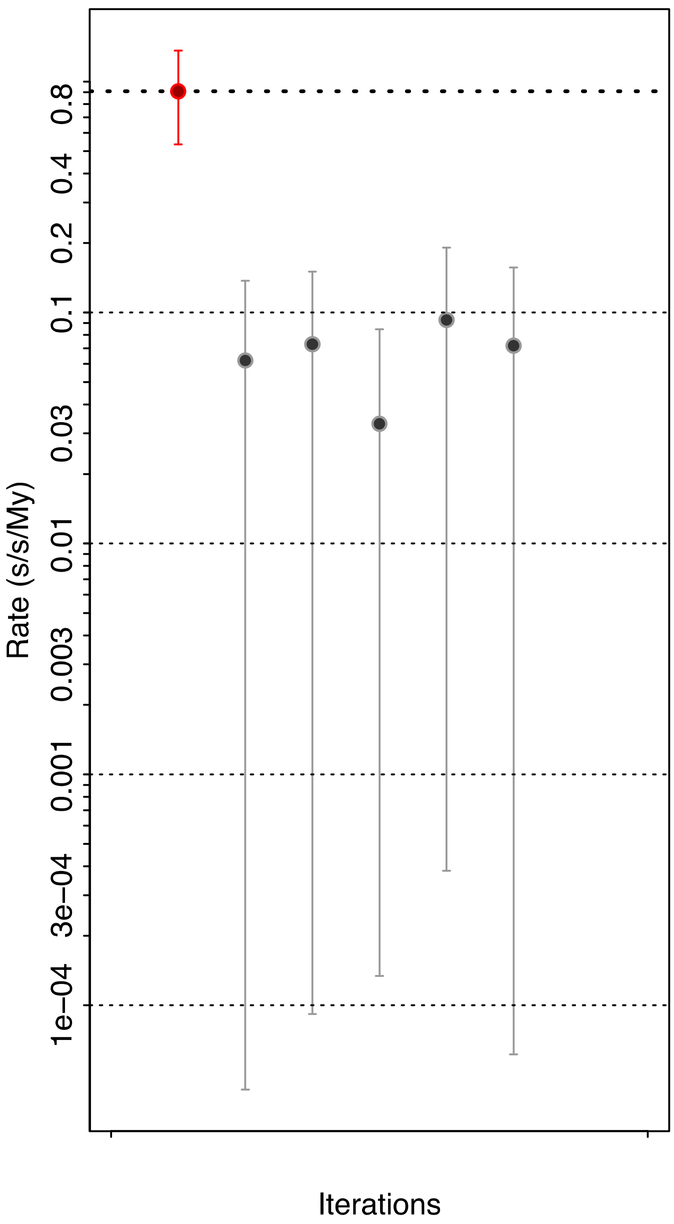

Supplementary Fig 6. Date-randomization test. The red circle and dotted line represent the mean estimate of the molecular rate obtained in the phylogenetic analysis of wisent and CladeX, calibrated using the radiocarbon dates associated with the ancient sequences. The grey lines represent the 95% HPD intervals of rates estimated with randomized dates. None of these margins overlap with the mean rate estimate from the original data set, demonstrating that the radiocarbon dates used for this study contain sufficient temporal information for calibrating the molecular clock.

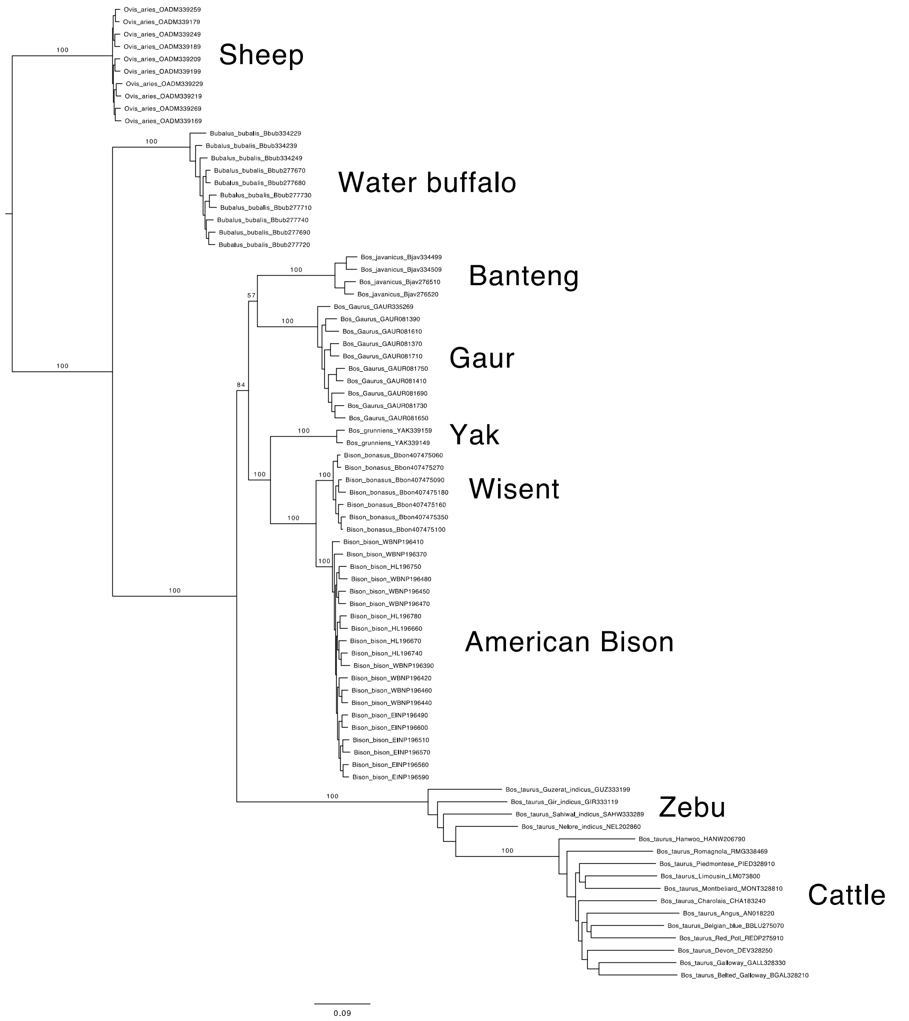

Supplementary Fig 7. Maximum-likelihood phylogeny of modern bovid species (and sheep as outgroup) from ~40k nuclear loci.

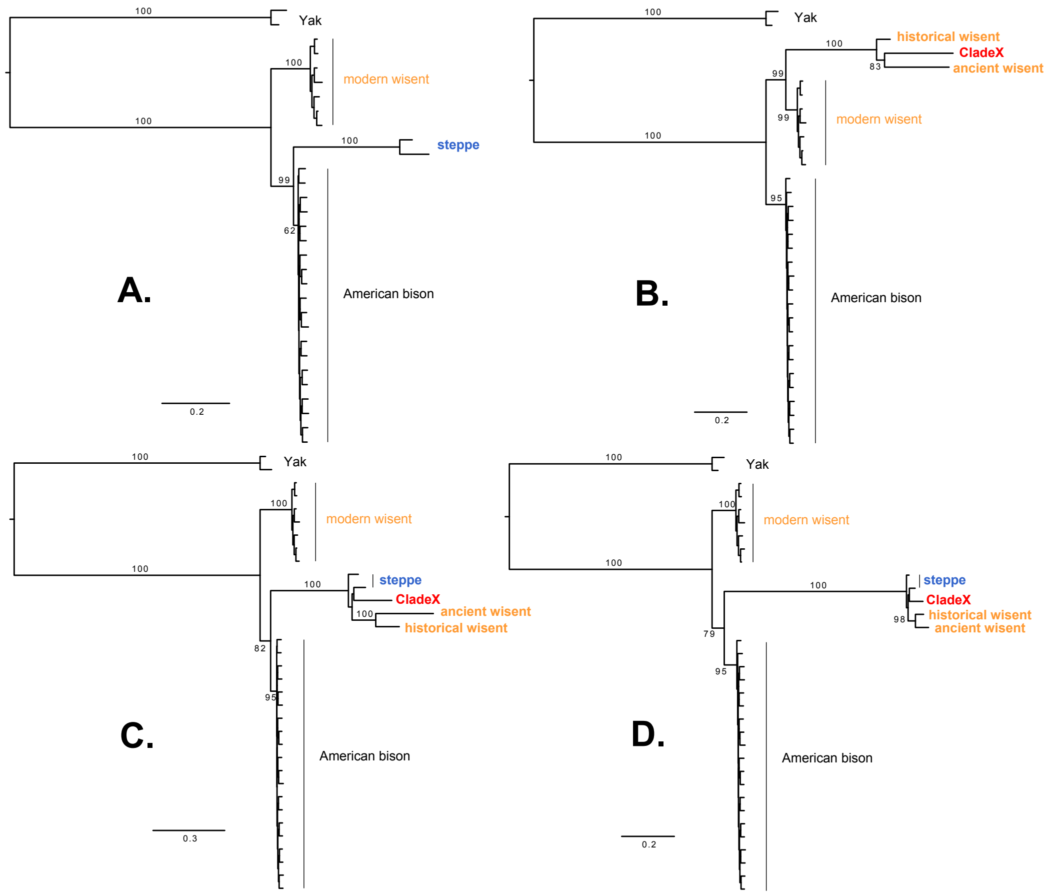

Supplementary Fig 8. Maximum-likelihood phylogenies of modern and ancient bison (and yak as outgroup), from ~10k nuclear loci. **A.** Phylogeny including the two ancient steppe bison. **B.** Phylogeny including the three pre-modern wisent. **C.** Phylogeny including the two steppe bison and three pre-modern wisent (ancient, historical and CladX). **D.** Replicate of **C.** but only using transversions for the non-modern samples.

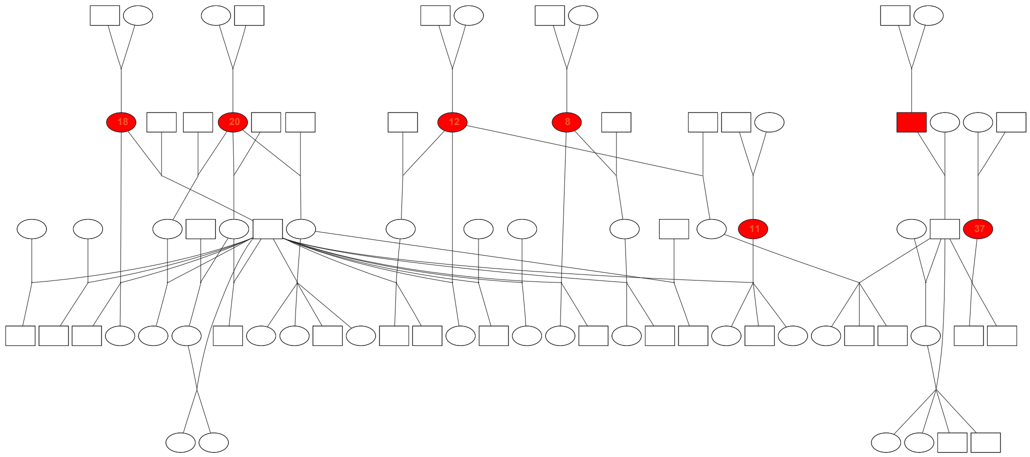

Supplementary Fig 9. Pedigree of wisent from the Białowieża Forest (Poland), from which seven genotyped individuals (in red) were included in the present study.

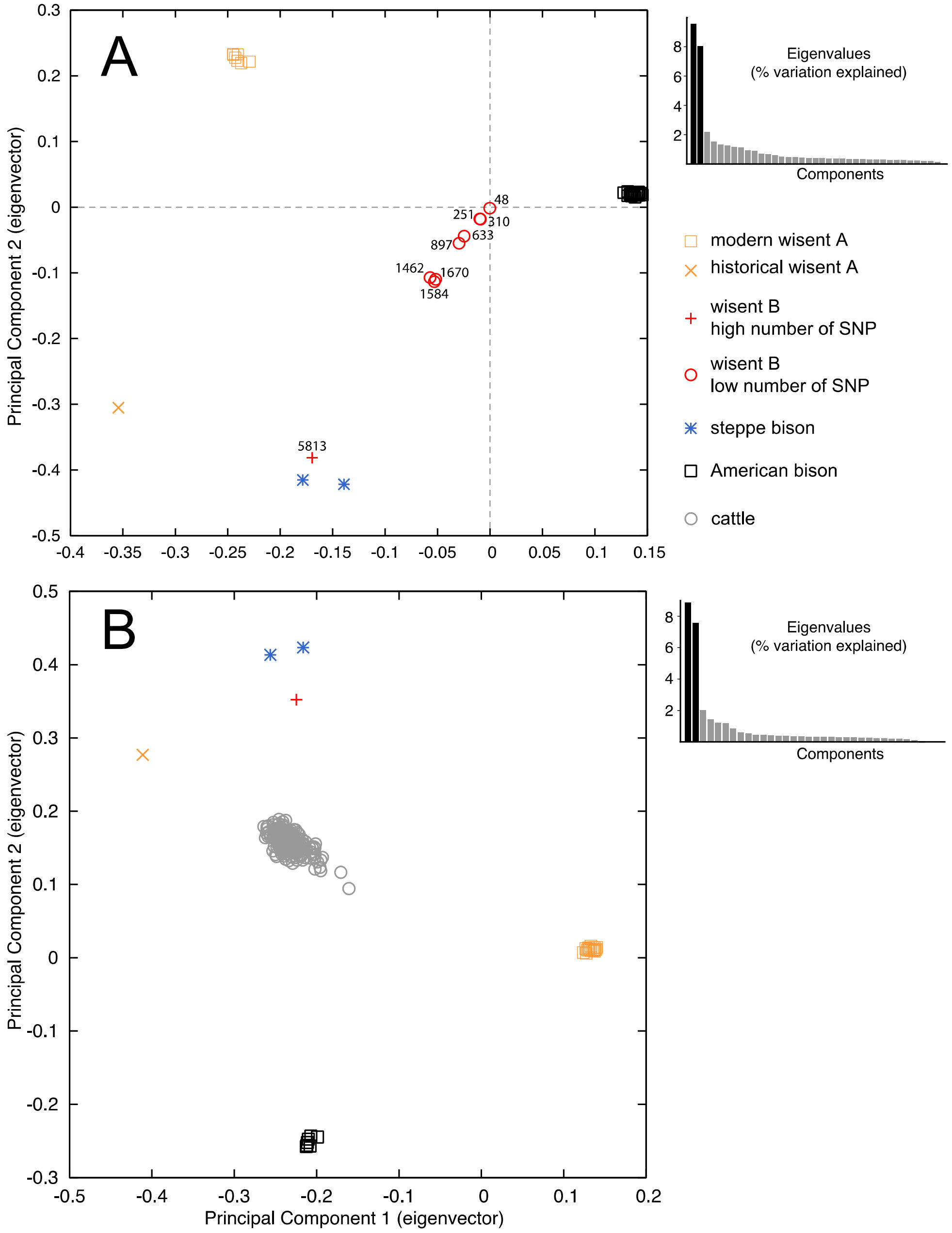

Supplementary Fig 10: A) Principal Component Analysis for nine CladeX individuals (including sample A006), one historical wisent, one ancient wisent, two steppe bison, seven modern wisent and 20 American bison. The numbers on the plot report the number of loci called for the individuals clustering towards zero coordinates (from Supplementary Table 2). Eigenvector 1 explains 9.58% of the variation, while eigenvector 2 explains 7.96% of the variation. B) Same Principal Component Analysis as Figure 3C with cattle individuals from Decker et al. (2009) projected onto original components.

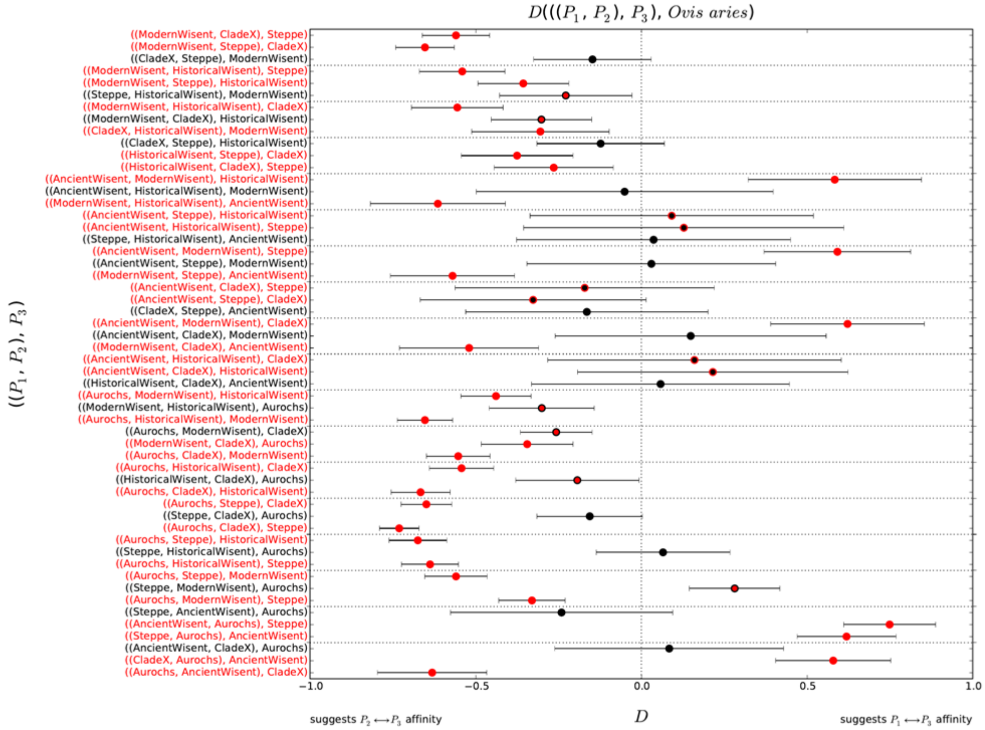

Supplementary Fig 11: Topology testing using D statistics, with sheep as outgroup. The topology being tested is shown on the vertical axis, with the most parsimonious of three possible topologies written in black. Data points that are significantly different (more than three standard errors) from zero are shown in red. The data point representing the topology closest to zero, amongst a set of three possible topologies, is shown with a black outline. Error bars are three standard errors either side of the data point, where the standard error was calculated using a block jackknife.

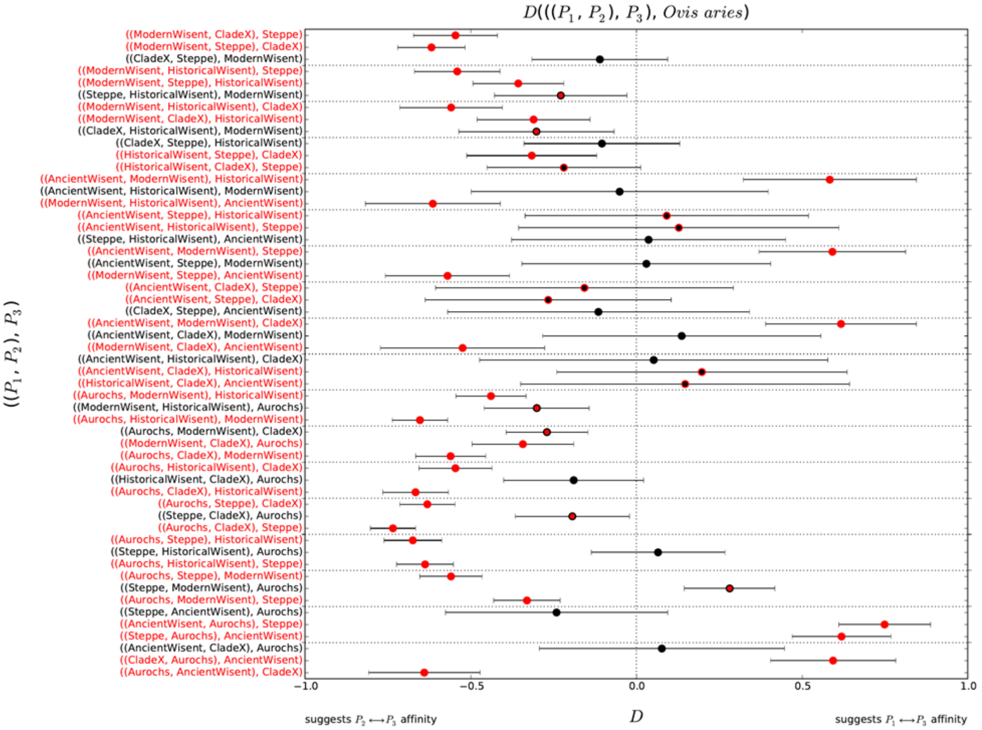

Supplementary Fig 12: Topology testing using D statistics, with sheep as outgroup. As in Supplementary Figure 11, except that sample A006 has been omitted from the CladeX group.

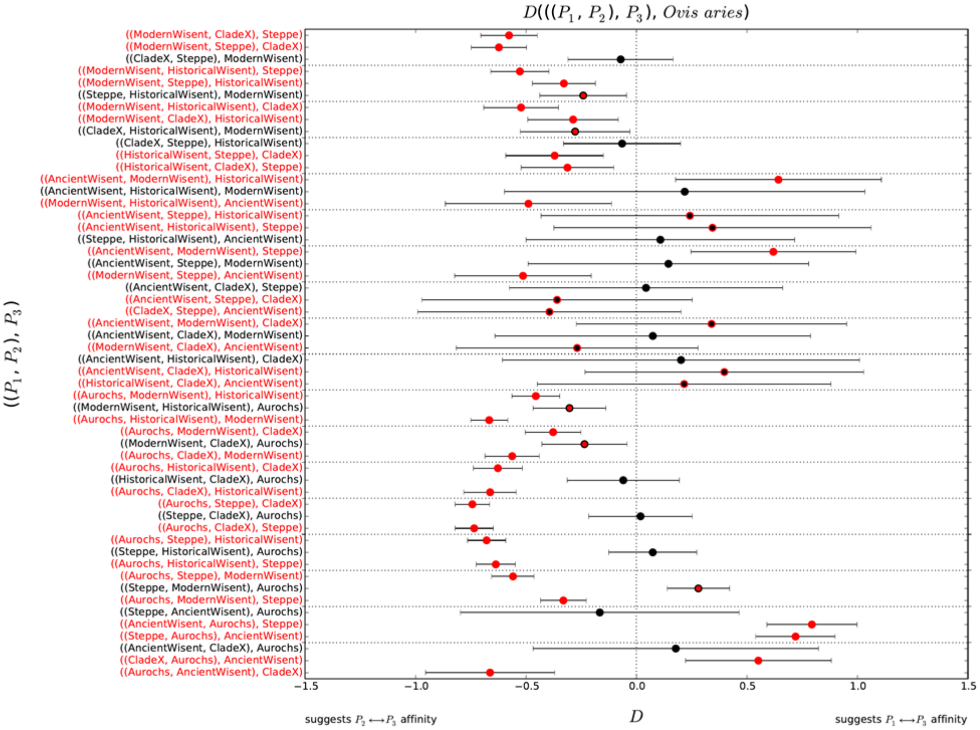

Supplementary Fig 13: Topology testing using D statistics, with sheep as outgroup. As in Supplementary Figure 11, except that genotypes called from read depths <2 have been omitted for extinct individuals.

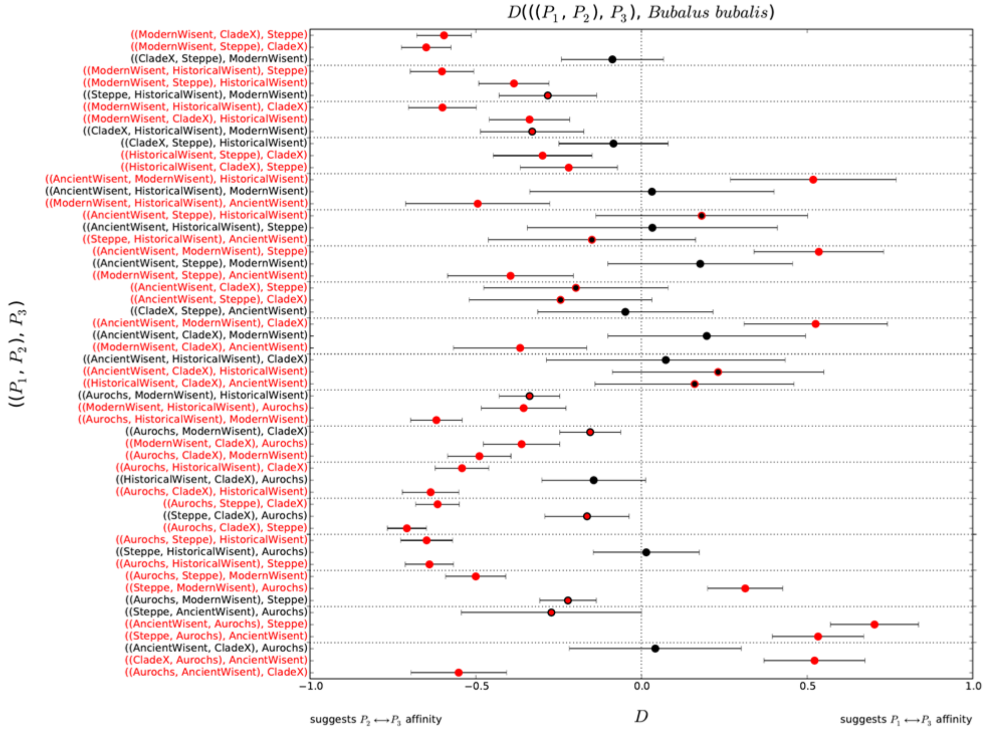

Supplementary Fig 14: Topology testing using D statistics, with Asian water buffalo as outgroup. As in Supplementary Figure 11, except the outgroup has been changed.

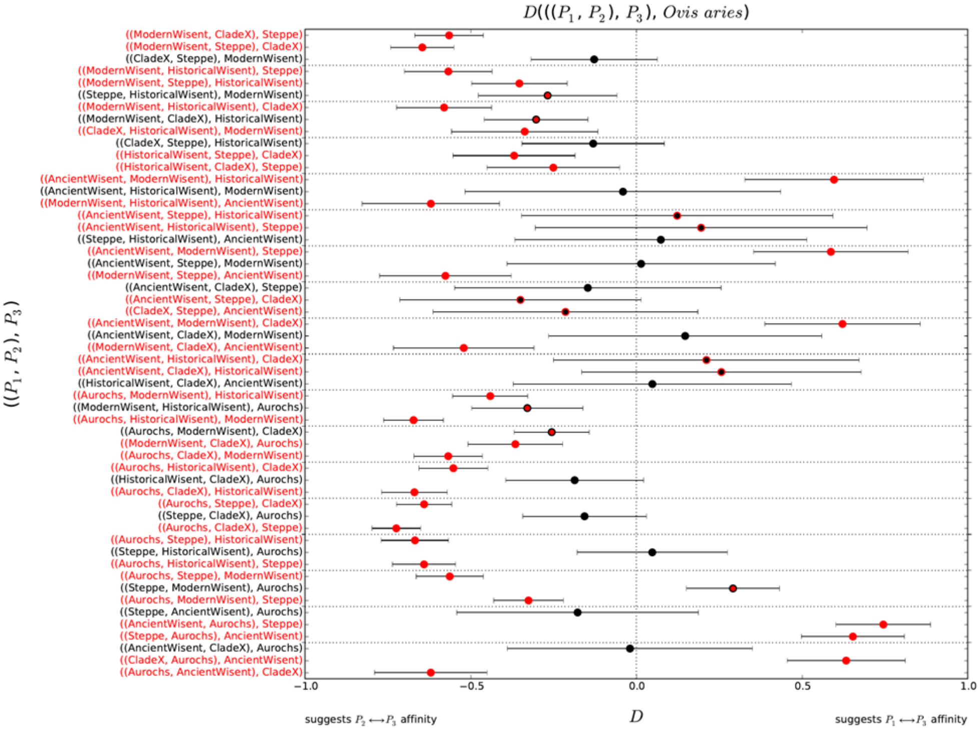

Supplementary Fig 15: Topology testing using D statistics, with sheep as outgroup. As in Supplementary Figure 11, except in extinct individuals, alleles have been randomly sampled from sites called as heterozygotes to simulate haploid sampling.

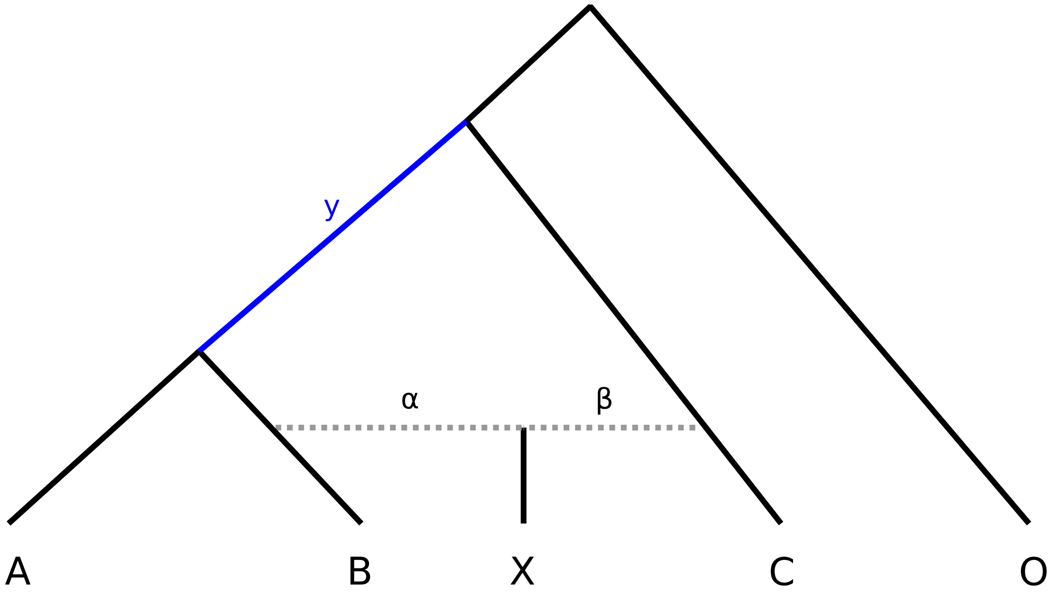

Supplementary Fig 16: An admixture graph showing the ancestry of X, where α is the proportion of ancestry from B and β=1-α is the proportion of ancestry from C.

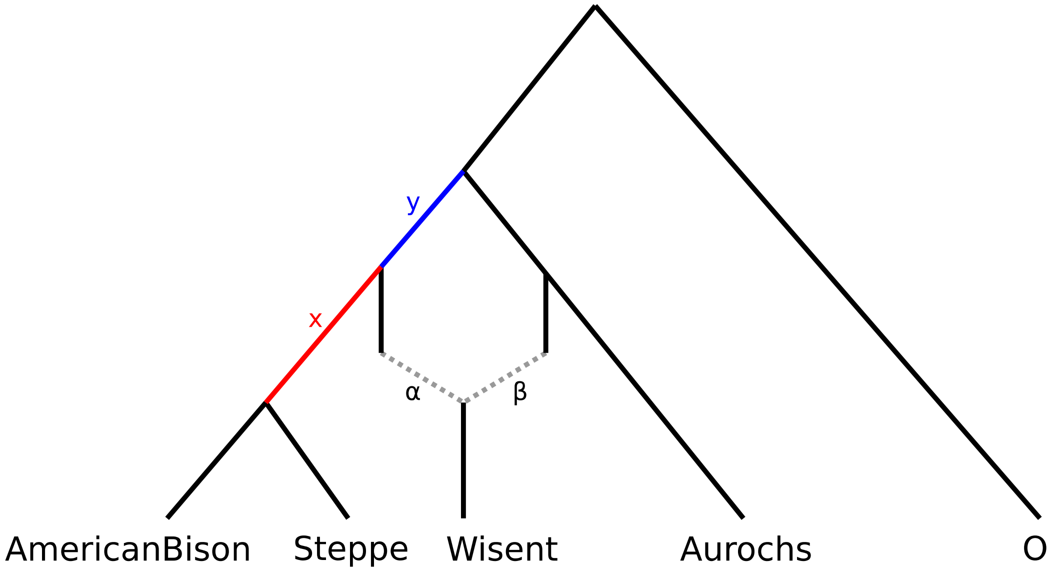

Supplementary Fig 17: An admixture graph showing the ancestry of the wisent, where α is the proportion of ancestry from steppe and β=1-α is the proportion of ancestry from aurochs.

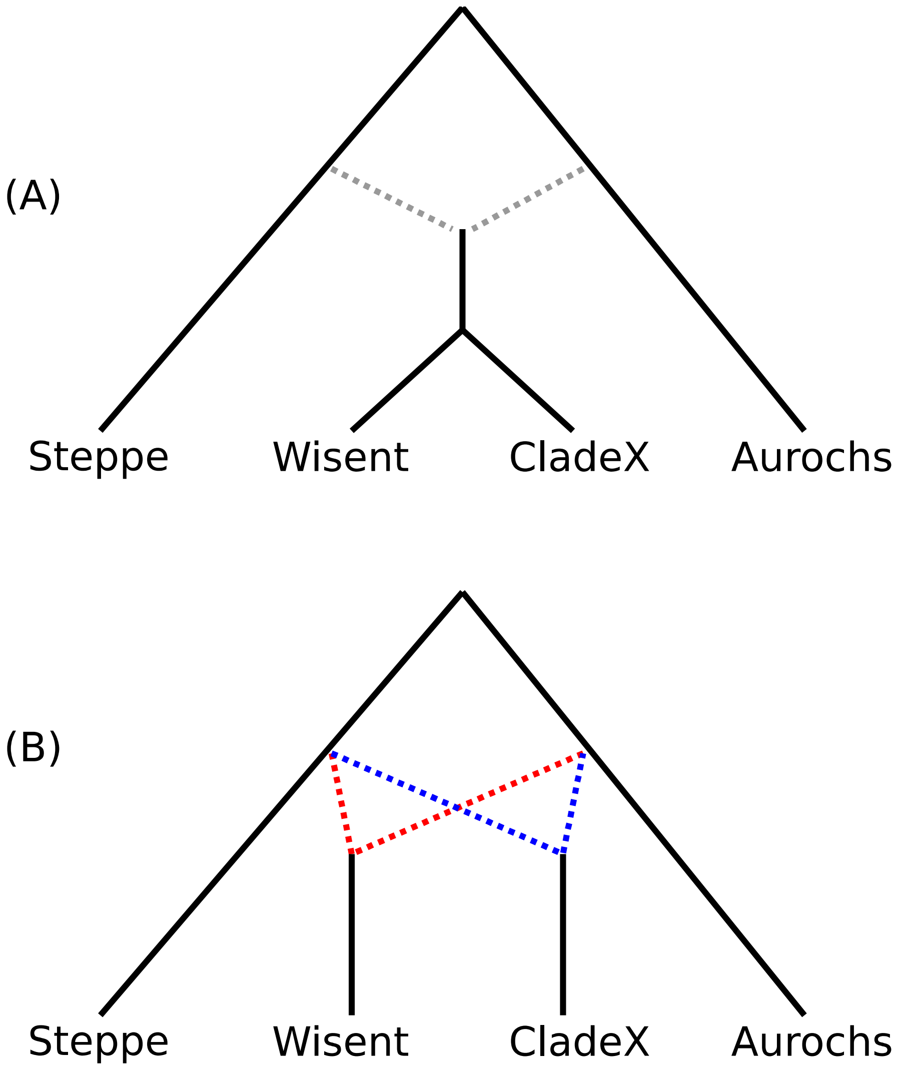

Supplementary Fig 18: Admixture graphs representing (A) a single hybridisation event prior to the divergence of the wisent, and (B) two independent hybridisation events leading to a wisent clade and a CladeX.

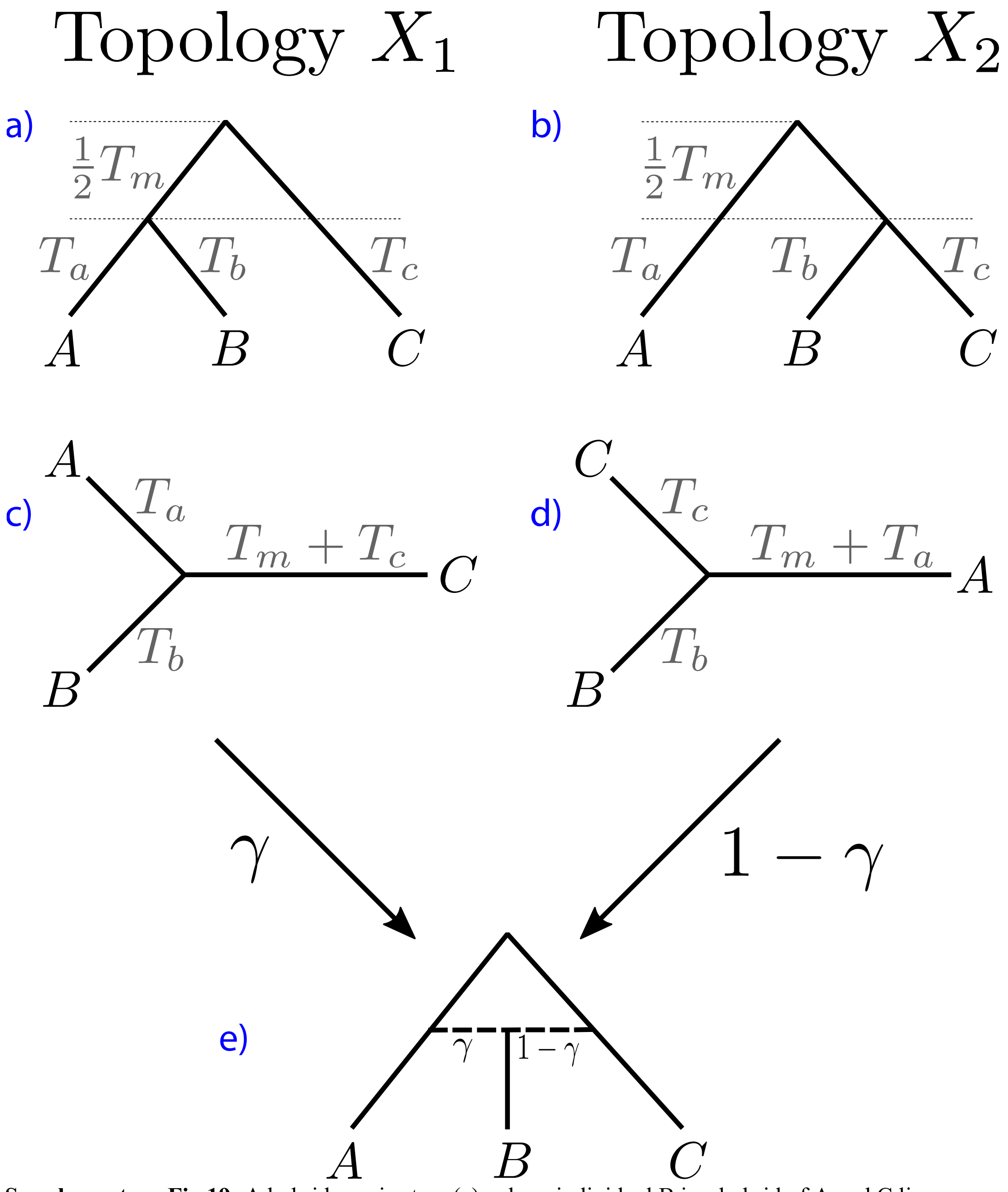

Supplementary Fig 19: A hybrid species tree (e), where individual B is a hybrid of A and C lineages, has two contributing species trees, (a) topology X_1, and (b) topology X_2, with proportion γ from topology X_1 and proportion $1-\gamma$ from topology X_2. The unrooted gene trees are shown for (c) topology X_1, and (d) topology X_2. Branch lengths T_a, T_b, T_c and $T_m$have units $2N_e\mu$ generations.

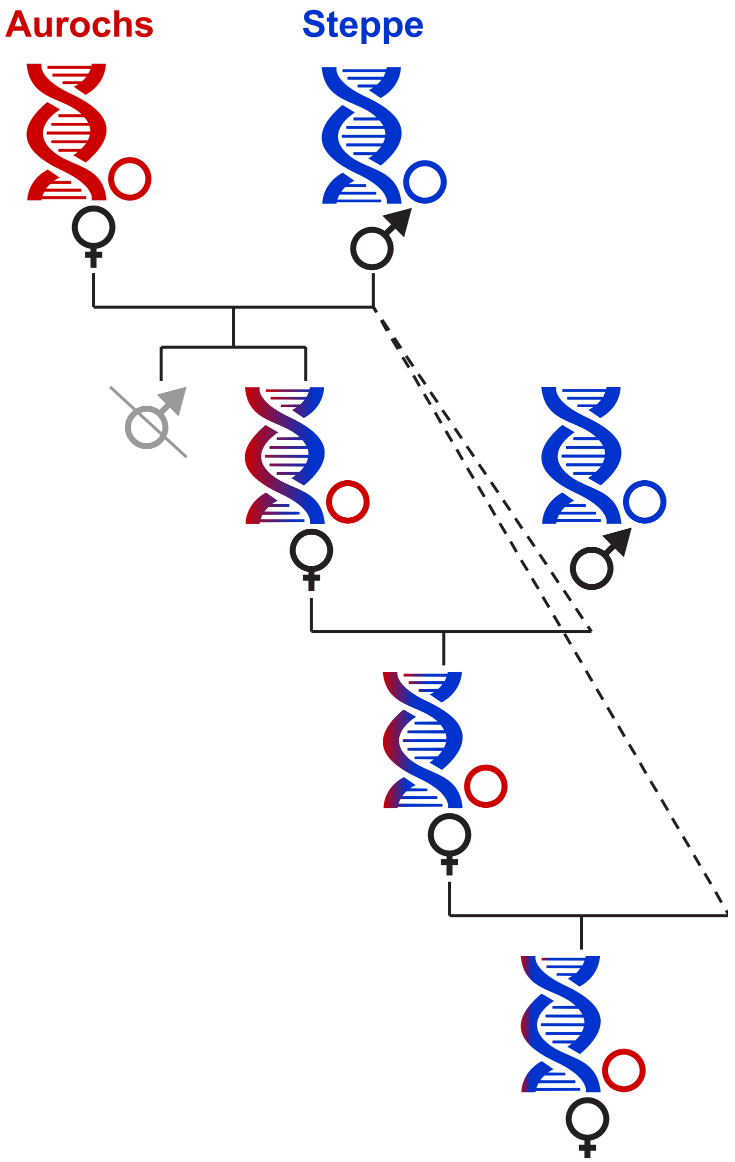

Supplementary Fig 20. Schematic representation of asymmetrical hybridisation between female aurochs and male steppe bison, and its genetic imprint on both nuclear and mitochondrial genomes after a few generations. The coloured double helix represents the nuclear genome, while the circles represent the strictly maternally inherited mitochondrial genome.

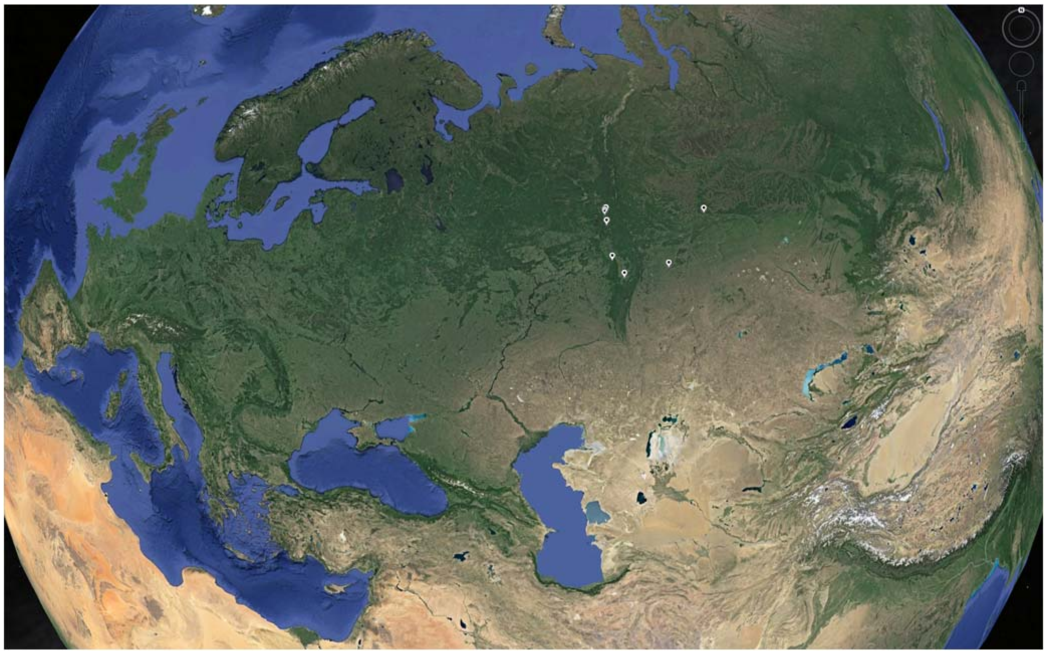

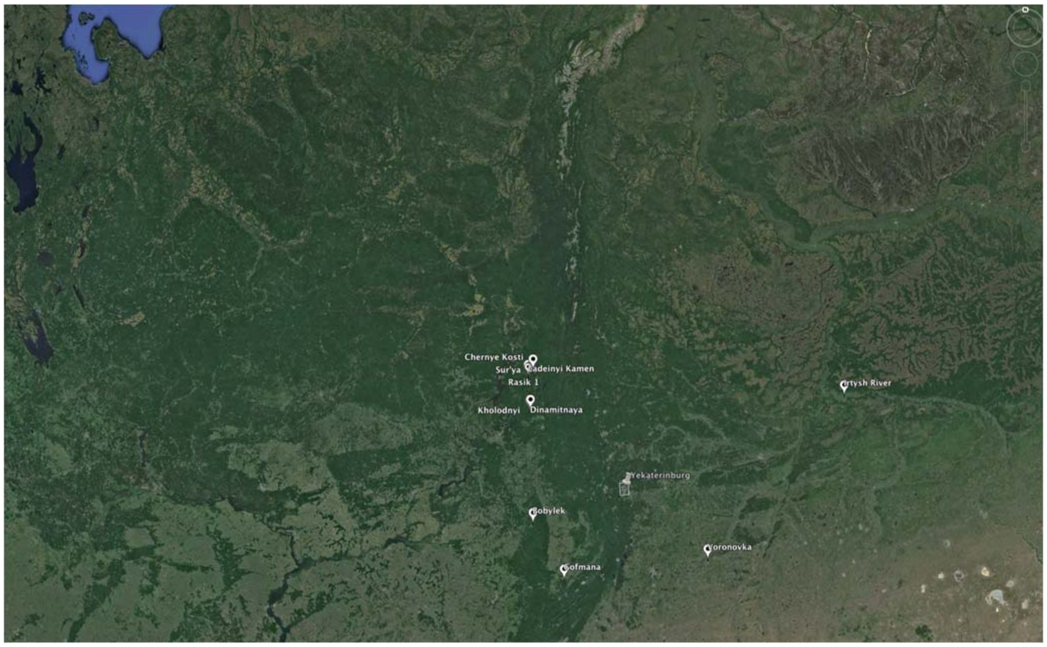

Supplementary Fig 21. Location of all cave sites from which bison samples have been genotyped in the Ural region.

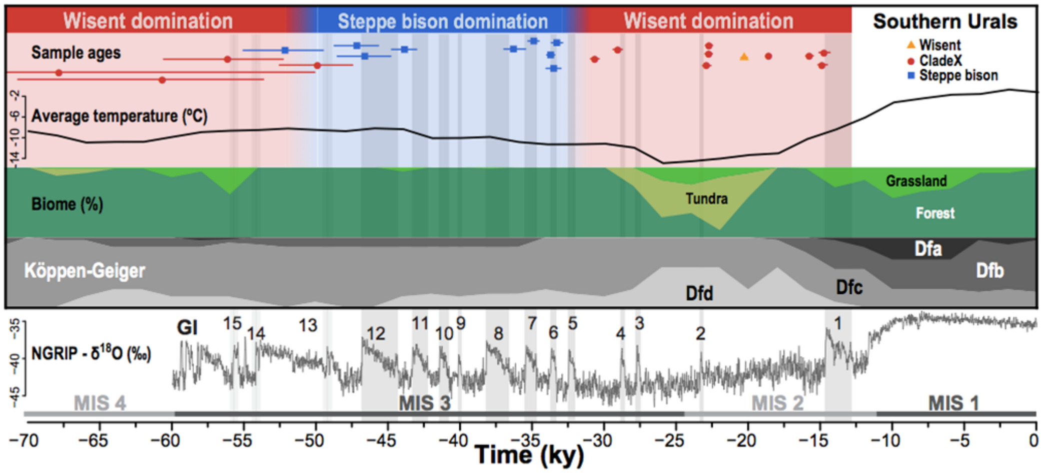

Supplementary Fig 22. Chronology of the Urals samples showing a series of replacement patterns that correlate with climate events. Individual calibrated AMS dates are plotted on top of the NGRIP δO^{18} record [1]. Greenland Interstadials (GI) are numbered in black, and Marine Isotope Stages (MIS) in grey. Inferred average temperature, biome reconstruction and proportion of the area for different Koppen climate classes are shown for the exact region where bison were sampled in southern Urals (Koppen classes: D for 'snow', f for 'fully humid', then a=hot summer; b=warm summer; c=cool summer; d=extremely continental). The most recent population replacement between wisent and steppe bison occurs around 32-33 ky, when major environmental transitions are also observed: 1) Globally, as shown on the NGRIP record with the last major interglacial event (GI 5) before a long period of cold climate; but also 2) Locally, as shown on both the average temperature and biome reconstructions. In this situation, wisent are associated with a cooler climate and the presence of tundra-like vegetation. Although dating resolution is degrading for deeper time, a similar shift is apparent around 50-52 kya. Steppe bison occupied this environment in MIS 3, but have not been detected after this stage and indeed were in a severe population decline by GI 1[2].

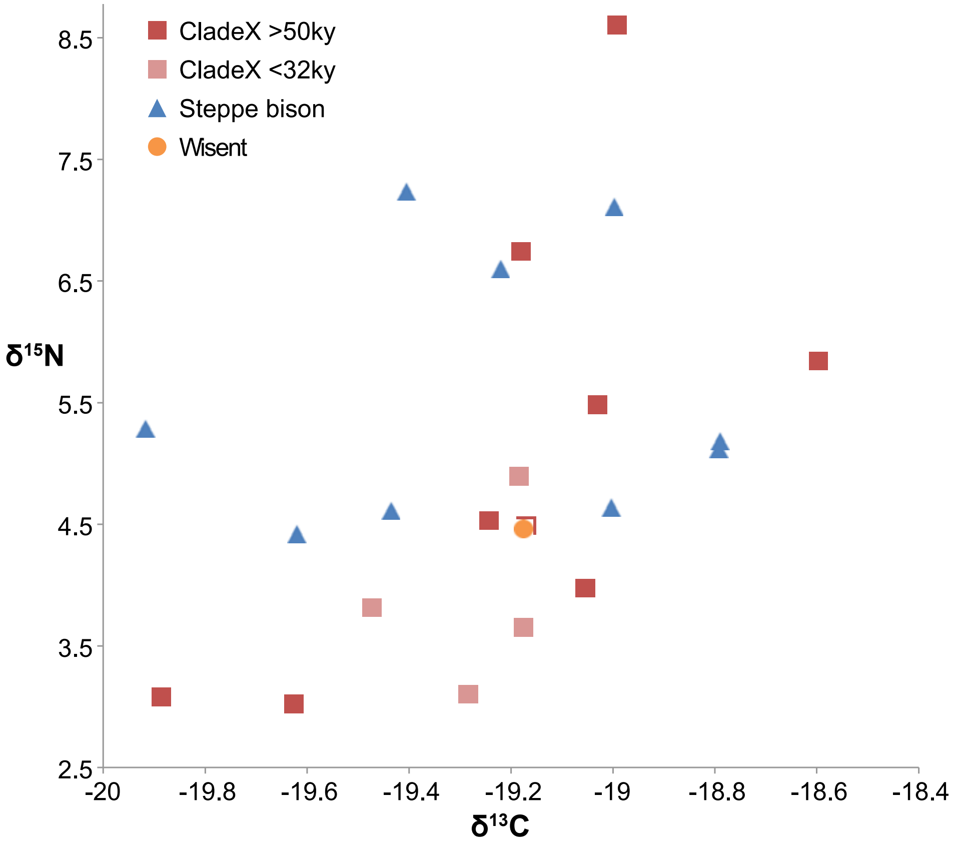

Supplementary Fig 23. Stable δ13C and δ15N isotope values for all genotyped bison sampled from the Ural region.

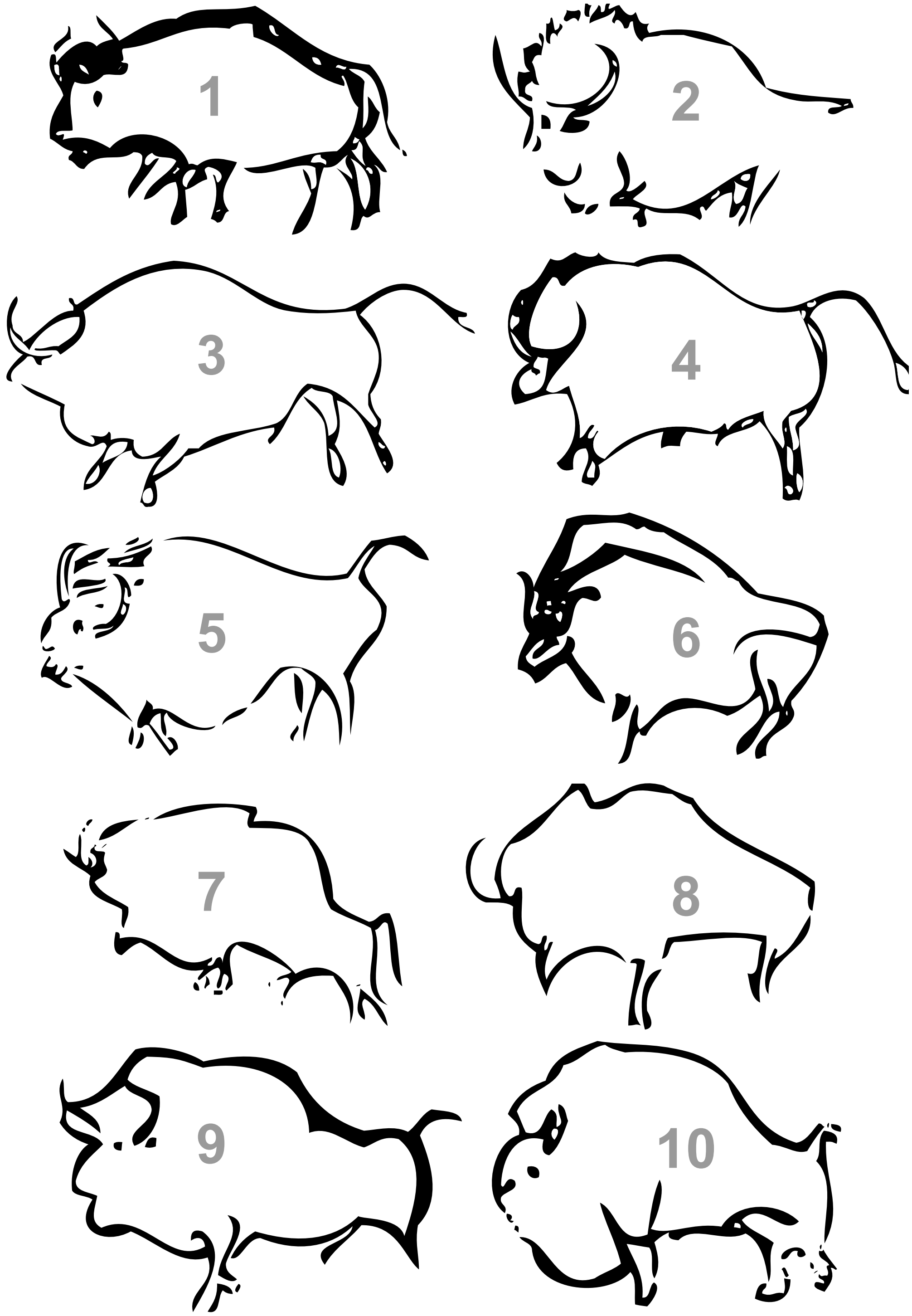

Supplementary Fig 24. Steppe-like morphologies. In European Palaeolithic art, some bison depictions show morphological traits and anatomical details compatible with the morphology of steppe bison (or American bison ancestry). Dates are given as indication based on archaeological occupation determined for each site, or, in the absence of such dating, based on stylistic comparison with other depictions:

1. Grotte Chauvet-Pont d'Arc (Ardèche, France). Blurred black charcoal drawing. Aurignacian period (~35,100 ± 175 calBP. (from C. Fritz and G. Tosello)

2. Grotte de Lascaux (Dordogne, France). Carving. Solutrean (~22,200 ± 380 calBP) or early Magdalenian period (between ~19,300 ± 561 and ~20,597 ± 375 calBP). (adapted from A. Glory[3])

3. Grotte de Lascaux (Dordogne, France). Carving. Solutrean (~22,200 ± 380 calBP) or early Magdalenian period (between ~19,300 ± 561 and ~20,597 ± 375 calBP). (adapted from A. Glory[3])

4. Grotte de Lascaux (Dordogne, France). Carving. Solutrean (~22,200 ± 380 calBP) or early Magdalenian period (between ~19,300 ± 561 and ~20,597 ± 375 calBP). (adapted from A. Glory[3])

5. Grotte du Gabillou (Dordogne, France). Carving. Early Magdalenian period (~20,597 ± 375 calBP). (adapted from J. Gaussen)

6. Grotte des Trois Frères (Ariège, France). Carving. Gravettian period (dating estimated based on stylistic analysis). (adapted from H. Breuil[4])

7. Grotte du Pech Merle (Lot, France). Painting (manganese). Gravettian period (~29,447 ± 443 calBP). (adapted from M. Lorblanchet[5])

8. Grotte du Pech Merle (Lot, France). Painting (manganese). Gravettian period (~29,447 ± 443 calBP). (adapted from M. Lorblanchet[5])

9. Grotte de La Pasiega (Cantabria, Spain). Black and red painting. Gravettian or Solutrean period (dating estimated based on stylistic analysis). (adapted from H. Breuil[4])

10. Abri du Roc de Sers (Charente, France). Carving on limestone. Solutrean period (< 20,442 ± 409 calBP). (adapted from L. Henri-Martin)

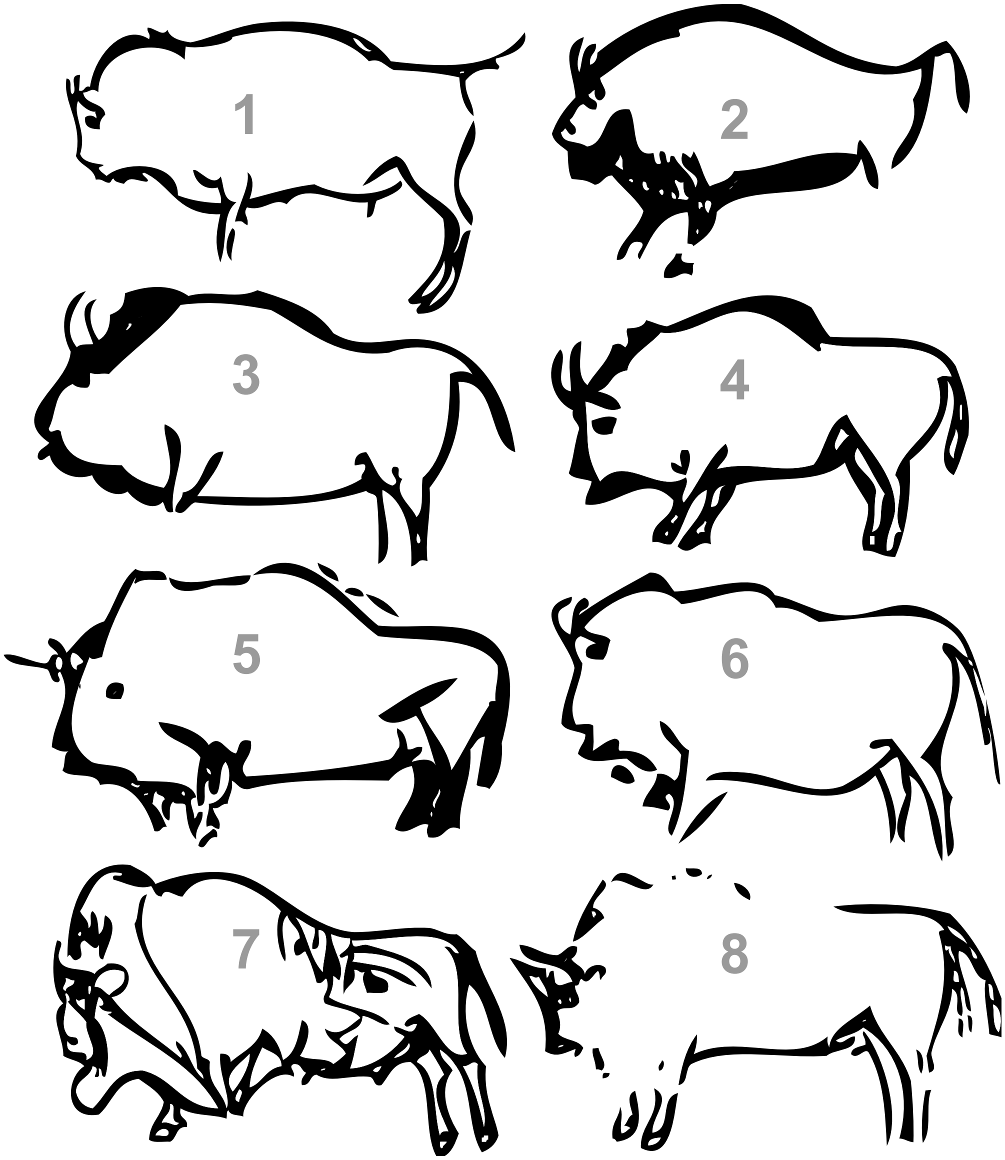

Supplementary Fig 25. Wisent-like morphologies. In European Palaeolithic art, some bison depictions show morphological traits and anatomical details compatible with identification of wisent ancestry. Dates are given as indication based on archaeological occupation determined for each site, or, in the absence of such dating, based on stylistic comparison with other depictions:

1. Grotte de Pergouset (Ardèche, France). Carving. Magdalenian period (dating estimated based on stylistic analysis). (adapted from M. Lorblanchet[5])

2. Grotte du Portel (Ariège, France). Painting. Magdalenian period (~14,250 ± 295 calBP). (adapted from H. Breuil[4])

3. Grotte de Niaux (Ariège, France). Painting. Magdalenian period (~17,000 ± 260 calBP). (adapted from H. Breuil[4])

4. Grotte de Niaux (Ariège, France). Painting. Magdalenian period (~17,000 ± 260 calBP). (adapted from H. Breuil[4])

5. Grotte de Fontanet (Ariège, France). Carving. Magdalenian period (between ~14250 ± 295 calBP and ~16,600 ± 1000 calBP). (adapted from A. Glory[3])

6. Grotte de Rouffignac (Dordogne, France). Painting. Magdalenian period (dating estimated based on stylistic analysis). (adapted from C. Barrière[6])

7. Grotte des Combarelles (Dordogne, France). Carving. Magdalenian period (between ~17,000 and ~14,300 calBP). (adapted from H. Breuil[4])

8. Grotte de Marsoulas (Haute-Garonne, France). Carving. Magdalenian period (dating estimated based on stylistic analysis). (from C. Fritz et G. Tosello)

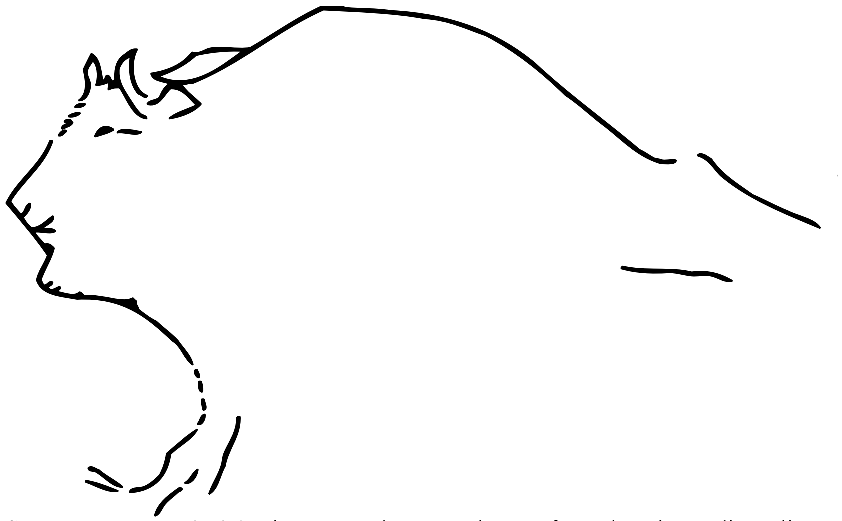

Supplementary Fig 26. Bison carved on round stone from the Riparo di Tagliente site in Italy

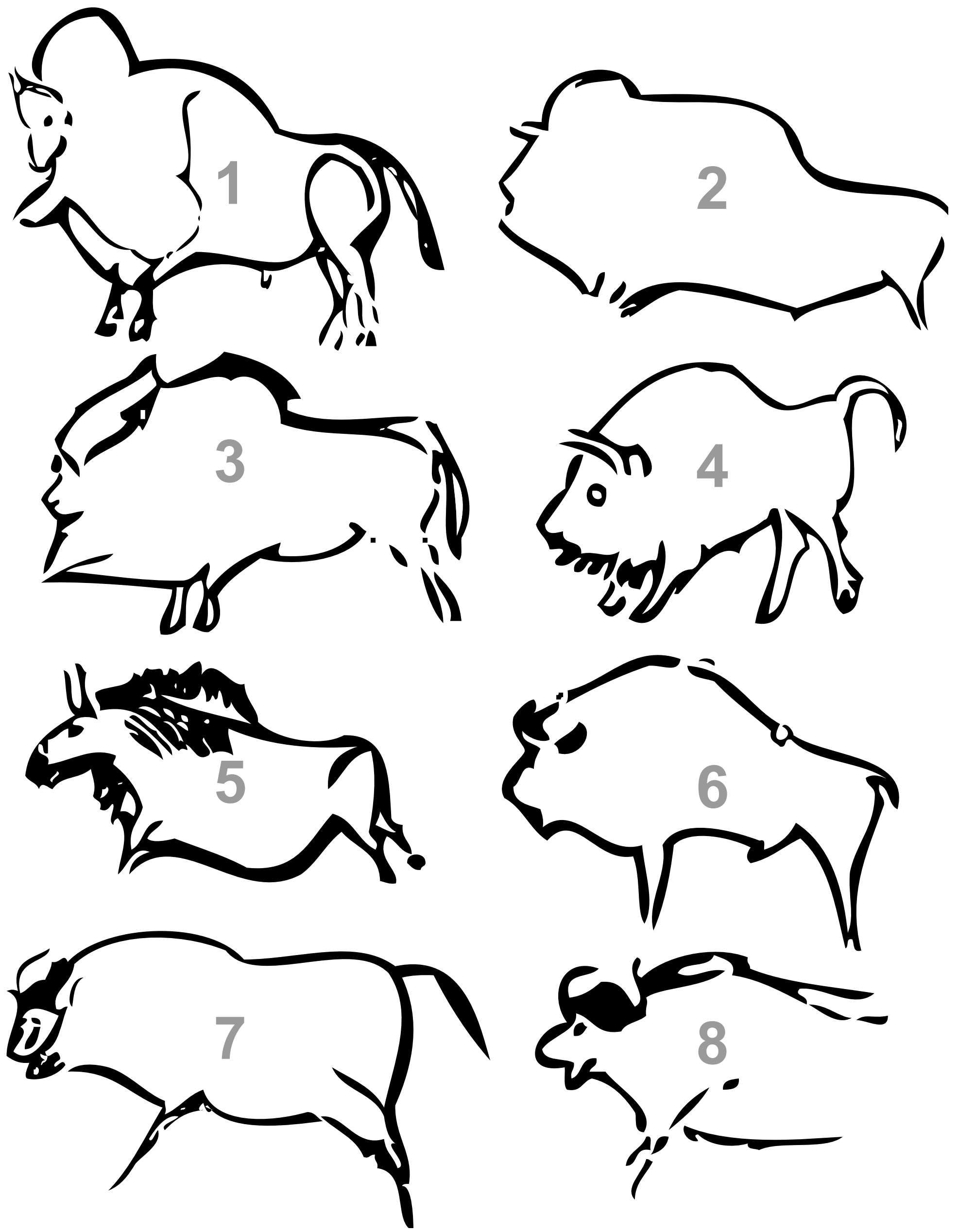

Supplementary Fig 27. Undetermined morphologies. In European Palaeolithic art, some bison depictions show morphological traits and anatomical details that could be compatible with either bison form. These pictures illustrate the limits of cave art analyses for morphological assessment of bison forms, due to varying graphical conventions between cultures. Dates are given as indication based on archaeological occupation determined for each site, or, in the absence of such dating, based on stylistic comparison with other depictions:

1 Grotte de Font-de-Gaume (Dordogne, France). Black and red painting, and carving. Magdalenian period (dating estimated based on stylistic analysis). (adapted from H. Breuil[4])

2 Grotte de Niaux (Ariège, France). Painting. Magdalenian period (~17,000 ± 260 calBP). (adapted from H. Breuil[4])

3 Grotte des Trois Frères (Ariège, France). Carving. Magdalenian period (dating estimated based on stylistic analysis). (adapted from H. Breuil[4])

4 Grotte des Trois Frères (Ariège, France). Carving. Magdalenian period (dating estimated based on stylistic analysis). (adapted from H. Breuil[4])

5 Grotte des Trois Frères (Ariège, France). Carving. Gravettian period (dating estimated based on stylistic analysis). (adapted from H. Breuil[4])

6 Grotte de La Grèze (Dordogne, France). Carving. Gravettian period (dating estimated based on stylistic analysis) (adapted from N. Aujoulat)

7 Grotte Chauvet-Pont d'Arc (Ardèche, France). Blured black charcoal drawing. Aurignacian period (~35100 ± 175 calBP). (from C. Fritz-G. Tosello)

8 Grotte Chauvet-Pont d'Arc (Ardèche, France). Blured black charcoal drawing. Aurignacian period (~35100 ± 175 calBP). (from C. Fritz-G. Tosello)

Supplementary Tables

Supplementary Table 1. Primers and adapters used in this study

	Primer	Primer Sequence (5' - 3')	Length [a]
Set_ A1	BovCR-16351F	CAACCCCCAAAGCTGAAG	~96bp
	BovCR-16457R	TGGTTRGGGTACAAAGTCTGTG	
Set_ B1	BovCR-16420F	CCATAAATGCAAAGAGCCTCAYCAG	~172bp
	BovCR-16642R	TGCATGGGGCATATAATTTAATGTA	
Set_ A2	BovCR-16507F	AATGCATTACCCAAACRGGG	~184bp
	BovCR-16755R	ATTAAGCTCGTGATCTARTGG	
Set_ B2	BovCR-16633F[b]	GCCCCATGCATATAAGCAAG	~132bp
	BovCR-16810R[b]	GCCTAGCGGGTTGCTGGTTTCACGC	
Set_ A3	BovCR-16765F[b]	GAGCTTAAYTACCATGCCG	~125bp
	BovCR-16998R	CGAGATGTCTTATTTAAGAGGAAAGAATGG	
Set_ B3	BovCR-16960F	CATCTGGTTCTTTCTTCAGGGCC	~110bp
	BovCR-80R[b]	CAAGCATCCCCCAAAATAAA	
Frag1	BovCR_16738MF[c,d]	***CACGACGTTGTAAAACGAC***ATYGTACATAGYACATTATGTCAA	~67bp
	BovCR_16810TR[c,d]	***TACGACTCACTATAGGGCGA***GCCTAGCGGGTTGCTGGTTTCACGC	
Frag2	Mamm_12SE[d]	CTATAATCGATAAACCCCGATA	~96bp
	Mamm_12SH[d]	GCTACACCTTGACCTAAC	
	GAII_Indexing_x	CAAGCAGAAGACGGCATACGAGATNNNNNNNGAGTGACTGGAGTTCAGACGTGT	n/a
	IS4_indPCR.P5[e]	AATGATACGGCGACCACCGAGATCTACACTCTTTCCCTACACGACGCTCTT	n/a
	IS7_short_amp.P5[e]	ACACTCTTTCCCTACACGAC	n/a
	IS8_short_amp.P7[e]	GTGACTGGAGTTCAGACGTGT	n/a
	P5_short_RNAblock	ACACUCUUUCCCUACACGAC	n/a
	P7_short_RNAblock	GUGACUGGAGUUCAGACGUGU	n/a
	Bison_mt1_forward [f]	ACCGCGGTCATACGATTAAC	
	Bison_mt1_reverse [f]	AATTGCGAAGTGGATTTTGG	
	Bison_mt2_forward [f]	ATGAGCCAAAATCCACTTCG	
	Bison_mt2_reverse [f]	TGTATTTGCGTCTGCTCGTC	
	Bison_mt3_forward [f]	CGAATCCACAGCCGAACTAT	
	Bison_mt3_reverse [f]	TATAAAGCACCGCCAAGTCC	

(a): Primers are excluded from the length of PCR amplicon.

(b):[2].

(c): M13 (CAC GAC GTT GTA AAA CGA C) and T7 (TAC GAC TCA CTA TAG GGC GA) sequences were used as tags for primers BovCR_16738F and BovCR_16810R, respectively. This was done to obtain good quality Sanger sequences from short amplicons.

(d): One-step simplex PCRs.

(e): (Meyer and Kircher, "Illumina Sequencing Library Preparation for Highly Multiplexed Target Capture and Sequencing.")

(f): Primer pairs for use to generate DNA baits for mitochondrial DNA capture.

Supplementary Table 2. Summary of nuclear alleles detected at bovine SNP loci: NGS results and locus counts for ancient samples; locus counts for modern samples

Sample ID	Method	Mapping results for the 9908 SNP positions						Number of SNP called out of the 9908 targeted for each ancient individuals							
								Coverage depth >=1				Coverage depth >=2			
		Retained_reads	hits_raw	hits_unique	hits_raw_frac	hits_clonality	Mean coverage	Total	REF/REF	REF/ALT	ALT/ALT	Total	REF/REF	REF/ALT	ALT/ALT
**A15526**		7045	1821	99	0.26	0.95	0.01	**49**	49	0	0	**1**	1	0	0
**A017**		1280556	3893	1289	0.00	0.67	0.13	**630**	591	0	39	**88**	49	0	39
**A018**		967346	3116	538	0.00	0.83	0.05	**253**	241	0	12	**28**	16	0	12
**A001**		656008	392937	3486	0.60	0.99	0.35	**1484**	1268	2	214	**523**	307	2	214
**A003**		1706985	12957	3423	0.01	0.74	0.35	**1569**	1363	5	201	**470**	264	5	201
**A004**	10k capture	240370	132883	645	0.55	1.00	0.07	**315**	287	0	28	**64**	36	0	28
**A005**		1736500	25788	3519	0.01	0.86	0.35	**1643**	1438	7	198	**464**	259	7	198
**A006**		10413909	99392	22312	0.01	0.78	2.25	**5690**	3468	104	2118	**4755**	2533	104	2118
**A007**		3583539	23832	2841	0.01	0.88	0.29	**1307**	1084	1	222	**509**	286	1	222
**A15654**		1700840	1227601	220913	0.72	0.82	22.28	**8738**	4532	230	3976	**8488**	4282	230	3976
**A4093**		9400283	62631	4478	0.01	0.93	0.45	**1946**	1480	2	464	**1031**	565	2	464
**A3133**	Shotgun / 10k	299829433	9812523	465082	0.03	0.95	46.87	**8898**	4579	321	3998	**8680**	4361	321	3998
**A875**	and 40k capture	3908972	291640	234493	0.07	0.20	23.65	**8433**	4341	342	3750	**8144**	4052	342	3750
**CPC98_Aurochs**	From published genome							**8882**	4770	1808	2304	**8810**	4698	1808	2304

Supplementary Table 3. Summary statistics for NGS of whole mitochondrial genomes

Sample ID	Retained_reads	hits_raw	hits_unique	hits_raw_frac	hits_clonality	AVG_Depth	STD_Depth	AVE_Length	STD_Length	5pC>T	3pG>A	Library repair
A001	4822143	1618364	86944	0.34	0.95	432.09	224.83	80.82	37.60	0.03	0.02	
A004	5150804	2314449	220697	0.45	0.90	1152.17	541.88	84.88	36.11	0.02	0.02	
A018	3790161	1021750	24699	0.27	0.98	130.53	60.04	85.32	34.05	0.03	0.03	USER
A4089	8618722	5380606	44044	0.62	0.99	237.83	155.46	87.18	33.56	0.02	0.02	
A3133	66864927	1958	1949	0.00	0.00	11.41	6.77	93.92	29.66	0.00	0.01	
A003	985033	371605	64372	0.38	0.83	334.44	112.68	84.31	34.07	0.08	0.07	
A005	521428	262622	39121	0.50	0.85	196.95	65.76	81.59	30.96	0.05	0.09	
A006	456078	120668	44541	0.26	0.63	208.39	93.86	75.86	25.87	0.13	0.17	
A007	431113	175432	43269	0.41	0.75	192.35	85.93	71.74	24.13	0.11	0.08	
A4093	212315	106221	16923	0.50	0.84	73.23	31.26	70.48	24.60	0.07	0.09	Partial UDG
A15637	469884	4401	2621	0.01	0.40	8.85	7.22	50.41	12.17	0.41	0.35	
A15654	294965	29628	28329	0.10	0.04	170.48	89.68	98.23	34.91	0.05	0.02	
A15668	230709	3603	2842	0.02	0.21	11.07	7.80	59.61	15.06	0.07	0.06	
LE237	507023	4271	2677	0.01	0.37	9.84	5.70	58.98	23.99	0.55	0.51	
LE242	6912671	48793	35418	0.01	0.27	120.46	67.86	55.09	18.68	0.61	0.60	None
LE257	4156307	184236	28788	0.04	0.84	94.38	38.34	53.17	20.00	0.52	0.50	

Supplementary Table 4. List of published mitochondrial control region sequences used for phylogenetic analysis. The Urals steppe bison are highlighted in red.

**American bison**	Bison_priscus_BS146_NS_11810_50	Bison_priscus_BS397_NS_32370_470	Bos_indicus_AY378135_0_0
Bison_bison_AF083357_H1_0_0	Bison_priscus_BS147_NS_28120_290	Bison_priscus_BS398_NS_27400_260	Bos_indicus_DQ887765_0_0
Bison_bison_AF083358_H2_0_0	Bison_priscus_BS148_NS_6400_50	Bison_priscus_BS400_NS_46100_2600	Bos_indicus_EF417971_0_0
Bison_bison_AF083359_H3_0_0	Bison_priscus_BS149_NS_46100_2200	Bison_priscus_BS405_SI_23040_120	Bos_indicus_EF417974_0_0
Bison_bison_AF083360_H4_0_0	Bison_priscus_BS150_NS_10510_50	Bison_priscus_BS407_NWT_55500_3100	Bos_indicus_EF417976_0_0
Bison_bison_AF083361_H5_0_0	Bison_priscus_BS151_NS_21530_130	Bison_priscus_BS412_Y_30500_250	Bos_indicus_EF417977_0_0
Bison_bison_AF083362_H6_0_0	Bison_priscus_BS161_NS_21040_120	Bison_priscus_BS414_BlR_4495_60	Bos_indicus_EF417979_0_0
Bison_bison_AF083363_H7_0_0	Bison_priscus_BS163_LC_13240_75	Bison_priscus_BS415_D_30810_975	Bos_indicus_EF417981_0_0
Bison_bison_AF083364_H8_0_0	Bison_priscus_BS164_LC_19540_120	Bison_priscus_BS418_China_26560_670	Bos_indicus_EF417983_0_0
Bison_bison_BS100_29_5	Bison_priscus_BS165_LC_26460_160	Bison_priscus_BS438_AB_53800_2200	Bos_indicus_EF417985_0_0
Bison_bison_BS102_22_5	Bison_priscus_BS170_YT_13040_70	Bison_priscus_BS440_AB_60400_2900	Bos_indicus_EF524120_0_0
Bison_bison_BS129_0_2000	Bison_priscus_BS172_LC_12525_70	Bison_priscus_BS443_AB_34050_450	Bos_indicus_EF524125_0_0
Bison_bison_BS162_AK_170_30	Bison_priscus_BS176_LC_12380_60	Bison_priscus_BS459_China_47700_1000	Bos_indicus_EF524126_0_0
Bison_bison_BS173_NTC_3220_45	Bison_priscus_BS178_LC_17960_90	Bison_priscus_BS469_AB_305_24	Bos_indicus_EF524128_0_0
Bison_bison_BS175_ICE_186_30	Bison_priscus_BS192_F_26300_300	Bison_priscus_BS472_F_13235_65	Bos_indicus_EF524130_0_0
Bison_bison_BS177_NTC_3155_36	Bison_priscus_BS193_NS_49600_4000	Bison_priscus_BS473_AB_56300_3100	Bos_indicus_EF524132_0_0
Bison_bison_BS200_AB_145_37	Bison_priscus_BS195_NS_29040_340	Bison_priscus_BS477_D_33710_240	Bos_indicus_EF524135_0_0
Bison_bison_BS342_CHL_10340_40	Bison_priscus_BS196_NS_19420_100	Bison_priscus_BS478_D_34470_200	Bos_indicus_EF524141_0_0
Bison_bison_BS348_CHL_10505_45	Bison_priscus_BS198_Y_2460_40	Bison_priscus_BS490_BlR_2415_25	Bos_indicus_EF524152_0_0
Bison_bison_BS368_0_2000	Bison_priscus_BS201_Y_12960_60	Bison_priscus_BS493_NS_50000_4200	Bos_indicus_EF524156_0_0
Bison_bison_BS417_AB_909_29	Bison_priscus_BS202_AB_10460_65	Bison_priscus_BS494_NS_44800_2200	Bos_indicus_EF524160_0_0
Bison_bison_BS419_AB_7475_45	Bison_priscus_BS206_Sibh_23780_140	Bison_priscus_BS495_NS_29570_340	Bos_indicus_EF524166_0_0
Bison_bison_BS421_AB_8145_45	Bison_priscus_BS211_Sibh_43800_1100	Bison_priscus_BS497_NS_30000_540	Bos_indicus_EF524167_0_0
Bison_bison_BS422_AB_908_31	Bison_priscus_BS216_NS_47000_2900	Bison_priscus_BS498_NS_25980_230	Bos_indicus_EF524170_0_0
Bison_bison_BS423_AB_4660_38	Bison_priscus_BS218_Si_14605_75	Bison_priscus_BS499_NS_31410_420	Bos_indicus_EF524177_0_0
Bison_bison_BS424_AB_202_32	Bison_priscus_BS222_NWT_6110_45	Bison_priscus_BS500_NS_35580_550	Bos_indicus_EF524180_0_0
Bison_bison_BS426_AB_7060_45	Bison_priscus_BS223_Si_53300_1900	Bison_priscus_BS517_BlR_2526_26	Bos_indicus_EF524183_0_0
Bison_bison_BS428_AB_7105_45	Bison_priscus_BS224_AK_13125_75	Bison_priscus_BS564_Si_24570_90	Bos_indicus_EF524185_0_0
Bison_bison_BS429_AB_6775_40	Bison_priscus_BS233_SW_16685_80	Bison_priscus_BS571_SIdy_32910_170	Bos_indicus_L27732_0_0
Bison_bison_BS430_9270_50	Bison_priscus_BS235_BlR_43400_900	Bison_priscus_BS592_Urals_42500_450	Bos_indicus_L27736_0_0
Bison_bison_BS432_AB_7310_45	Bison_priscus_BS236_SW_19420_100	Bison_priscus_BS605_NTC_20380_90	**Aurochs**
Bison_bison_BS433_AB_10450_55	Bison_priscus_BS237_AB_11240_70	Bison_priscus_BS660_Urals_29500_140	Bos_primigenius_DQ915522_ALL1_12030_52
Bison_bison_BS434_AB_809_32	Bison_priscus_BS243_SW_37550_400	Bison_priscus_BS662_SI_20000_0	Bos_primigenius_DQ915523_CAT1_5650_0
Bison_bison_BS439_AB_5845_45	Bison_priscus_BS244_LC_26210_170	Bison_priscus_BS674_Urals_29060_140	Bos_primigenius_DQ915524_CHWF_3905_185
Bison_bison_BS441_AB_1273_32	Bison_priscus_BS248_OCr_12350_70	Bison_priscus_BS708_Urals_47050_750	Bos_primigenius_DQ915537_CPC98_5936_34
Bison_bison_BS444_AB_636_29	Bison_priscus_BS249_F_39200_550	Bison_priscus_BS713_Urals_30970_180	Bos_primigenius_DQ915542_EIL06_5830_29
Bison_bison_BS445_AB_378_30	Bison_priscus_BS253_LC_12665_65	Bison_priscus_IB179_LC_12465_75	Bos_primigenius_DQ915543_EIL14_5830_29
Bison_bison_BS449_6195_45	Bison_priscus_BS254_CHL_10230_55	**European bison**	Bos_primigenius_DQ915554_LJU3_8020_50
Bison_bison_BS454_AB_287_29	Bison_priscus_BS258_F_22120_130	Bison_bonasus_AF083356_0_0	Bos_primigenius_DQ915558_NORF_3370_30
Bison_bison_BS456_AB_125_30	Bison_priscus_BS260_D_30750_290	Bison_bonasus_AY428860_0_0	Bos_primigenius_EF187280_PVL04_3204_56
Bison_bison_BS460_AB_10425_50	Bison_priscus_BS261_LC_12915_70	Bison_bonasus_EF693811_0_0	**Cattle**
Bison_bison_BS464_AB_5205_45	Bison_priscus_BS262_D_29150_500	Bison_bonasus_EU272053_0_0	Bos_taurus_DQ124372_T4_0_0
Bison_bison_BS465_AB_7115_50	Bison_priscus_BS281_BlR_40800_600	Bison_bonasus_EU272054_0_0	Bos_taurus_DQ124375_T4_0_0
Bison_bison_BS466_AB_3298_37	Bison_priscus_BS282_Si_56700_3200	Bison_bonasus_EU272055_0_0	Bos_taurus_DQ124381_T3_0_0
Bison_bison_BS503_BlR_2776_36	Bison_priscus_BS284_Y_13135_65	Bison_bonasus_U12953_0_0	Bos_taurus_DQ124383_T2_0_0
Bison_bison_BS560_AB_2807_28	Bison_priscus_BS286_Sim_49500_1300	Bison_bonasus_U12954_0_0	Bos_taurus_DQ124388_T3_0_0
Bison_bison_BS569_AB_3600_70	Bison_priscus_BS287_BlR_49100_1700	Bison_bonasus_U34294_0_0	Bos_taurus_DQ124394_T3_0_0
Bison_bison_BS570_AB_11300_290	Bison_priscus_BS289_BlR_2172_37	**Yak**	Bos_taurus_DQ124398_T3_0_0
Bison_bison_BS99_26_5	Bison_priscus_BS291_NS_49700_1400	Bos_grunniens_AY521140_0_0	Bos_taurus_DQ124400_T4_0_0
Bison_bison_U12935_0_0	Bison_priscus_BS292_NS_35710_730	Bos_grunniens_AY521149_0_0	Bos_taurus_DQ124401_T4_0_0
Bison_bison_U12936_0_0	Bison_priscus_BS294_BlR_58200_3900	Bos_grunniens_AY521150_0_0	Bos_taurus_DQ124412_T4_0_0
Bison_bison_U12941_0_0	Bison_priscus_BS297_NS_10990_50	Bos_grunniens_AY521151_0_0	Bos_taurus_EU177822_T3_0_0
Bison_bison_U12943_0_0	Bison_priscus_BS311_BlR_12425_45	Bos_grunniens_AY521152_0_0	Bos_taurus_EU177841_T1_0_0
Bison_bison_U12944_0_0	Bison_priscus_BS316_SI_57700_3000	Bos_grunniens_AY521154_0_0	Bos_taurus_EU177842_T1_0_0
Bison_bison_U12945_0_0	Bison_priscus_BS318_NS_12410_50	Bos_grunniens_AY521155_0_0	Bos_taurus_EU177845_T1_0_0
Bison_bison_U12946_0_0	Bison_priscus_BS320_SI_49600_1500	Bos_grunniens_AY521156_0_0	Bos_taurus_EU177847_T1_0_0
Bison_bison_U12947_0_0	Bison_priscus_BS321_AK_9506_38	Bos_grunniens_AY521160_0_0	Bos_taurus_EU177848_T1_0_0
Bison_bison_U12948_0_0	Bison_priscus_BS323_SI_37810_380	Bos_grunniens_AY521161_0_0	Bos_taurus_EU177853_T2_0_0
Bison_bison_U12955_0_0	Bison_priscus_BS327_D_31530_230	Bos_grunniens_DQ007210_0_0	Bos_taurus_EU177854_T2_0_0
Bison_bison_U12956_0_0	Bison_priscus_BS328_SIdy_31690_180	Bos_grunniens_DQ007221_0_0	Bos_taurus_EU177860_T2_0_0
Bison_bison_U12957_0_0	Bison_priscus_BS329_D_27060_190	Bos_grunniens_DQ007222_0_0	Bos_taurus_EU177861_T2_0_0
Bison_bison_U12958_0_0	Bison_priscus_BS337_CHL_10378_36	Bos_grunniens_DQ856594_0_0	Bos_taurus_EU177862_T5_0_0
Bison_bison_U12959_0_0	Bison_priscus_BS340_NS_24500_180	Bos_grunniens_DQ856599_0_0	Bos_taurus_EU177863_T5_0_0
**Steppe bison**	Bison_priscus_BS345_NS_39800_1200	Bos_grunniens_DQ856600_0_0	Bos_taurus_EU177864_T5_0_0
Bison_priscus_A3133_Yukon_26360_220	Bison_priscus_BS350_NS_38700_1000	Bos_grunniens_DQ856603_0_0	Bos_taurus_EU177865_T5_0_0
Bison_priscus_BS105_F_23380_460	Bison_priscus_BS351_BlR_57700_3200	Bos_grunniens_DQ856604_0_0	**Buffalo**
Bison_priscus_BS107_F_19570_290	Bison_priscus_BS359_NTC_20020_150	Bos_grunniens_EF494177_0_0	Bubalus_bubalis_AF197208_0_0
Bison_priscus_BS108_F_21020_360	Bison_priscus_BS364_NS_38800_1100	Bos_grunniens_EF494178_0_0	Bubalus_bubalis_AF475212_0_0
Bison_priscus_BS109_F_20730_350	Bison_priscus_BS365_NS_47000_2900	**Zebu**	Bubalus_bubalis_AF475256_0_0
Bison_priscus_BS111_F_21580_370	Bison_priscus_BS387_NS_33320_540	Bos_indicus_AB085923_0_0	Bubalus_bubalis_AF475259_0_0
Bison_priscus_BS121_F_19360_280	Bison_priscus_BS388_NS_27590_280	Bos_indicus_AB268563_0_0	Bubalus_bubalis_AF475278_0_0
Bison_priscus_BS123_BlR_1730_60	Bison_priscus_BS389_NS_17160_80	Bos_indicus_AB268564_0_0	Bubalus_bubalis_AY488491_0_0
Bison_priscus_BS124_BlR_11900_70	Bison_priscus_BS390_NS_31630_440	Bos_indicus_AB268566_0_0	Bubalus_bubalis_EF536327_0_0
Bison_priscus_BS125_F_27440_790	Bison_priscus_BS392_NS_36320_780	Bos_indicus_AB268571_0_0	Bubalus_bubalis_EF536328_0_0
Bison_priscus_BS126_F_19150_280	Bison_priscus_BS393_NS_39850_1200	Bos_indicus_AB268574_0_0	Bubalus_bubalis_EU268899_0_0
Bison_priscus_BS130_BlR_9000_250	Bison_priscus_BS394_NS_37460_890	Bos_indicus_AB268578_0_0	Bubalus_bubalis_EU268909_0_0
Bison_priscus_BS133_F_33800_1900	Bison_priscus_BS395_NS_40700_1300	Bos_indicus_AB268580_0_0	
Bison_priscus_BS145_NS_12270_50	Bison_priscus_BS396_NS_23680_170	Bos_indicus_AY378134_0_0	

Supplementary Table 5. List of published whole mitochondrial genome sequences used for phylogenetic analysis.

**American bison**	**Cattle**	**Yak**
GU947000_Bison_bison_Plains_Nebraska_0	FJ971080_Bos_Q_Italy_Romagnola_0	KJ704989_Bos_grunniens_ChinaGansu_Gannan_0
GU946976_Bison_bison_Plains_Montana_0	FJ971085_Bos_R_Italy_Cinisara_0	KR011113_Bos_grunniens_ChinaTibet_QinghaiPlateau_0
GU947004_Bison_bison_Plains_Wyoming_0	EU177841_Bos_T1_Italy_chianina_0	KR052524_Bos_grunniens_ChinaTibet_Pali_0
GU947006_Bison_bison_Wood_ElkIsland_0	DQ124383_Bos_T2_Korea_0	KJ463418_Bos_grunniens_ChinaQinghai_Dantong_0
GU946987_Bison_bison_Plains_Montana_0	EU177815_Bos_T3_Italy_piemontese_0	KM233417_Bos_mutus_ChinaTibet_Yakow_0
GU947005_Bison_bison_Wood_ElkIsland_0	DQ124372_Bos_T4_Korea_0	**Buffalo**
GU947002_Bison_bison_Plains_Texas_0	EU177862_Bos_T5_Italy_valdostana_0	GU947003_Bison_bison_Plains_Texas_0
GU947003_Bison_bison_Plains_Texas_0	**Aurochs**	AY488491_Bubalus_bubalis
**Wisent**	GU985279_Bos_P_England_6760	AY702618_Bubalus_bubalis
JN632602_Bison_bonasus_0	JQ437479_Bos_P_Poland_1500	AF547270_Bubalus_bubalis
HQ223450_Bison_bonasus_0	**Zebu**	
HM045017_Bison_bonasus_Poland_0	FJ971088_Bos_I1_Mongolia_0	
**Steppe bison**	EU177870_Bos_I2_Iran_0	
KM593920_Bison_priscus_SGE2_France_TroisFreres_19151		

Supplementary Table 6. f4 ratio estimates, f4(A,O,X,C) is the numerator, f4(A,O,B,C) is the denominator.

S6-A. Including heterozygotes

**A**	**O**	**X**	**C**	**:**	**A**	**O**	**B**	**C**	**alpha**	**std.err**	**Z**
AmericanBison	Ovis_aries	AllWisent+CladeX	Aurochs	:	AmericanBison	Ovis_aries	Steppe	Aurochs	0.890988	0.025788	34.551
AmericanBison	Ovis_aries	AllWisent+CladeX	Steppe	:	AmericanBison	Ovis_aries	Aurochs	Steppe	0.109012	0.025788	4.227
AmericanBison	Ovis_aries	AllWisent	Aurochs	:	AmericanBison	Ovis_aries	Steppe	Aurochs	0.884257	0.02918	30.304
AmericanBison	Ovis_aries	AllWisent	Steppe	:	AmericanBison	Ovis_aries	Aurochs	Steppe	0.115743	0.02918	3.967
AmericanBison	Ovis_aries	CladeX	Aurochs	:	AmericanBison	Ovis_aries	Steppe	Aurochs	0.893978	0.022763	39.273
AmericanBison	Ovis_aries	CladeX	Steppe	:	AmericanBison	Ovis_aries	Aurochs	Steppe	0.106022	0.022763	4.658
AmericanBison	Ovis_aries	AncientWisent	Aurochs	:	AmericanBison	Ovis_aries	Steppe	Aurochs	0.812638	0.054701	14.856
AmericanBison	Ovis_aries	AncientWisent	Steppe	:	AmericanBison	Ovis_aries	Aurochs	Steppe	0.187362	0.054701	3.425
AmericanBison	Ovis_aries	HistoricalWisent	Aurochs	:	AmericanBison	Ovis_aries	Steppe	Aurochs	0.773802	0.032319	23.943
AmericanBison	Ovis_aries	HistoricalWisent	Steppe	:	AmericanBison	Ovis_aries	Aurochs	Steppe	0.226198	0.032319	6.999
AmericanBison	Ovis_aries	ModernWisent	Aurochs	:	AmericanBison	Ovis_aries	Steppe	Aurochs	0.899149	0.031184	28.834
AmericanBison	Ovis_aries	ModernWisent	Steppe	:	AmericanBison	Ovis_aries	Aurochs	Steppe	0.100851	0.031184	3.234

S6-B. Haploidisation by randomly sampling an allele at heterozygous sites

**A**	**O**	**X**	**C**	**:**	**A**	**O**	**B**	**C**	**alpha**	**std.err**	**Z**
AmericanBison	Ovis_aries	AllWisent+CladeX	Aurochs	:	AmericanBison	Ovis_aries	Steppe	Aurochs	0.894329	0.027147	32.944
AmericanBison	Ovis_aries	AllWisent+CladeX	Steppe	:	AmericanBison	Ovis_aries	Aurochs	Steppe	0.105671	0.027147	3.893
AmericanBison	Ovis_aries	AllWisent	Aurochs	:	AmericanBison	Ovis_aries	Steppe	Aurochs	0.88342	0.030518	28.947
AmericanBison	Ovis_aries	AllWisent	Steppe	:	AmericanBison	Ovis_aries	Aurochs	Steppe	0.11658	0.030518	3.82
AmericanBison	Ovis_aries	CladeX	Aurochs	:	AmericanBison	Ovis_aries	Steppe	Aurochs	0.912424	0.025204	36.202
AmericanBison	Ovis_aries	CladeX	Steppe	:	AmericanBison	Ovis_aries	Aurochs	Steppe	0.087576	0.025204	3.475
AmericanBison	Ovis_aries	AncientWisent	Aurochs	:	AmericanBison	Ovis_aries	Steppe	Aurochs	0.813521	0.059078	13.77
AmericanBison	Ovis_aries	AncientWisent	Steppe	:	AmericanBison	Ovis_aries	Aurochs	Steppe	0.186479	0.059078	3.156
AmericanBison	Ovis_aries	HistoricalWisent	Aurochs	:	AmericanBison	Ovis_aries	Steppe	Aurochs	0.786183	0.035363	22.232
AmericanBison	Ovis_aries	HistoricalWisent	Steppe	:	AmericanBison	Ovis_aries	Aurochs	Steppe	0.213817	0.035363	6.046
AmericanBison	Ovis_aries	ModernWisent	Aurochs	:	AmericanBison	Ovis_aries	Steppe	Aurochs	0.899281	0.032252	27.883
AmericanBison	Ovis_aries	ModernWisent	Steppe	:	AmericanBison	Ovis_aries	Aurochs	Steppe	0.100719	0.032252	3.123

Supplementary Table 7: Bootstrap resampling of genotypes for testing topologies using D statistics. The table shows the fraction of bootstrap replicates for which the original result was not recapitulated, from 10000 bootstraps, for 10%, 20%, etc. subsets of the genotypes. A topology is considered to be simple if it either has a non-significant D statistic (see Supplementary Figure 11), or has a D statistic closest to zero with confidence intervals that do not overlap the D statistic for the other two topologies.

Most parsimonious topology	Simple topology	10%	20%	30%	40%	50%	60%	70%	80%	90%
((CladeX, Steppe), ModernWisent)	True	0.0067	0.0001	0.0	0.0	0.0	0.0	0.0	0.0	0.0
((Steppe, HistoricalWisent), ModernWisent)	False	0.0575	0.0573	0.0284	0.0036	0.0005	0.0	0.0	0.0	0.0
((ModernWisent, CladeX), HistoricalWisent)	False	0.1753	0.371	0.485	0.4427	0.3039	0.1564	0.0549	0.0072	0.0

((CladeX, Steppe), HistoricalWisent)	True	0.0182	0.0174	0.0154	0.016	0.0113	0.0072	0.0022	0.0004	0.0
((AncientWisent, HistoricalWisent), ModernWisent)	True	0.0565	0.0152	0.0042	0.0012	0.0	0.0	0.0	0.0	0.0
((Steppe, HistoricalWisent), AncientWisent)	False	0.0151	0.0039	0.0001	0.0002	0.0	0.0	0.0	0.0	0.0
((AncientWisent, Steppe), ModernWisent)	True	0.0484	0.0086	0.0014	0.0002	0.0	0.0	0.0	0.0	0.0
((CladeX, Steppe), AncientWisent)	False	0.0304	0.0142	0.0086	0.0063	0.0033	0.0025	0.0015	0.0001	0.0
((AncientWisent, CladeX), ModernWisent)	True	0.0703	0.0213	0.0062	0.0015	0.0007	0.0	0.0	0.0	0.0
((HistoricalWisent, CladeX), AncientWisent)	False	0.0184	0.0053	0.001	0.0005	0.0	0.0	0.0	0.0	0.0
((ModernWisent, HistoricalWisent), Aurochs)	False	0.0591	0.0031	0.0005	0.0	0.0	0.0	0.0	0.0	0.0
((Aurochs, ModernWisent), CladeX)	False	0.2229	0.2476	0.0824	0.0115	0.0009	0.0	0.0	0.0	0.0
((HistoricalWisent, CladeX), Aurochs)	True	0.0061	0.0	0.0	0.0	0.0	0.0	0.0	0.0	0.0
((Steppe, CladeX), Aurochs)	True	0.0001	0.0	0.0	0.0	0.0	0.0	0.0	0.0	0.0
((Steppe, HistoricalWisent), Aurochs)	True	0.0	0.0	0.0	0.0	0.0	0.0	0.0	0.0	0.0
((Steppe, ModernWisent), Aurochs)	False	0.1362	0.0535	0.0048	0.0007	0.0002	0.0	0.0001	0.0	0.0
((Steppe, AncientWisent), Aurochs)	True	0.0441	0.0082	0.0001	0.0001	0.0	0.0	0.0	0.0	0.0
((AncientWisent, CladeX), Aurochs)	True	0.0276	0.0058	0.0004	0.0001	0.0	0.0	0.0	0.0	0.0

Supplementary Table 8: Hypergeometric test for shared derived steppe alleles. Steppe derived sites were filtered for coverage depth in the wisent lineages 1 and 2, for which the test was performed. In the last row, wisent represents all wisent other than CladeX.

1	2	Steppe	Derived 1	Derived 2	Common	P
Ancient Wisent	CladeX	161	111	133	108	1.72E-12
Ancient Wisent	Historical Wisent	174	115	119	108	1.37E-24
Ancient Wisent	Modern Wisent	178	124	108	95	5.12E-11
CladeX	Historical Wisent	529	448	385	370	3.09E-29
CladeX	Modern Wisent	556	469	350	326	2.79E-13
Historical Wisent	Modern Wisent	618	436	372	342	5.50E-48
Wisent	CladeX	557	357	468	332	4.18E-14

Supplementary Table 9: Hypergeometric test for shared derived aurochs alleles. Aurochs derived sites were filtered for coverage depth in the wisent lineages 1 and 2, for which the test was performed. In the last row, wisent represents all wisent other than CladeX.

**1**	**2**	**Aurochs**	**Derived 1**	**Derived 2**	**Common**	**P**
Ancient Wisent	CladeX	758	20	9	4	4.11E-05
Ancient Wisent	Historical Wisent	822	22	11	8	1.01E-11
Ancient Wisent	Modern Wisent	826	25	22	12	1.49E-14
CladeX	Historical Wisent	2517	36	47	16	7.34E-20
CladeX	Modern Wisent	2580	39	73	15	1.99E-14
Historical Wisent	Modern Wisent	2845	58	83	39	2.66E-50
Wisent	CladeX	2634	93	41	15	1.58E-12

Supplementary Table 10: The weighted sample median $\hat{M}$, the weighted sample mode $\hat{Mo}$, and the prediction error E_{pred}, for each ABC analysis.

**Trio**	$\hat{\mathbf{M}}$	$\hat{\mathbf{Mo}}$	$\mathbf{E_{pred}}$
A875, 6A, Aurochs	0.8660	0.9204	0.4534
A3133, 6A, Aurochs	0.8480	0.9172	0.4881
A875, Historical Wisent, Aurochs	0.8636	0.9323	0.4187
A3133, Historical Wisent, Aurochs	0.8646	0.9384	0.4921
All	0.8250	0.9034	0.5111

Supplementary Table 11: Empirical posterior probabilities for levels of hybridisation 1%-5%, for each trio.

**Trio**	**1%**	**2%**	**3%**	**4%**	**5%**
A875, 6A, Aurochs	0.9620	0.9340	0.8720	0.8400	0.8120
A3133, 6A, Aurochs	0.9600	0.9600	0.8840	0.8440	0.7980
A875, Historical Wisent, Aurochs	0.9660	0.9340	0.8860	0.8520	0.7940
A3133, Historical Wisent, Aurochs	0.9580	0.9100	0.8580	0.8080	0.7640
All	0.9720	0.9440	0.9140	0.8760	0.8760

Supplementary Note 1:

Samples, DNA extraction and sequencing

Samples and radiocarbon dating

For clarity purposes we kept the most commonly used taxonomic nomenclature of bovine throughout the study. Although not yet widely accepted, it has been proposed to sink the genus *Bison* into *Bos* based on the shallow time depth of their evolutionary history [7]. The validity of such genetic separation is further tested in this study.

Samples from a total of 87 putative bison bones were collected from 3 regions across Europe: Urals, Caucasus, and Western Europe (Supplementary Data 1). As shown in the Supplementary Data 1, most of the samples were from bones identified as bison or bovid post-cranial samples, because cranial material is rare for this time period.

The main set of samples, from northeastern Europe, represents isolated bones excavated from a wide variety of cave deposits throughout the Ural Mountains and surrounding areas. These samples are housed at the Zoological Museum of the Institute of Plant and Animal Ecology (ZMIPAE) in Ekaterinburg, Russia.

In southeastern Europe, bovid bone fragments were excavated in Mezmaiskaya Cave in the Caucasus Mountains. Samples were obtained from the Laboratory of Prehistory in St Petersburg. Additional six samples from the Caucasus are identified as Caucasian bison (B. bonasus caucasicus, hereafter referred to as historical wisent): two of them are from the National History Museum (NHM) in London, and four come from hunts in the Kuban Oblast in the early 20th century (one collected by scientist Viktor Iwanovich Worobjew in 1906 and three hunted during the Kuban Hunt under the Grand Duke Sergei Mikhailovich of Russia), currently held at the Zoological Institute of the Russian Academy of Sciences (ZIRAS - Saint Petersburg, Russia). Four additional bones from the Caucasus region comes from the eastern border with Ukraine and are held at the Institute of Archeology (IAKiev), Ukrainian Academy of Sciences, Kiev.

Most western European bones come from late Pleistocene deposits on the North Sea bed. These specimens, now curated by the North Sea Network (NSN) in the Netherlands, were recovered by trawling operations and as such have little stratigraphic information. Specimens were selected on the basis of their morphological similarities with the 'small form' described by Drees and Post [8].

Three bones held in the collections of the Vienna Natural History Museum (VNHM), and three bones held in the Museum National d'Histoire Naturelle (Paris) come from central European Holocene sites.

Finally, one bone comes from the Monti Lessini rock-shelter site Riparo Tagliente in the North of Italy, one bone comes from the Swiss site of Le Gouffre de la combe de la racine in the Jura mountains (Swiss Institute for Speleology and Karst Studies, ISSKA), and one bone comes from l'Aven de l'Arquet in the Gard region of France (Musée de Préhistoire d'Orgnac).

In addition, two samples from the Beringian region were used: one sample, a steppe bison astragalus from the Yukon territory (Canada), has previously been used in a study of cytosine methylation in ancient DNA [9]; and another steppe bison from Alyoshkina Zaimka in Siberia.

All non-contemporaneous samples from which bison mitochondrial control region sequences were successfully amplified were sent for accelerator mass spectrometry (AMS) radiocarbon dating (except for seven samples from level 3 of the Mezmaiskaya cave, which were expected to be older than AMS dating capabilities [10,11]). The dating was performed by the AMS facility at the Oxford Radiocarbon Accelerator Unit at the University of Oxford (OxA numbers), the Eidgenössische Technische Hochschule in Zürich for a Ukrainian sample (ETH number), and the Ångström Laboratory of the University of Uppsala, Sweden, for the Swiss sample (Ua number). The results are shown in Supplementary Data 1, with all dates reported in kcal yr BP unless otherwise stated. The calibration of radiocarbon dates was performed using OxCal v4.1 with the IntCal13 curve [12].

In addition, two bones identified as bison were previously dated at the Centre for Isotope Research, Radiocarbon Laboratory, University of Groningen, Netherlands, with infinite radiocarbon age, consistently with the dating performed at Oxford (A2808-JGAC26=GrA-34533; A2809-JGAC27= GrA-34524).

Ancient DNA extraction

All ancient DNA work was conducted in clean-room facilities at the University of Adelaide's Australian Centre for Ancient DNA, Australia (ACAD), and at the University of Tuebingen, Germany (UT) following published guidelines [13].

<u>University of Adelaide:</u>

Samples were UV irradiated (260 nm) on all surfaces for 30 min. Sample surface was wiped with 3% bleach, then ~1 mm was removed using a Dremel tool and carborundum cutting disks. Each sample was ground to a fine powder using a Mikro-Dismembrator (Sartorius). Two DNA extraction methods were used during the course of the project (see Supplementary Data 1for the method used for specific samples):

- *Phenol-chloroform method*: Ancient DNA was extracted from 0.2-0.5g powdered bone using phenol-chloroform and centrifugal filtration methods according to a previously published method [2].

- *In solution silica based method*: Ancient DNA was extracted from 0.2-0.3g powdered bone according to a previously published method [14].

<u>University of Tuebingen:</u>

Samples were UV-irradiated overnight to remove surface contamination. DNA extraction was performed following a guanidinium-silica based extraction method [15] using 50mg of bone powder. A DNA library was prepared using 20µl of extract for each sample according to [16]. Sample-specific indexes were added to both library adapters to differentiate between individual samples after pooling and multiplex sequencing [17]. Indexed libraries were amplified in 100µl reactions, followed by purification over Qiagen MinElute spin columns (Quiagen, Hilden, Germany).

Sequencing of the mitochondrial control region

A ~600 bp fragment of the mitochondrial control region was amplified in one or up to four overlapping fragments, depending on DNA preservation. PCR amplifications were performed using primers designed for the bovid mitochondrial control region, following the method described in [2].

One-step simplex PCR amplifications using Platinum *Taq* Hi-Fidelity polymerase were performed on a heated lid thermal cycler in a final volume of 25 µl containing 1 µl of aDNA extract, 1mg/ml rabbit serum albumin fraction V (RSA; Sigma-Aldrich, Sydeny, NSW), 2 mM $MgSO_4$ (Thermo Fisher, Scoresby VIC), 0.6 µM of each primer (Supplementary Table 1), 250 µM of each dNTP (Thermo Fisher), 1.25 U Platinum *Taq* Hi-Fidelity and 1 × Hi-Fidelity PCR buffer (Thermo Fisher). The conditions for PCR amplification were initial denaturation at 95°C for 2 min, followed by 50 cycles of 94°C for 20 sec, 55°C for 20 sec and 68°C for 30 sec, and a final extension at 68°C for 10 min at the end of the 50 cycles.

Multiplex primer sets A and B were set up separately (Supplementary Table 1). Multiplex PCR was performed in a final volume of 25 µl containing 2 µl of aDNA extract, 1 mg/ml RSA, 6 mM $MgSO_4$, 0.2 µM of each primer (Supplementary Table 1), 500 µM of each dNTP, 2 U Platinum *Taq* Hi-Fidelity and 1 × Hi-Fidelity PCR buffer. Multiplex PCR conditions were initial denaturation at 95°C for 2 min, followed by 35 cycles of 94°C for 15 sec, 55°C for 20 sec and 68°C for 30 sec, and a final extension at 68°C for 10 min at the end of the 35 cycles. Multiplex PCR products were then diluted to 1:10 as template for the second step of simplex PCR. The simplex PCR, using Amplitaq Gold (Thermo Fisher) or Hotmaster™ *Taq* DNA polymerase (5Prime, Milton, Qld), was conducted in a final volume of 25 µl containing 1 µl of diluted multiplex PCR product, 2.5 mM $MgCl_2$, 0.4 µM of each primer (Supplementary Table 1), 200 µM of each dNTP, 1 U Amplitaq Gold/Hotmaster *Taq* polymerase and 1 × PCR buffer. The PCR conditions were initial denaturation at 95°C for 2 min, followed by 35 cycles of 94°C for 20 sec, 55°C for 15 sec and 72°C for 30 sec, and a final extension at 72°C for 10 min at the end of the 35 cycles. Multiple PCR fragments were cloned to evaluate the extent of DNA damage and within-PCR template diversity.

PCR products were then checked by electrophoresis on 3.5-4.0% agarose TBE gels, and visualized after ethidium bromide staining on a UV transilluminator. PCR amplicons were purified using Agencourt® AMPure magnetic beads (Beckman Coulter, Lane Cove, NSW) according to the manufacturer's instructions. Negative extraction controls and non-template PCR controls were used in all experiments.

All purified PCR products were bi-directionally sequenced with the ABI Prism® BigDye™ Terminator Cycle Sequencing Kit version 3.1 (Thermo Fisher). The sequencing reactions were performed in a final volume of 10 µl containing 3.2 pmol of primer (Supplementary Table 1), 0.25 µl Bigdye terminator premixture, and 1.875 µl of 5 × sequencing buffer. The reaction conditions included initial denaturation at 95°C for 2 min, 25 cycles with 95°C for 10 sec, 55°C for 15 sec, and 60°C for 2 min 30 sec. Sequencing products were purified using Agencourt® Cleanseq magnetic beads (Beckman Coulter) according to the manufacturer's protocol. All sequencing reactions were analysed on an ABI 3130 DNA capillary sequencer (Thermo Fisher).

Mitochondrial control region sequences (>400bp) were successfully amplified from 65 out of 87 analysed samples. Three samples produced a mixture of cattle and bison

amplification products; these were identified as contaminated and removed from all analyses. Sequences from two individuals did not match bovid haplotypes and were identified as brown bear and elk in BLAST searches (see Supplementary Data 1). This is presumably due to the source postcranial elements being morphologically ambiguous and misidentified.

Sequencing of the whole mitochondrial genome

To provide deeper phylogenetic resolution and further examine the apparent close relationship between *Bos* and wisent mitochondria, full mitogenome sequences of 13 CladeX specimens, as well as one ancient wisent, one historical wisent, and one steppe bison were generated using hybridisation capture with RNA probes.

Samples A001, A004, A018, A4089 (CladeX)

DNA library preparation

DNA repair and polishing were performed in a reaction that contained 20 µl DNA extract, 1x NEB Buffer 2 (New England Biolabs, Ipswich, MA), 3U USER enzyme cocktail (New England Biolabs), 20U T4 polynucleotide kinase (New England Biolabs), 1mM ATP, 0.1 mM dNTPs (New England Biolabs), 8 µg RSA, and H_2O to 38.5 µl. The reaction was incubated at 37°C for 3 hours then 4.5U of T4 DNA polymerase (New England Biolabs) was added and the reaction incubated at 25°C for a further 30 min. Double-stranded libraries were then built with truncated Illumina adapters containing dual 5-mer internal barcodes as in [16].

Amplification of Bos taurus mitochondrial in vitro transcription (IVT) templates

RNA probes were generated from long-range PCR products of *Bos taurus* mitochondrial DNA. The NCBI Primer-Blast program (http://www.ncbi.nlm.nih.gov/tools/primer-blast/) was used to design primers to amplify the *Bos taurus* mitochondrial genome (NC_006853.1) in three overlapping sections: mito-1 (6568 bp), mito-2 (6467 bp), and mito-3 (5390 bp). Primer pairs were designed with a high melting temperature to permit amplification with 2-stage PCR and the T7 RNA promoter was attached to the 5' end of one primer from each pair [18](Supplementary Table 1). Amplification of each mitochondrial section was performed using a heated lid thermal cycler in multiple PCRs containing 1x Phire Buffer (Thermo Fisher), 25 ng calf thymus DNA (Affymetrix, Santa Clara, CA), 200 µM dNTPs , 500 nM forward and reverse primers, 0.5 µl Phire Hot Start II DNA polymerase (Thermo Fisher), and H_2O to 25 µl. The mito-1 and mito-2 sections were amplified with a thermal cycler program of 1 cycle: 98°C for 30 sec; 26 cycles: 98°C for 10 sec and 72°C for 70 sec; and 1 cycle: 72°C for 180 sec whilst the program for mito-3 was 1 cycle: 98°C for 30 sec, 28 cycles: 98°C for 10 sec and 72°C for 60 sec, and 1 cycle: 72°C for 180 sec. After amplification, 2 □l of each PCR was agarose gel electrophoresed and the product visualized with Gel-Red (Biotium, Hayward, CA) staining and UV illumination. Amplification of mito-1 and mito-2 produced a single band and the PCRs for these mitochondrial sections were separately pooled and then purified with QiaQuick columns (Qiagen, Chadstone Centre, VIC) following the provided PCR cleanup protocol. Amplification of mito-3 produced unwanted products and the correct size amplicon was size selected using gel excision followed

by purification with QiaQuick columns using the gel extraction protocol. Purified amplicons from each mitochondrial section were quantified using a NanoDrop 2000 Spectrophotometer (Thermo Fisher).

Transcription of Bos taurus mitochondrial IVT templates

Each of the three mitochondrial IVT templates were transcribed using a T7 High Yield RNA Synthesis Kit (New England Biolabs) in multiple reactions containing 150-200 ng purified amplicon, 1x Reaction Buffer, 10 mM rNTPs, 2 µl T7 enzyme mix, and H_2O to 20 µl. The IVT reactions were incubated for 16 hours at 37°C and then the DNA template was destroyed by incubating for an additional 15 min at 37°C with 2U Turbo Dnase (Thermo Fisher). IVT reactions for each mitochondrial section were separately pooled and purified with Megaclear spin columns (Thermo Fisher) except that H_2O was used to elute the RNA instead of the provided elution buffer. The elution buffer provided with the Megaclear kit was found to inhibit fragmentation in the next step. Integrity of the RNA was verified on an acrylamide gel and the mass quantified with a Nanodrop 2000 Spectrophotometer.

Fragmentation of mitochondrial IVT RNA

RNAs from the IVT transcription were fragmented with a NEBNext Magnesium RNA Fragmentation Module (New England Biolabs) in reactions that contained 1x Fragmentation buffer, 45 µg RNA, and H_2O to 20 µl. Reactions were incubated at 94°C for 10 min and fragmentation stopped with the addition of 2 µl Stop Buffer. After fragmentation, each reaction was purified with a RNeasy MinElute spin column (Qiagen) by following the provided cleanup protocol except for the final elution. To elute, 20 µL H_2O was pipetted into the column and the column was heated at 65°C for 5 min and then centrifuged at 15,000 g for 1 min. The flow-through was transferred to a 1.5 ml tube and stored at -80°C. The fragmented RNA was quantified on a NanoDrop 2000 Spectrophotometer and 100 ng was visualized on an acrylamide gel producing a smear in the range of 80-300 bases.

Biotinylation of fragmented RNA

Biotinylation was performed in several reactions containing 6.7 µg each of mito-1, mito-2, and mito-3 fragmented RNA, 40 µl Photoprobe Long Arm (Vector Laboratories, Burlingame, CA), and H_2O to 80 µl in 200 µl PCR tubes. The tubes were placed in a 4°C gel cooling rack and then incubated under the bulb of a UV sterilization cabinet for 30 min. Organic extractions were performed on the labelling reactions by adding 64 µl H_2O, 16 µl 1 M Tris buffer, and 160 µl sec-butanol to each tube and shaking vigorously for 30 sec followed by centrifugation for 1 minute at 1000 g. The upper organic layers were discarded and the extraction repeated with an additional 160 µl sec-butanol. After the second organic layers were discarded, the remaining aqueous phases were purified with RNeasy MinElute spin columns following the provided reaction cleanup protocol but with a modified elution procedure described in the previous step. Elutions with similar RNA were pooled and then quantified with a NanoDrop Spectrophotometer 2000 and the RNA, which will now be called probe, was stored at -80°C in 5 µl aliquots at 100 ng/µl.

Repetitive sequence blocking RNA

RNA to block repetitive sequences in bison aDNA was transcribed from Bovine HyBlock™ DNA (i.e. Cot-1 DNA, Applied Genetics Laboratories Inc., Melbourne, FL) using a published linear amplification protocol [19]. Briefly, the HyBlock DNA was polished in a reaction containing T4 polynucleotide kinase and T4 DNA polymerase and purified with MinElute spin columns following the PCR cleanup protocol provided. Tailing was performed on the polished DNA with terminal transferase and a tailing solution containing 92 µM dTTP (Thermo Fisher) and 8 µM ddCTP (Affymetrix). After tailing, the Hybloc DNA was purified with MinElute spin columns as before. The HyBlock DNA was then heat denatured and the T7-A18B primer (Supplementary Table 1), containing the T7 RNA polymerase promoter, was allowed to anneal to the poly-T tail with slow cooling. A second-strand synthesis reaction was then performed on the HyBlock DNA using DNA polymerase I Klenow fragment (New England Biolabs) and the product was purified with MinElute spin columns. The double stranded HyBlock DNA was transcribed using a T7 High Yield RNA Synthesis Kit in multiple reactions containing 75 ng DNA, 1x Reaction Buffer, 10 mM rNTPs, 2 µl T7 enzyme mix, and H_2O to 20 µl. IVT reactions were incubated for 16 hours at 37°C and then the DNA template was destroyed by adding 2U Turbo Dnase and incubating for an additional 15 min at 37°C. The RNA was purified with RNeasy MinElute spin columns as above. Purified RNA was quantified on a NanoDrop 2000 and 100 ng visualized on an acrylamide gel, which produced a smear 80 to 500 bp in length.

Primary mitochondrial hybridisation capture

Truncated versions of the Illumina adapters were used for hybridisation capture because full-length adapters reduce enrichment efficiency [20]. For the primary hybridisation capture, three Reagent Tubes were prepared for each bison library with the following materials: Reagent Tube #1- 3.5 µl of 35-55 ng/µl DNA library; Reagent Tube #2- 5 µl probes, 1 µl HyBlock RNA, and 0.5 µl of 50 µM P5/P7 RNA blocking oligonucleotides (Supplementary Table 1); Reagent Tube #3- 30 µl Hybridisation Buffer [21]: 75% formamide (Thermo Fisher), 75 mM HEPES , pH 7.3, 3 mM EDTA (Thermo Fisher), 0.3% SDS (Thermo Fisher), and 1.2 M NaCl (Thermo Fisher). Hybridisation capture was performed in a heated lid thermal cycler programmed as follows: Step 1- 94°C for 2 min, Step 2- 65°C for 3 min, Step 3- 42°C for 2 min, Hold 4- 42°C hold. To start hybridisation capture, Reagent Tubes were placed in the thermal cycler at the start of each program Step in the following order: Step 1- Reagent Tube #1; Step 2- Reagent Tube #2; Step 3- Reagent Tube #3. For each library, once the Hold cycle started 20 µl of hybridisation buffer from Reagent Tube #3 was mixed with the RNA in Reagent Tube #2. The entire content of Reagent Tube #2 was then pipetted into Reagent Tube #1 and mixed with the bison library to begin the hybridisation capture. Hybridisation capture was carried out at 42°C for 48 hours.

Magnetic streptavidin beads (New England Biolabs) were washed just prior to the end of the hybridisation capture incubation. For each library, 50 µl of beads were washed twice using 0.5 ml Wash Buffer 1(2X SSC+0.05% Tween-20, all reagents Thermo Fisher) and a magnetic rack. We also saturated all magnetic bead sites that could potentially bind nucleic acid in a non-specific fashion using yeast tRNA, to optimise the expected and specific streptavidin-biotin binding. Briefly, the beads were blocked

by incubation in 0.5 ml Wash Buffer 1+ 100 µg yeast tRNA (Thermo Fisher) for 30 min on a rotor. Blocked beads were washed once as before and then suspended in 0.5 ml Wash Buffer. At the end of the hybridisation capture, each reaction was added to a tube of blocked beads and incubated at room temperature for 30 min on a rotor. The beads were then taken through a series of stringency washes as follows: Wash 1 - 0.5 ml Wash Buffer 1 at room temperature for 10 min; Wash 2 - 0.5 ml Wash Buffer 2 (0.75X SSC + 0.05% Tween-20) at 50°C for 10 min; Wash 3 - 0.5 ml Wash Buffer 2 at 50°C for 10 min; Wash 4 - 0.5 ml Wash Buffer 3 (0.2X SSC + 0.05% Tween-20) at 50°C for 10 min. After the last wash, the captured libraries were released from the probe by suspending the beads in 50 µl of Release buffer (0.1 M NaOH, Sigma Aldrich) and incubating at room temperature for 10 min. The Release buffer was then neutralized with the addition of 70 µl Neutralization buffer (1 M Tris-HCl pH 7.5, Thermo Fisher). Captured libraries were then purified with MinElute columns by first adding 650 µl PB buffer and 10 µl 3 M sodium acetate to adjust the pH for efficient DNA binding. Libraries were purified using the provided PCR cleanup protocol and eluting with 35 µl EB+0.05% Tween-20.

Primary hybridisation capture amplification

Amplification of each primary hybridisation capture was performed in five PCRs containing 5 µl of primary captured library, 1X Phusion HF buffer (Thermo Fisher), 200 µM dNTPs, 200 µM each of primers IS7_short_amp.P5 and IS8_short_amp.P7 (Supplementary Table 1), 0.25 U Phusion Hot Start II DNA polymerase (Thermo Fisher), and H_2O to 25 µl. The five PCR products were pooled and DNA was purified using AMPure magnetic beads.

Secondary mitochondrial hybridisation capture

Amplified primary libraries were taken through a second round of hybridisation capture using the same procedure as describe in *Primary mitochondrial hybridisation capture* step.

Secondary hybridisation capture amplification

Indexed primers were used to convert the DNA from the secondary hybridisation capture to full length Illumina sequencing libraries. Each library was amplified in three PCRs containing 5 µl secondary hybridisation capture library, 1X Phusion HF buffer, 200 µM dNTPs, 200 µM each of primers GAII_Indexing_*x* (library specific index) and IS4 (Supplementary Table 1), 0.25 U Phusion Hot Start II DNA polymerase, and H_2O to 25 µl. Amplification was performed in a heated lid thermal cycler programmed as follows 1 cycle: 98°C for 30 sec; 10 cycles: 98°C for 10 sec, 60°C for 20 sec, 72°C for 20 sec; and 1 cycle: 72°C for 180 sec. The five PCR products were pooled and DNA was purified using AMPure magnetic beads.

<u>Samples A003, A005, A006, A007, A017, A15526, A15637, A15668 (CladeX), A4093 (*ancient wisent*) and A15654 (*historical wisent*)</u>

DNA library preparation

Double-stranded Illumina libraries were built from 20 µl of each DNA extract using

partial UDG treatment [22] and truncated Illumina adapters with dual 7-mer internal barcodes, following the protocol from [23].

Hybridisation capture

Commercially synthesised biotinylated 80-mer RNA baits (MYcroarray, MI, USA) were used to enrich the target library for mitochondrial DNA. Baits were designed as part of the commercial service using published mitochondrial sequences from 24 placental mammals, including *Bison bison* and *Bos taurus*.

One round of hybridisation capture was performed according to the manufacturer's protocol (MYbaits v2 manual) with modifications. We used P5/P7 RNA blocking oligonucleotides (Supplementary Table 1) instead of the blocking oligonucleotides provided with the kit. We also incubated the magnetic beads with yeast tRNA to saturate all potential non-specific sites on the magnetic beads that could bind nucleic acids and increase the recovery of non-specific DNA and therefore decrease the final DNA yield.

Indexed primers were used to convert the capture DNA to full length Illumina sequencing libraries. Each library was amplified in eight PCRs containing 5 µl hybridisation capture library, 1x Gold Buffer II, 2.5mM $MgCl_2$, 200 µM dNTPs, 200 µM each of primers GAII_Indexing_*x* (library specific index) and IS4 (Supplementary Table 1), 1.25 U Amplitaq Gold DNA polymerase, and H_2O to 25 µl. Amplification was performed in a heated lid thermal cycler programed as follows 1 cycle: 94°C for 6 min; 15 cycles: 98°C for 30 sec, 60°C for 30 sec, 72°C for 40 sec; and 1 cycle: 72°C for 180 sec. The PCR products were pooled and DNA was purified using AMPure magnetic beads (Agencourt®, Beckman Coulter).

Samples LE237, LE242 and LE257 (CladeX)

Target DNA enrichment was performed by capture of the pooled libraries using DNA baits generated from bison (*Bison bison*) mitochondrial DNA [24]. The baits were generated using three primer sets (Supplementary Table 1, f) designed with the Primer3Plus software package [25]. All extractions and pre-amplification steps of the library preparation were performed in clean room facilities and negative controls were included for each reaction.

Sample A3133 (steppe bison)

DNA repair and polishing were performed in a reaction that contained 20 µl bison A3133 extract, 1x NEB Buffer 2, 3U USER enzyme cocktail, 20U T4 polynucleotide kinase, 1mM ATP, 0.1 mM dNTPs, 8 µg RSA, and H_2O to 38.5 µl. The reaction was incubated at 37°C for 3 hours then 4.5U of T4 DNA polymerase was added and the reaction incubated at 25°C for a further 30 min. Double-stranded libraries were then built with truncated Illumina adapters containing dual 5-mer internal barcodes as in [16] with the final amplification with indexed primers using Phusion Hot Start II DNA polymerase to obtain full length Illumina sequencing libraries.

Nuclear locus capture

Genome-wide nuclear locus capture was attempted on DNA repaired libraries of 13 bison samples (as described above - see Supplementary Supplementary Table 2). Two different sets of probe were used (as described below), but ultimately, only the 9908 loci common to both sets were used for comparative analysis (see nuclear locus analysis section).

Probe sets

40k SNP probe set

This probe set was originally designed to enrich 39,294 of the 54,609 BovineSNP50 v2 BeadChip (Illumina) bovine single nucleotide polymorphism (SNP) loci used in a previous phylogenetic study [26], allowing for a direct comparison of the newly generated data to published genotypes. The discrepancy in the number of surveyed targets was due to manufacturing constraints, as the flanking sequences surrounding certain bovine SNP were too degenerate for synthesis with the MyBaits technology. Probes (MYcroarray, Ann Arbor, MI) were 121-mer long, centred on the targeted bovine SNP and with no tiling, as per the original design of the BovineSNP50 v2 BeadChip [27].

The BovineSNP50 v2 BeadChip assay targets SNPs that are variable in *Bos taurus* in order to genotype members of cattle breeds. Consequently, SNPs are heavily ascertained to be common in cattle, and their use in phylogenetic studies of other bovid species results in levels of heterozygosity that decrease rapidly with increased genetic distance between cattle and the species of interest. Decker et al. (2009) found the average minor allele frequency in plains bison and wood bison for the 40,843 bovine SNPs used in the phylogenetic analysis was 0.014 and 0.009, respectively. Average minor allele frequencies ranged from 0.139 to 0.229 in breeds of taurine cattle.

10k SNP probe set

A second set of probes was ordered from MyBaits that targeted a 9,908 locus subset of the previous 39,294 bovine SNPs selected for enrichment. This smaller subset was chosen to minimise ascertainment bias during phylogenetic and population analyses based on their polymorphism within the diversity of available modern genotypes of bison (American and European), Yak, Gaur and Banteng (total of 72 individuals). All of these taxa belong to a monophyletic clade, outside of the cattle diversity, and are consequently all equidistant from the cattle breeds that were used to ascertain the SNP [27], therefore reducing the impact of ascertainment bias when conducting comparisons within the clade. The exclusion of monomorphic sites across specie allows focusing the capture on loci that are more likely to be phylogenetically informative within the bison diversity. Furthermore, singleton sites (only variable for one modern individual, and therefore not informative for the modern phylogeny) were retained on the principle that they might capture some of the unknown ancient diversity of bison when genotyping ancient individuals.

We designed 70-mer probes, and this short length, as well as the limited number of targets, allowed for a tiling of 4 different probes for each targeted locus, within the same MYcroarray custom kit of 40,000 unique probes. Among all potential 70-mer

sequences within the original 121-mer probe sequence set, only those containing the targeted bovine SNP no fewer than 10 nucleotides from either end were retained as potential probes. Four probes were then designed using the following criteria: i) Estimated melting temperature closest to the average from the 40k SNP probe set; ii) Optimum proportion of guanine based on the efficiency of the 40k SNP probe set; iii) No two probes can be closer than 7 nucleotides from one another; iv) All 'GGGG' and 'CTGGAG' motifs were modified to 'GTGT' and 'CTGTAG', respectively. The former change was incorporated on the recommendation from MyBaits to avoid poly G stretches because their synthesis technology has difficulty with this type of motif and the latter variation was included to remove a restriction site that will be used in a future protocol to produce these probes from an immortalized DNA oligo library [28].

<u>DNA library preparation</u>

All DNA libraries were used for capture of both the mitochondrial genome and genome-wide nuclear loci. See Supplementary Information "Whole mitochondrial genome sequencing" for protocols.

<u>Hybridisation capture</u>

One round of hybridisation capture was performed according to the manufacturer's protocol (MYbaits v2 manual) with modifications. We used P5/P7 RNA blocking oligonucleotides (Supplementary Table 1) instead of the blocking oligonucleotides provided with the kit. We also incubated the magnetic beads with yeast tRNA (see above) to saturate all potential non-specific sites on the magnetic beads that could bind nucleic acids and increase the recovery of non-specific DNA.

Indexed primers were used to convert the capture DNA to full length Illumina sequencing libraries. Each library was amplified in eight PCRs containing 5 μl hybridisation capture library, 1C Gold Buffer II, 2.5mM $MgCl_2$, 200 μM dNTPs, 200 μM each of primers GAII_Indexing_*x* (library specific index) and IS4 (Supplementary Table 1), 1.25 U Amplitaq Gold DNA polymerase, and H_2O to 25 μl. Amplification was performed in a heated lid thermal cycler programed as follows 1 cycle: 94°C for 6 min; 15 cycles: 98°C for 30 sec, 60°C for 30 sec, 72°C for 40 sec; and 1 cycle: 72°C for 180 sec. The PCR products were pooled and DNA was purified using AMPure magnetic beads.

NGS and data processing

Whole mitochondrial genomes

All libraries enriched for the mitochondrial genome were sequenced in paired-end reactions on Illumina machines (HiSeq 2500 for LE237A, LE242B and LE247B – MiSeq for the rest), except for A017 and A15526 from which the final concentration of DNA obtained after capture was insufficient for sequencing. The mitochondrial genome of the steppe bison A3133 was recovered from shotgun sequencing on an Illumina HiSeq, performed in the context of another study (see Supplementary Table 3).

All NGS reads were processed using the pipeline Paleomix v1.0.1[29]. AdapterRemoval v2[30] was used to trim adapter sequences, merge the paired reads, and eliminate all

reads shorter than 25 bp. BWA v0.6.2[31] was then used to map the processed reads to the reference mitochondrial genome of the wisent (NC_014044) or the American bison (NC_012346, only for the steppe bison A3133). Minimum mapping quality was set at 25, seeding was disabled and the maximum number or fraction of gap opens was set to 2.

MapDamage v2[32] was used to check that the expected contextual mapping and damage patterns were observed for each library, depending on the enzymatic treatment used during library preparation (see Supplementary Table 3 and Figures S1-3 for examples), and re-scale base qualities for the non-repaired libraries.

Finally nucleotides at the position of the bovine SNP were called using samtools and bcftools, setting the minimum base quality at 30 and the minimum depth of coverage at 2. Consensus sequences were then generated using the Paleomix script vcf_to_fasta.

Nuclear

Nuclear DNA from historical (historical wisent: A15654) and ancient (ancient wisent: A4093; CladeX: A15526, A001, A003, A004, A005, A006, A007, A017, A018; steppe: A3133, A875) samples, containing HiSeq data (A3133 and A875) and MiSeq data (all samples), was processed using Paleomix v1.0.1[29] to map reads against the *Bos taurus* reference UMD 3.1[33]. Paleomix was configured to use BWA v0.6.2[31] for mapping, with seeding disabled and -n 0.01 -o 2 (see Supplementary Table 2). MapDamage v2[32] was used to check that the expected contextual mapping and damage patterns were observed for each library, and empirically re-scale base qualities at the end of the fragments.

Variants were called using the consensus caller of samtools/bcftools v1.2[34] limiting calls to the 9908 capture sites. Variant calls with a QUAL value lower than 25 were removed. The genotypes for historical and ancient samples were merged with previously published extant bovid 40k capture data[26], and *Bos primigenius* (aurochs) sample CPC98[35]. Only genotypes for the 9908 loci common among all data were retained.

<u>Supplementary Note 2:</u>

<u>DNA analyses</u>

Phylogenetic analysis

Mitochondrial control region phylogeny

The 60 newly sequenced bovid mitochondrial regions (Supplementary Data 1) were manually aligned, using SeaView v4.3.5[36]. These sequences were aligned with 302 published sequences (Supplementary Table 4) representing the following bovid mitochondrial lineages: European bison or wisent (*Bison bonasus*), American bison (*Bison bison*), steppe bison (*Bison priscus*), zebu (*Bos indicus*), and cattle (*Bos taurus*). Among these published sequences, 5 were from steppe bison collected in the Urals (Shapiro et al. 2004, Supplementary Data 1).

The TN93+G6 model of nucleotide substitution was selected by comparison of Bayesian information criterion (BIC) scores in ModelGenerator v0.85[37]. A phylogenetic tree was then inferred using both maximum-likelihood and Bayesian methods (Figure 2A). Bayesian analyses were performed using the program MrBayes v3.2.3[38]. Posterior estimates of parameters were obtained by Markov chain Monte Carlo sampling with samples drawn every 1000 steps. We used 2 runs, each of four Markov chains, comprising one cold and three heated chains, each of 10 million steps. The first 50% of samples were discarded as burn-in before the majority-rule consensus tree was calculated. A maximum-likelihood analysis was performed with the program PhyML v3[39], using both NNI and SPR rearrangements to search for the tree topology and using approximate likelihood-ratio tests to establish the statistical support of internal branches. Complete phylogenies inferred using both methods are shown in Supplementary Figure 4.

Whole mitochondrial genome phylogeny

The 16 newly sequenced bison whole mitochondrial genomes (Supplementary Data 1) were aligned with 31 published sequences (Supplementary Table 5) representing the following bovid mitochondrial lineages: 3 wisent (*Bison bonasus*), 8 American bison (*Bison bison*), 1 steppe bison (*Bison priscus*), 5 yaks (*Bos grunniens – Bos mutus*), 2 zebus (*Bos indicus*), 7 cattle (*Bos taurus*), 2 aurochsen (*Bos primigenius*), and 4 buffalo (*Bubalus bubalis*).

We used the same methods as described above for the control region to align and estimate the phylogeny. The HKY+G6 model of nucleotide substitution was selected through comparison of BIC scores (Figures 2B and S5).

Estimation of evolutionary timescale

To estimate the evolutionary timescale, we used the program BEAST v1.8.1[40] to conduct a Bayesian phylogenetic analysis of all radiocarbon-dated samples from CladeX and wisent (Figure 1C). The GMRF skyride model[41] was used to account for the complex population history, and a strict clock was assumed. We found support for a strict molecular clock based on replicate analyses using a relaxed uncorrelated lognormal clock[42], which could not reject the strict clock assumption.

Mean calibrated radiocarbon dates associated with the sequences were used as calibration points. Some samples appear to be older than 55 ky: one from the Urals, four from the North Sea and five from the Caucasus (Supplementary Data 1). Because

these dates have effectively infinite radiocarbon error margins, we allowed them to vary in the analysis by treating them as distinct parameters to be estimated in the model[43]. The dated samples from Mezmaiskaya Cave are from stratigraphic layers 2B4 and 2B3, which lie atop of layer 3. All these lower Middle Palaeolithic layers at Mezmaiskaya have 14C results beyond the radiocarbon limit, reflected in the predominance of greater-than or near-background limit ages[11], and therefore are consistent with the electron spin resonance (ESR) chronology for these levels[10], which suggests mean ages in the range from 53 to 73 ky BP (including error margins). Consequently, for each Caucasian sample, we specified a lognormal prior age distribution (mean=8,000) with an offset of 50 ky and with 95% of the prior probability less than 80 ky. A similar prior distribution (mean=26,000) was used for the five remaining samples that had infinite radiocarbon dates, with a 95% prior probability less than 150 ky. Based on the results of all four phylogenetic analyses described above, which showed strong support for the reciprocal monophyly of CladeX and wisent when outgroups were included, this monophyly was constrained for the BEAST runs.

All parameters showed sufficient sampling (indicated by effective sample sizes above 200) after 5,000,000 steps, with the first 10% of samples discarded as burn-in. In addition, a date-randomization test was conducted to check whether the temporal signal from the radiocarbon dates associated with the ancient sequences was sufficient to calibrate the analysis[44]. This test randomizes all dates and determines whether the 95% high posterior density (HPD) intervals of the rates estimated from the date-randomized data sets include the mean rate estimated from the original data set (Supplementary Figure 6).

The time to the most recent common ancestor (tMRCA) between wisent and CladeX mitochondrial lineages was estimated at 121.6 kyr (92.1 – 152.3) (Figure 2C). The tMRCAs for the two lineages was inferred to be 69.3 kyr (53.4 – 89.4) for wisent and 114.9 kyr (89.2 – 143.1) for CladeX. Furthermore, there is some phylogeographical structure within CladeX, with all individuals from the North Sea forming a basal group, which existed before the population replacement with steppe bison, but complete mixture of genetic diversity between all locations after re-colonization. In addition, the tMRCA of the MIS 3 diversity of CladeX was estimated to be about 53.1 kyr (41.5 – 67.5). This date closely matches the ages of the last observed MIS 4 CladeX individuals across all sampled locations, supporting the idea of a population movement and contraction of wisent individuals towards a refugium during the warmer period of MIS 3 in Europe.

Nuclear phylogeny from bovine SNP locus data

Phylogenetic trees were inferred from nuclear locus data (see next section for information about the data sets). First, a phylogenetic tree of modern representatives of bovid species, and with sheep as an outgroup, was inferred from published 40,843 data[26] (Supplementary Figure 7). Using RAxML v8.1.21[45], the three characters (genotype states AA, AB and BB) from the BovineSNP50 chip were considered as different states in an explicit analogue of the General Time Reversible (GTR) substitution model, with separate substitution parameters for the three possible transformations. For all analyses, 20 maximum likelihood searches were conducted to

find the best tree, and branch support was estimated with 500 bootstrap replicates using the rapid bootstrapping algorithm[46].

This species tree, estimated from genome-wide nuclear locus data, shows that the extant bison species (wisent and American bison) are sister taxa, contrary to the phylogenetic signal from the maternally inherited mitochondrial genome. This topology also clearly shows the paraphyletic status of the genus *Bos* (banteng, gaur, yak, zebu and cattle), as it also includes the genus *Bison* (wisent and American bison).

Using the same method, we reconstructed the phylogeny of bison with the inclusion of five pre-modern samples (for which the highest number of nuclear loci were called amongst the ~10k nuclear bovine SNPs). When only the two steppe bison specimens are included they form a sister-lineage to modern American bison (Supplementary Figure 8A). Similarly, when the steppe bison and pre-modern wisent (including ancient, historical and CladeX) are included, all five pre-modern specimens form a clade most closely related to American bison (Supplementary Figure 8C). However, when only the pre-modern wisent is included, the three specimens (ancient, historical and CladeX) form a clade that is most closely related to modern wisent (Supplementary Figure 8B). These conflicting results reflect the complex non-tree like relationships among the modern and pre-modern taxa, and are consistent with the hybridisation origin of wisent/CladeX and the severe bottleneck in the recent history of the wisent. Hence, we used population genomics statistics to study this nuclear locus dataset (see next section). Finally, these topologies are robust to the removal of transitions (see Supplementary Figure 8D), a minimum depth of 2 for variant calling, and haploidisation (data not shown).

Genome wide nuclear locus analysis

Captured nuclear loci corresponding to bovine SNPs for ancient samples were analysed with published genotypes from modern populations: 20 American bison were selected on the criterion that they do not display any detectable signal of recent introgression from cattle (unpublished data); 2 Yak (*Bos gruniens*); 10 water buffalo (*Bubalus bubalis*); and 10 Sheep (*Ovis aries*). Additionally, 7 modern wisent were selected (among 50 sequenced – [47]) as non-related individuals on a known five-generation pedigree (as shown in Supplementary Figure 9).

Principal Component Analysis

PCA (Figures 3A and S10) was performed using EIGENSOFT version 6.0.1 [48]. In Figure 3A, CladeX sample A006 was used as the representative of CladeX, as this sample contained the most complete set of nuclear loci called at the bovine SNP loci (see Supplementary Table 2). Other CladeX individuals, as well as ancient wisent, cluster towards coordinates 0.0, 0.0 (see Supplementary Figure 10), most likely due to missing data.

Topology testing with the D statistic

For three bison populations, assuming two bifurcations and no hybridisations, there are three possible phylogenetic topologies. For this simple case, the D statistic is expected to be significantly different from zero for exactly two of the three topologies, and not significantly different from zero for the most parsimonious topology. We therefore calculate a D statistic [49] for each of these three topologies, using the sheep (*Ovis aries*) as an outgroup.

When D statistics for the set of three topologies do not indicate zero for one topology and non-zero for the other two, the true phylogeny is not treelike. However, the most parsimonious topology may still be apparent when considering only small amounts of introgression from populations of similar size. The interpretation of a most parsimonious tree topology is not valid where confidence intervals around the D statistic closest to zero, contain one or more of the other D statistics.

In this manner, the D statistic was used to indicate the most parsimonious topology for phylogenies including CladeX, ancient wisent, historical wisent, modern wisent, steppe bison and aurochs (Supplementary Figure 11). D statistics were calculated using ADMIXTOOLS version 3.0, git~3065acc5 [50].

Following concern over the limited amount of data for CladeX, particularly in samples other than 6A, we calculated the D statistics with sample 6A omitted from the analysis (Supplementary Figure 12). The most parsimonious topologies match in both cases.

Sensitivity to other factors were also investigated, such as setting a bovine SNP site coverage depth threshold of two (Supplementary Figure 13), changing the outgroup to *Bubalus bubalis* (Asian water buffalo, Supplementary Figure 14), and haploidisation by randomly sampling an allele at heterozygous sites (Supplementary Figure 15). None of these factors had notable influences on the outcome.

We also considered that the obtained topologies may have been caused by the small number of observed loci. To determine how sensitive the topology testing was missing data, we performed bootstrap resampling of the locus calls on decreasingly sized subsets of the data (Supplementary Table 7). For 10,000 bootstraps, we counted how often we obtained a result other than shown in Supplementary Figure 11.

For this bootstrap, a topology is considered to be simple if: (1) It has a D statistic which, uniquely amongst the set of three, is not significantly different from zero, or (2) All three are significantly different from zero but one has a D statistic closest to zero, with confidence intervals that do not overlap the D statistic for the other two topologies.

For simple topologies, we counted how often the bootstrap replicate suggested a simple topology that did not match the most parsimonious topology in Supplementary Figure 11. For non-simple topologies, we counted how often the result suggested any simple topology. In both cases, a lack of support for any simple topology (such as multiple topologies having a D statistic not significantly different from zero) was not counted.

This bootstrapping shows that the D statistics are robust to the small number of observed genotypes.

Admixture proportion determination using an f4 ratio

The proportion of the wisent's ancestry differentially attributable to the steppe bison and the aurochs, was estimated with AdmixTools using an f4 ratio, as described in [50] with sheep (*Ovis aries*) as the outgroup. For the admixture graph shown in Supplementary Figure 16, the admixture proportion, α, is the ratio of two f4 statistics.

$$\alpha y = F4(A, O; X, C)$$

$$y = F4(A, O; B, C)$$

$$\alpha = \frac{\alpha y}{y} = \frac{F4(A, O; X, C)}{F4(A, O; B, C)}$$

For the estimation of admixture proportions using an f4 ratio, it is intended that the ingroup A, while closely related to B, has diverged from B prior to the admixture event. However, in the context of steppe ancestry for wisent, no such population matching ingroup A was available. The admixture graph for wisent is shown in Supplementary Figure 17.

$$\alpha y = F_4(AmericanBison, O; Wisent, Aurochs)$$

$$x + y = F_4(AmericanBison, O; Steppe, Aurochs)$$

$$\alpha \approx \frac{\alpha y}{x + y} = \frac{F_4(AmericanBison, O; Wisent, Aurochs)}{F_4(AmericanBison, O; Steppe, Aurochs)}$$

Where α in Supplementary Figure 17 is approximately determined by the f4 ratio for small branch lengths x. The f4 ratio we calculate therefore represents a lower bound on the proportion of steppe bison present in the wisent populations. The steppe ancestry was found to be at least 0.891, with a standard error of 0.026 (Supplementary Table 6-A).

Sensitivity to haploidisation was checked by randomly sampling an allele at heterozygous sites (Supplementary Table 6-B), which had no notable influence on the outcome.

Hypergeometric test for shared derived alleles

To test whether the wisent lineages (including CladeX) have a common hybrid ancestry (Supplementary Figure 18A), or whether multiple independent hybridisation events gave rise to distinct wisent lineages (Supplementary Figure 18B), we identify nuclear loci which have an ancestral state in the aurochs lineage, but a derived state in the steppe lineage (see next section 'identification of derived alleles'). Under the assumption of a single hybrid origin, we expect a common subset of derived steppe alleles to be present in the various wisent lineages. In contrast, multiple hybridisation events would result in different subsets of derived steppe alleles being present in different wisent lineages. Likewise, we expect the subset of derived aurochs alleles to indicate either one, or multiple hybridisation events.

If the total number of derived steppe alleles is s, the number of derived steppe alleles observed in one wisent lineage is a, and the number in a second wisent lineage is b, then under model B, the number of sites which are found to be in common is a random variable X~HGeom(a, s-a, b). Where HGeom is the hypergeometric

distribution, having probability mass function:

$$P(X = k) = \frac{\binom{a}{k}\binom{s-a}{b-k}}{\binom{s}{b}}$$

For the number of derived steppe alleles in common between two wisent lineages, c, we calculate P(X ≥ c). This indicates the likelihood of having observed c or more derived steppe alleles in common, if independent hybridisation events gave rise to both wisent and CladeX lineages.

Likelihoods were calculated for steppe derived alleles on all pairwise combinations of wisent lineages (Supplementary Table 8), and then repeated for derived aurochs alleles (Supplementary Table 9). This provides strong support for an ancestral hybridisation event occurring prior to the divergence of the wisent lineages.

We note that parallel genetic drift may also result in a pattern of alleles observed to be derived in the steppe lineage and the wisent lineages, however this is only a confounding factor where the parallel drift occurred in the post hybridisation lineage common to wisent and CladeX in Supplementary Figure 18A. Therefore, this only confounds the determination of genomic positions from a specific parent population, not that the wisent and CladeX lineages have shared ancestry post hybridisation. Alleles under strong selection following distinct hybridisation events would also be shared between lineages more often than if they were randomly distributed. We consider this situation unlikely, as it would require that the same alleles were randomly introgressed repeatedly, and then a strong selective advantage of the alleles at all times and in all environments.

Although we cannot reject the hypothesis that the modern European bison morph may be recent, and only appeared after the LGM as an adaptation to the Holocene environment in Europe, it would mean that the *Bos* mitochondrial lineage has been maintained in the steppe bison diversity throughout the late Pleistocene, and that only individuals carrying this mitochondrial lineage survived in Europe. Therefore, a hybrid origin of the European morph prior to 120 kyr, and maintained during the late Pleistocene, is more parsimonious with the current data.

Identification of derived alleles

The identification of a derived allele in the B lineage of Supplementary Figure 16, for the above analysis, can be performed in a simple way. If the ancestral allele is fixed in both C and the outgroup O, and the derived allele is fixed within B, then the site may be readily identified as derived. However, such fixed alleles are likely to be rare, especially in large populations, and therefore in limited number in our 10K SNP subset. Furthermore, a steppe bison derived allele observed in a wisent population may not be fixed in the wisent, as the population may also contain the ancestral allele from the aurochs lineage.

Relaxing the criterion of allele fixation in any lineage, we identify differential ancestry using the difference in allele frequencies between populations. An ancestral site is one in which the allele frequency closely matches that of the outgroup and a derived site has an allele frequency differing from the outgroup.

For the admixture graph in Supplementary Figure 16, where population X has ancestry from both B and C lineages, with outgroup O, we define an allele frequency shift in B, analogous to a derived state, if

$\hat{F}_2(C,O) < \hat{F}_2(X,C)$ and $\hat{F}_2(C,O) < \hat{F}_2(X,O)$,

where $\hat{F}_2(M,N)$is an unbiased estimate of $(m-n)^2$, for populations M and N with population allele frequencies *m* and *n* at a single locus, as in Appendix A of [50]. Similarly, we define the allele frequency shift in B to have the same shift in X if, in addition to the shift in B:

$\hat{F}_2(B,X) < \hat{F}_2(B,C)$ and $\hat{F}_2(B,X) < \hat{F}_2(B,O)$ and

$\hat{F}_2(B,X) < \hat{F}_2(X,C)$ and $\hat{F}_2(B,X) < \hat{F}_2(X,O)$ and

$\hat{F}_2(C,O) < \hat{F}_2(B,C)$ and $\hat{F}_2(C,O) < \hat{F}_2(B,O)$.

By observing a shared allele frequency shift instead of shared fixed alleles, we obtain greater sensitivity to the phylogenetic signal that is specific to one ancestral lineage. As for fixed derived alleles, the specific sites showing an allele frequency shift are identified, and can then be compared between multiple daughter populations.

Admixture proportion determination using ABC and simulated data

As the f4 ratio test is giving an upper limit to the amount of aurochs introgression (due to the branch length uncertainty shown in Supplementary Figure 17), we independently test the admixture proportions using simulated data and an ABC approach.

Approximate Bayesian Computation (ABC) is a likelihood-free methodology employed when calculating likelihood functions is either impossible or computationally expensive[51]. The methodology relies on being able to efficiently simulate data, and then compare simulated data to observed data. When simulated data is sufficiently close to the observed data, the parameters used to simulate the data are retained in a posterior distribution.

Consider a single locus, which for three individuals A, B, and C, two different genotypes are observed. The three possible patterns that can be observed are AB, BC, and AC, denoted by the tree tips with shared state. The observed pattern results from a single mutation somewhere on the gene tree, where the position of the mutation relative to the internal node defines which pattern is observed. For example, from the un-rooted gene tree in Supplementary Figure 19c, if a mutation occurs on the branch between C and the internal node, the pattern AB is observed. We assume the relevant time scales are short enough that multiple mutations at a single locus are rare (infinite sites model[52]).

Under the assumption of neutral and independent mutations, the number of fixed mutations accumulating on a branch is Poisson distributed with mean $\mu \times t$, where μ is mutations per locus per generation, and time t is in units of $2N_e$ generations[53,54]. The counts $\boldsymbol{n} = (n_{ab}, n_{bc}, n_{ac})$, of observed site patterns AB, BC, and AC, are random variables, which for topology X_1 (Supplementary Figure 19c),

$$n_{ab} \sim Pois(T_m + T_c),$$

$$n_{bc} \sim Pois(T_a),$$

$$n_{ac} \sim Pois(T_b),$$

and topology X_2 (Supplementary Figure 19d),

$$n_{ab} \sim Pois(T_c),$$
$$n_{bc} \sim Pois(T_m + T_a),$$
$$n_{ac} \sim Pois(T_b),$$

where $\boldsymbol{T} = (T_a, T_b, T_c, T_m)$ are branch lengths in units of evolutionary time of $2N_e\mu$ generations, and the total number of observed patterns is $N = n_{ab} + n_{bc} + n_{ac}$. Thus for a locus where two genotypes are observed, the probability of patterns AB, BC, AC, is given by $\boldsymbol{p}^{\boldsymbol{T}} = (p_{ab}^{\boldsymbol{T}}, p_{bc}^{\boldsymbol{T}}, p_{ac}^{\boldsymbol{T}}.)$, where for topology X_1 (Supplementary Figure 19c),

$$\begin{aligned} P(\mathrm{AB}|\boldsymbol{T}, X_1) &= p_{ab}^{\boldsymbol{T},X_1} = (T_m + T_c)/(T_m + T_c + T_a + T_b) \\ P(\mathrm{BC}|\boldsymbol{T}, X_1) &= p_{bc}^{\boldsymbol{T},X_1} = T_a/(T_m + T_c + T_a + T_b) \\ P(\mathrm{AC}|\boldsymbol{T}, X_1) &= p_{ac}^{\boldsymbol{T},X_1} = T_b/(T_m + T_c + T_a + T_b) \end{aligned}$$

and for topology X_2 (Supplementary Figure 19d),

$$\begin{aligned} P(\mathrm{AB}|\boldsymbol{T}, X_2) &= p_{ab}^{\boldsymbol{T},X_2} = T_c/(T_m + T_c + T_a + T_b) \\ P(\mathrm{BC}|\boldsymbol{T}, X_2) &= p_{bc}^{\boldsymbol{T},X_2} = (T_a + T_m)/(T_m + T_c + T_a + T_b) \\ P(\mathrm{AC}|\boldsymbol{T}, X_2) &= p_{ac}^{\boldsymbol{T},X_2} = T_b/(T_m + T_c + T_a + T_b). \end{aligned}$$

We simulate site pattern counts for each of the two species trees in Supplementary Figure 19 by drawing from a Multinomial distribution, where for tree topology X_1, $\boldsymbol{n}^{X_1} \sim \mathrm{Mult}(N, \boldsymbol{p}^{\boldsymbol{T},X_1})$, and for tree topology X_2, $\boldsymbol{n}^{X_2} \sim \mathrm{Mult}(N, \boldsymbol{p}^{\boldsymbol{T},X_2})$.

Given a collection of site pattern counts from a hybrid tree with hybridisation parameter $\gamma \in [0,1]$ (Figure S19e), we expect that the combined site pattern counts will be a linear combination of the counts for the different topologies X_1 and X_2. This assumption is reasonable for a large number of total observations N. The simulated counts, $\boldsymbol{n}^{\gamma}$, of site patterns for the hybridised tree is then given by

$$\begin{aligned} \boldsymbol{n}^{\gamma} &= \gamma \boldsymbol{n}^{X_1} + (1-\gamma)\boldsymbol{n}^{X_2} \\ &= (n_{ab}^{\gamma}, n_{bc}^{\gamma}, n_{ac}^{\gamma}). \end{aligned}$$

As branch lengths are not known (μ, N_e and number of generations are all unknown), we use uninformative priors for the branch lengths. Furthermore, we only require relative branch lengths, so branch lengths $\boldsymbol{T}$ used for simulation were scaled such that $T_b = 1$. Hence we can meaningfully simulate counts of site patterns $\boldsymbol{n}^{\gamma}$ under hybridisation, for comparison to observed site pattern counts.

We perform ABC using the R package 'abc', with a ridge regression correction for comparison of the simulated and observed data using the "abc" function[55]. The distance between the observed and simulated data sets is calculated as the Euclidean distance in three-dimensional space. A tolerance $\epsilon = 0.005$ was chosen so that the closest $\ell \times \epsilon$ simulated data sets are retained. For each analysis we had $\ell = 100000$, resulting in 500 posterior samples.

We performed leave-one-out cross-validation using the function "cv4abc" on $\ell' = 250$ randomly selected simulations, and report the prediction error, calculated as

$$E_{\text{pred}} = \frac{\sum_{i=1}^{\ell'} (\hat{\gamma}_i - \gamma_i)^2}{\text{Var}(\gamma_i)}$$

for each analysis. At most the prediction error was 0.5111 standard deviations away from zero, and so we observe that the ridge regression has performed well (see Supplementary Table 11).

Similarly, on inspection of the cross-validation plots, we observe that the ridge regression performs well for γ, as the true simulated values of γ are well estimated by the ridge regression correction. Hence the correction has strengthened the parameter inference methodology when compared to a simple rejection algorithm.

We avoid reporting sample means due to the heavy negative skew in the posterior distributions of γ, and hence report the median (the most central ordered observed value) and mode of each distribution. The mode is estimated using a kernel density estimate of the posterior distribution. Not all simulated data is equally ‘close’ to the observed data, and the median and mode are weighted according to these distances[56].

The weighted posterior median was between 0.8250 and 0.8660, and the weighted posterior mode was between 0.9034 and 0.9384. These measures of centre indicate evidence for some non-zero level of hybridisation from the Aurochs genome. Evidence against hybridsation must be indicated by overwhelming support for either $\gamma = 0$ or $\gamma = 1$ (no mixing of the tree topologies). However, these values lie on either end of the support for the prior distribution of γ, and hence any resulting posterior distribution for γ. There- fore, classical highest probability density (HPD) intervals cannot be used to indicate uncertainty in the estimates of these measures of centre, as any interval of density less than 100% will result in zero and one being artificially omitted by construction. This is not evidence for or against hybridisation, but rather a consequence of the way in which we calculate HPD intervals.

Supplementary Table 11 gives empirical posterior probabilities for different levels of hybridisation. For example, the first column gives the empirical posterior probability of observing at least 1% hybridisation. This is found for each trio by calculating the total proportion of posterior samples where $0.01 \leq \gamma \leq 0.99$. In general, for some percentage of hybridisation α, Supplementary Table 11 reports

$$[P(\frac{\alpha}{100} \leq \gamma \leq 1 - \frac{\alpha}{100})]$$

for α = 1%, 2%, 3%, 4% and 5%, from the posterior distribution of γ.

As there is no accepted value of γ for which we can claim that significant hybridisation has occurred, we leave it to the reader to consider what they consider to be a significant level of hybridisation, and to find the appropriate probability. However, if one considers 1% hybridisation to be significant, then the observed data indicates that the data has between a 95.80% and 97.20% chance of being from a hybridised topology. Similarly, if one considers 5% hybridisation to be significant, then the observed data has between a 76.40% and 85.00% chance of being from a hybridised topology.

Asymmetrical hybridisation

In this study, we show that wisent and CladeX are of hybrid origin, certainly between ancient aurochs and steppe bison forms. This is consistent with the population structure of most bovids, where a single bull usually breeds with different females of multiple generations. As explained in[57], this usually results in asymmetrical hybridization when males of one species (steppe bison here) dominate males of the other species (aurochs here), therefore preferentially mating with female aurochs, as well as their offspring, potentially over several generations. In addition, male F_1 hybrids are usually sterile or sub-fertile, increasing the amount of steppe bison genomic contribution to the offspring. As illustrated in Supplementary Figure 20, after just a few generations, this mating process results in individuals that are essentially steppe bison for their nuclear genome, but with an aurochs mitochondrial genome (strictly maternally inherited), which is the result that we obtained from the genotyping of historical and ancient wisent individuals (including CladeX).

Supplementary Note 3:

Paleoenvironment reconstruction and stable isotope analyses in the Ural region

The Urals are a well sampled region, with the highest number of genotyped bones through time (Figure 5 and S22). We generated a convex hull based on geo-referenced site locations for all genotyped ancient samples collected from the Urals (Supplementary Figure 21). We used the HadCM3 global circulation model and BIOME4 model to reconstruct paleoclimate and environmental conditions for the Ural region throughout the period from 70,000 years ago to the present day.

We used the HadCM3 global circulation model to reconstructed paleoclimate proxies for the Ural region. The HadCM3 consists of linked atmospheric, ocean and sea ice models at a spatial resolution of 2.5° latitude and 3.75° longitude, resampled at a 1° x 1° latitude/longitude grid cell resolution [58]. The temporal resolution of the raw data is 1,000 year slices back to 22,000BP and 2,000 year slices from 22,000 to 80,000BP [58] We used these palaeo-climate simulations to derive estimates of annual mean daily temperature and Köppen-Geiger climate classifications [59] throughout the period from 70,000 years ago to the present day. We intersected each grid cell in the Ural study region (n = 51) with the derived climate estimates, at each point in time, using ArcGIS 10. We calculated the mean temperature for the region and change in the proportion of the study region represented by four Köppen climate classes, each differing temperature: Dfa (hot summers), Dfb (warm summers), Dfc (cool summers), Dfd (continental temperatures). These are shown in Supplementary Figure 22. Interestingly, our reconstructions for the Urals show a decrease in area with hot and warm summer conditions (Dfa and Dfb) after 35kya.

BIOME4 was used to infer paleovegetation types. BIOME4 is a coupled biogeographical and biogeochemical model that simulates the distribution of 28 plant functional types (PFT) at a global scale [60]. Model inputs for each grid cell are monthly climate (mean annual temperature, mean annual precipitation and mean annual sunshine hours), atmospheric [CO_2], and soil texture class. Ecophysiological constraints determine which PFT is likely to occur in each grid cell. A coupled carbon and water flux model calculates the leaf area index that maximizes net primary production (in gC m^{-2} $year^{-1}$) for each PFT. Competition between PFTs was simulated by using the optimal net primary production of each PFT as an index of competitiveness. Global maps of BIOME4 PFTs were accessed at the same spatial and temporal resolution as the paleoclimate data (http://www.bridge.bris.ac.uk/resources/simulations/). We grouped PFTs into three categories: Grassland (PFT identify numbers = 18-20); Tundra (ID = 22-26); and Forest (ID = 7-11). For each grid cell in the Ural study region, at each point in time, we determined whether the dominant PFT was grassland, tundra or forest. Interestingly the vegetation shift between an all forest-like landscape to a landscape represented by a large proportion of tundra and grassland-like vegetation occurred after 35kya, which coincides with a decrease in hot and warm summer conditions (see above).

These results from the paleovegetation and climate inferences agree with previous landscape reconstructions of the region: In the Middle Urals, where almost all the samplings sites were located, the areas covered with arboreal vegetation underwent

changes during MIS3. Spruce and birch open forests were widespread during coolings, and spruce and birch forest-steppe with occurrence of pine formed during warmings. Mesophilic meadows dominated by forbs and grasses were also prevalent during warm climatic events (Lapteva, 2008; 2009; Pisareva and Faustova, 2008). In the south, where one of the sites (Gofmana) is situated, steppe landscapes dominated by Asteraceae, Artemisia, and Poaceae were widespread. Spruce, birch and pine forests covered the areas along the rivers (Smirnov, Bolshakov, Kosintsev et al., 1990). The following was reconstructed for the territory of the Irtysh River: forest-steppe landscapes with pine (Pinus s/g Haploxylon) and spruce forests, as well as meadows with a predominance of Cyperaceae and Poaceae and small quantities of Artemisia and Chenopodiaceae (Araslanov *et al*. 2009).

During MIS2, periglacial forest-steppes dominated by herbaceous communities were typical of the Last Glacial Maximum. Larch, pine and birch covered the river-valleys. Herbaceous vegetation was dominated by goosefoot, sagebrush and grass (Grichuk 2002). Periglacial forest-steppes with arboreal vegetation, including pine-birch forests and small quantities of spruce have been reconstructed for the Last Glacial Termination. Areas covered with sagebrush-goosefoot steppes with small quantities of grass were widespread (Lapteva, 2007).

At later stages of MIS2, periglacial forb-grass forest-steppes with pine, birch and small quantities of spruce have been reconstructed for the Sur'ya 5 and Rasik 1 sites [61]. Periglacial steppes dominated by Artemisia, Rosaceae, Chenopodiaceae, Cichorioideae and Poaceae have been reconstructed for the Voronovka site. Pinus sylvestris and Betula pubescens with occurrence of spruce (Picea), oak (Quercus) and teil (Tilia) covered the river-valleys [62].

The palynological analyses and landscape reconstruction suggest that both bison forms inhabited semi-open landscapes of forest-steppe type, where arboreal vegetation was represented by birch, spruce, pine and sometimes larch, while steppe and meadow herbaceous communities were observed. However, only CladeX (specifically from the Gofmana site, during MIS 3, Rasik 1 and Sur'ya 5, and Voronovka sites, during MIS2) also inhabited steppe-like landscapes, showing a more diverse ecological niche than steppe in this region.

In addition to the paleo-climate and -vegetation reconstructions, stable isotope values (δ13C and δ15N) obtained for all the genotyped bison individuals from the Ural region were compared between steppe bison and wisent (Supplementary Figure 23). Wisent individuals displayed more diverse stable isotope ratios than the steppe bison individuals. This observation is consistent with feeding in more diverse vegetations communities, which correlates well with the reconstructed paleo-environments for the region in the time periods they are found.

Modelled paleo-climate and -vegetation reconstruction at the sampling locations in the southern Urals suggest drastic shifts, which coincide in time with the observed population replacements between steppe bison and wisent. More specifically, between 14 and 31 kya wisent were likely to exist in environmental condition characterised by relatively cold average temperatures, open landscapes with tundra-like flora, and the absence of warm summers. Although modern wisent are found today in wood-like habitats, it has been suggested that they are living in sub-optimal habitat, and paleodiet reconstructions have placed ancient wisent in tundra-like environments, in agreement with our observations [63].

Interestingly, the steppe bison was only recorded when forest vegetation was inferred to dominate the landscape, adding to the evidence that this form of bison might not have been exclusively steppe-adapted [63,64].

Supplementary Note 4:

Cave painting

The present survey, placing wisent across Europe (from the Urals/Caucasus to Ukraine/Italy) during MIS2 and late MIS3, suggests that depictions of bison in European Palaeolithic art, such as cave painting, carving and sculptures, are likely to include representations of wisent. Paleolithic art representations have often been used to infer the morphological appearance of steppe bison, sometimes in great detail [64,4,65–67]. And until now, the steppe bison (i.e., direct ancestor of modern American bison) has always been assumed to be the unique model present at the time of cave painting, and therefore, the diversity within the representations of bison was mainly explained by putative cultural and individual variations of style through time [68–70]. However, in the vast diversity of bison representations (820 pictures representing 20.6% of all known cave ornamentation, according to [71]), two consistent morphological types can be distinguished (see Fig 1 and Fig S24-27). The first type, abundant prior to the last glacial maximum, is characterized by long horns (with one curve), a very oblique dorsal line and a very robust front part of the body (solid shoulders versus hindquarters), all these traits being similar to the modern American bison. The second type, dominating the more recent paintings between 18 and 15 kya, displays thinner sinuous horns (often with double curve), a smaller hump and more balanced dimensions between the front and the rear of the body, similar to the modern wisent lineage, and to some extant the *Bos* lineage. The imposing figure of the steppe bison, with its high hump and long horns stepping out the head profile, certainly was a very strong influence on the artists painting in the cave in Europe before the last glacial maximum. However, later generations thoroughly depicted the slender shape of the more recent form of bison. Considering the geographical and temporal distribution of genotyped steppe bison and wisent presented here, particularly the ~16,000 years old wisent B individual from Northern Italy, it is likely that the variety of bison representations in Paleolithic art does not just come from stylistic evolution, but actually represents different forms of bison (i.e., pre and post-hybridisation) through time.

Supplementary References

1. Wolff, E. W., Chappellaz, J., Blunier, T., Rasmussen, S. O. & Svensson, A. Millennial-scale variability during the last glacial: The ice core record. *Quaternary Science Reviews* **29,** 2828–2838 (2010).
2. Shapiro, B. *et al.* Rise and Fall of the Beringian Steppe Bison. *Science* **306,** 1561–1565 (2004).
3. Leroi-Gourhan, A. & Allain, J. *Lascaux inconnu*. (CNRS, 1979).
4. Capitan, L., Breuil, H. & Peyrony, D. *La caverne de Font-de-Gaume, aux Eyzies (Dordogne)*. (Imprimerie du Chêne, 1910).
5. Lorblanchet, M. *La grotte ornée de Pergouset (Saint-Géry, Lot). Un sanctuaire secret paléolithique*. (Maison des Sciences de l'Homme, 2001).
6. Barrière, C. L'art pariétal de Rouffignac, la grotte aux cent mammouths. *Bulletins et Mémoires de la Société d'anthropologie de Paris* **10,** 144–145 (1983).
7. Groves, C. & Grubb, P. *Ungulate Taxonomy*. (Johns Hopkins University Press, 2011).
8. Drees, M. & Post, K. Bison bonasus from the North Sea, the Netherlands. *Cranium* **24,** 48–52 (2007).
9. Llamas, B. *et al.* High-Resolution Analysis of Cytosine Methylation in Ancient DNA. *PLoS ONE* **7,** e30226 (2012).
10. Skinner, A. R. *et al.* ESR dating at Mezmaiskaya Cave, Russia. *Applied Radiation and Isotopes* **62,** 219–224 (2005).
11. Pinhasi, R., Higham, T. F. G., Golovanova, L. V. & Doronichev, V. B. Revised age of late Neanderthal occupation and the end of the Middle Paleolithic in the northern Caucasus. *PNAS* **108,** 8611–8616 (2011).

12. Reimer, P. J. *et al.* IntCal13 and Marine13 Radiocarbon Age Calibration Curves 0–50,000 Years cal BP. *Radiocarbon* **55,** 1869–1887 (2013).
13. Willerslev, E. & Cooper, A. Ancient DNA. *Proc Biol Sci* **272,** 3–16 (2005).
14. Brotherton, P. *et al.* Neolithic mitochondrial haplogroup H genomes and the genetic origins of Europeans. *Nat Commun* **4,** 1764 (2013).
15. Rohland, N. & Hofreiter, M. Ancient DNA extraction from bones and teeth. *Nat. Protocols* **2,** 1756–1762 (2007).
16. Meyer, M. & Kircher, M. Illumina Sequencing Library Preparation for Highly Multiplexed Target Capture and Sequencing. *Cold Spring Harb Protoc* **2010,** pdb.prot5448 (2010).
17. Kircher, M., Sawyer, S. & Meyer, M. Double indexing overcomes inaccuracies in multiplex sequencing on the Illumina platform. *Nucl. Acids Res.* **40,** e3–e3 (2012).
18. Cone, R. W. & Schlaepfer, E. Improved In Situ Hybridization to HIV with RNA Probes Derived from PCR Products. *J Histochem Cytochem* **45,** 721–727 (1997).
19. Liu, C., Bernstein, B. & Schreiber, S. *DNA linear amplification*. (Scion Publishin Ltd, 2005).
20. Rohland, N. & Reich, D. Cost-effective, high-throughput DNA sequencing libraries for multiplexed target capture. *Genome Res.* gr.128124.111 (2012). doi:10.1101/gr.128124.111
21. Konietzko, U. & Kuhl, D. A subtractive hybridisation method for the enrichment of moderately induced sequences. *Nucleic Acids Res.* **26,** 1359–1361 (1998).
22. Rohland, N., Harney, E., Mallick, S., Nordenfelt, S. & Reich, D. Partial uracil–DNA–glycosylase treatment for screening of ancient DNA. *Philosophical Transactions of the Royal Society of London B: Biological Sciences* **22,** 939–949 (2015).

23. Haak, W. *et al.* Massive migration from the steppe was a source for Indo-European languages in Europe. *Nature* **522,** 207–211 (2015).
24. Maricic, T., Whitten, M. & Pääbo, S. Multiplexed DNA Sequence Capture of Mitochondrial Genomes Using PCR Products. *PLoS ONE* **5,** e14004 (2010).
25. Untergasser, A. *et al.* Primer3Plus, an enhanced web interface to Primer3. *Nucl. Acids Res.* **35,** W71–W74 (2007).
26. Decker, J. E. *et al.* Resolving the evolution of extant and extinct ruminants with high-throughput phylogenomics. *PNAS* **106,** 18644–18649 (2009).
27. Matukumalli, L. K. *et al.* Development and Characterization of a High Density SNP Genotyping Assay for Cattle. *PLoS ONE* **4,** e5350 (2009).
28. Shankaranarayanan, P. *et al.* Single-tube linear DNA amplification (LinDA) for robust ChIP-seq. *Nat Meth* **8,** 565–567 (2011).
29. Schubert, M. *et al.* Characterization of ancient and modern genomes by SNP detection and phylogenomic and metagenomic analysis using PALEOMIX. *Nat. Protocols* **9,** 1056–1082 (2014).
30. Lindgreen, S. AdapterRemoval: Easy Cleaning of Next Generation Sequencing Reads. *BMC Research Notes* **5,** 337 (2012).
31. Li, H. & Durbin, R. Fast and accurate short read alignment with Burrows–Wheeler transform. *Bioinformatics* **25,** 1754–1760 (2009).
32. Jónsson, H., Ginolhac, A., Schubert, M., Johnson, P. L. F. & Orlando, L. mapDamage2.0: fast approximate Bayesian estimates of ancient DNA damage parameters. *Bioinformatics* **29,** 1682–1684 (2013).
33. Zimin, A. V. *et al.* A whole-genome assembly of the domestic cow, Bos taurus. *Genome Biology* **10,** R42 (2009).

34. Li, H. A statistical framework for SNP calling, mutation discovery, association mapping and population genetical parameter estimation from sequencing data. *Bioinformatics* **27,** 2987–2993 (2011).

35. Park, S. D. E. *et al.* Genome sequencing of the extinct Eurasian wild aurochs, Bos primigenius, illuminates the phylogeography and evolution of cattle. *Genome Biology* **16,** 234 (2015).

36. Gouy, M., Guindon, S. & Gascuel, O. SeaView Version 4: A Multiplatform Graphical User Interface for Sequence Alignment and Phylogenetic Tree Building. *Mol Biol Evol* **27,** 221–224 (2010).

37. Keane, T. M., Creevey, C. J., Pentony, M. M., Naughton, T. J. & Mclnerney, J. O. Assessment of methods for amino acid matrix selection and their use on empirical data shows that ad hoc assumptions for choice of matrix are not justified. *BMC Evolutionary Biology* **6,** 29 (2006).

38. Ronquist, F. *et al.* MrBayes 3.2: Efficient Bayesian Phylogenetic Inference and Model Choice Across a Large Model Space. *Syst Biol* **61,** 539–542 (2012).

39. Guindon, S. *et al.* New Algorithms and Methods to Estimate Maximum-Likelihood Phylogenies: Assessing the Performance of PhyML 3.0. *Syst Biol* **59,** 307–321 (2010).

40. Drummond, A. J. & Rambaut, A. BEAST: Bayesian evolutionary analysis by sampling trees. *BMC Evolutionary Biology* **7,** 214 (2007).

41. Minin, V. N., Bloomquist, E. W. & Suchard, M. A. Smooth Skyride through a Rough Skyline: Bayesian Coalescent-Based Inference of Population Dynamics. *Mol Biol Evol* **25,** 1459–1471 (2008).

42. Drummond, A. J., Ho, S. Y. W., Phillips, M. J. & Rambaut, A. Relaxed Phylogenetics and Dating with Confidence. *PLoS Biol* **4,** e88 (2006).

43. Shapiro, B. *et al.* A Bayesian Phylogenetic Method to Estimate Unknown Sequence Ages. *Mol Biol Evol* **28,** 879–887 (2011).

44. Ho, S. Y. W. *et al.* Bayesian Estimation of Substitution Rates from Ancient DNA Sequences with Low Information Content. *Syst Biol* **60,** 366–375 (2011).

45. Stamatakis, A. RAxML-VI-HPC: maximum likelihood-based phylogenetic analyses with thousands of taxa and mixed models. *Bioinformatics* **22,** 2688–2690 (2006).

46. Stamatakis, A., Hoover, P. & Rougemont, J. A Rapid Bootstrap Algorithm for the RAxML Web Servers. *Syst Biol* **57,** 758–771 (2008).

47. Pertoldi, C. *et al.* Phylogenetic relationships among the European and American bison and seven cattle breeds reconstructed using the BovineSNP50 Illumina Genotyping BeadChip. *Acta Theriol* **55,** 97–108 (2010).

48. Patterson, N., Price, A. L. & Reich, D. Population Structure and Eigenanalysis. *PLoS Genet* **2,** e190 (2006).

49. Durand, E. Y., Patterson, N., Reich, D. & Slatkin, M. Testing for Ancient Admixture between Closely Related Populations. *Mol Biol Evol* **28,** 2239–2252 (2011).

50. Patterson, N. *et al.* Ancient Admixture in Human History. *Genetics* **192,** 1065–1093 (2012).

51. Beaumont, M. A., Zhang, W. & Balding, D. J. Approximate Bayesian Computation in Population Genetics. *Genetics* **162,** 2025–2035 (2002).

52. Kimura, M. The Number of Heterozygous Nucleotide Sites Maintained in a Finite Population Due to Steady Flux of Mutations. *Genetics* **61,** 893–903 (1969).

53. Watterson, G. A. On the number of segregating sites in genetical models without recombination. *Theor Popul Biol* **7,** 256–276 (1975).

54. Hudson, R. in *Oxford Surveys in Evolutionary Biology* **7,** 1–44 (Oxford University Press, 1990).
55. Csilléry, K., François, O. & Blum, M. G. B. abc: an R package for approximate Bayesian computation (ABC). *Methods in Ecology and Evolution* **3,** 475–479 (2012).
56. Blum, M. G. B. & François, O. Non-linear regression models for Approximate Bayesian Computation. *Stat Comput* **20,** 63–73 (2009).
57. Groves, C. Current taxonomy and diversity of crown ruminants above the species level. *Zitteliana* **B 32,** 5–14 (2014).
58. Singarayer, J. S. & Valdes, P. J. High-latitude climate sensitivity to ice-sheet forcing over the last 120 kyr. *Quaternary Science Reviews* **29,** 43–55 (2010).
59. Peel, M. C., Finlayson, B. L. & McMahon, T. A. Updated world map of the Köppen-Geiger climate classification. *Hydrol. Earth Syst. Sci.* **11,** 1633–1644 (2007).
60. Kaplan, J. O. Geophysical Applications of Vegetation Modeling. (Lund University, 2001).
61. Lapteva, E. G. Landscape-climatic changes on the eastern macroslope of the Northern Urals over the past 50000 years. *Russ J Ecol* **40,** 267–273 (2009).
62. Lapteva, E. G. & Korona, O. M. Holocene vegetation changes and anthropogenic influence in the forest-steppe zone of the Southern Trans-Urals based on pollen and plant macrofossil records from the Sukharysh cave. *Veget Hist Archaeobot* **21,** 321–336 (2011).
63. Bocherens, H., Hofman-Kamińska, E., Drucker, D. G., Schmölcke, U. & Kowalczyk, R. European Bison as a Refugee Species? Evidence from Isotopic

Data on Early Holocene Bison and Other Large Herbivores in Northern Europe. *PLoS ONE* **10,** e0115090 (2015).

64. Guthrie, R. D. *Frozen fauna of the Mammoth Steppe : the story of Blue Babe*. (University of Chicago Press, 1990).
65. Bandi, H.-G. ; H., W. ;. Sauter, M. R. ;. Sitter, B. *La Contribution de la Zoologie et de L'Ethologie a L'Interpretation de L'Art des Peuples Chasseurs Prehistoriques*. (Editions Universitaires, 1984).
66. Guthrie, R. D. *The nature of Paleolithic art*. (University of Chicago Press, 2005).
67. Paillet, P. *Le bison dans les arts magdaléniens du Périgord*. (CNRS éd, 1999).
68. Breuil, H. *Quatre cents siècles d'art pariétal; les cavernes ornées de l'âge du renne.* (Centre d'études et de documentation préhistoriques, 1952).
69. Leroi-Gourhan, A. *Préhistoire de l'art occidental*. (1965).
70. Petrognani, S. *De Chauvet à Lascaux: l'art des cavernes, reflet de sociétés préhistoriques en mutation*. (Editions Errance, 2013).
71. Sauvet, G. & Wlodarczyk. L'art pariétal, miroir des sociétés paléolithiques. *Zephyrus: Revista de prehistoria y arqueología* **53,** 217–240 (2000).

References in Russian:

Arslanov KH, Laukhin SA, Maksimov FE, *et al*. (2009) Radiocarbon Chronology and Landscapes of Western Siberian Lipovsk-Novoselovsky Interstadial (on evidence of study section near V. Lipovka) // Fundamental Problems of Quaternary: Resultats and Trends of Further Researches. (Ed. A.E. Kantorovich). Novosibirsk. P. 44 – 47. (in Russian).

Grichuk VP (2002) Vegetation of the Late Pleistocene. In: A.A.Velichko (ed.), Dynamics of terrestrial landscape components and inner marine basins of Northern Eurasia during the last 130 000 years. Moscow: GEOS Publishers, pp. 64-88. (in Russian).

Lapteva EG (2007) Реконструкция ландшафтно-климатических изменений на территории Среднего Зауралья в позднеледниковье и голоцене на основе палинологических данных из рыхлых отложений пещеры Першинская-1 // Эколоия древних и традиционных обществ. Вып. 3. (Ред. Н.П. Матвеева). С. 30 – 36. (in Russian).

Lapteva EG (2008) Major palaeogeographical stages and specific landscape-climatic changes on the eastern slope of the Urals during the last 50 kyrs (inferred from palynological data) // Problems of Pleistocene palaeogeography and stratigraphy. (Eds. N.S. Bolikhovskaya and P.A. Kaplin). Vol. 2. P. 196 – 204. (in Russian).

Pisareva VV, Faustova MA (2008) Reconstruction of Landscapes of Northern Russia during the Middle Valday Mega-Interstadial // Way to North: Paleoenvironment and Inhabitants of Arctic and Subarctic (Eds. A.A. Velichko and S.A. Vasil'ev). Moscow.P. 53 – 62. (in Russian).
